# Exploring protective effect of *Glycine tabacina* aqueous extract against nephrotic syndrome by network pharmacology and experimental verification

**DOI:** 10.1186/s13020-020-00361-7

**Published:** 2020-08-01

**Authors:** Lihua Tan, Yanbei Tu, Kai Wang, Bing Han, Hongquan Peng, Chengwei He

**Affiliations:** 1 State Key Laboratory of Quality Research in Chinese Medicine, Institute of Chinese Medical Sciences, University of Macau, Taipa, 999078 Macao SAR China; 2 Renal Division, Kiang Wu Hospital, Macao, 999078 Macao SAR China

**Keywords:** *Glycine tabacina* aqueous extract, Network pharmacology, Nephrotic syndrome, Oxidative stress, Inflammation

## Abstract

**Background:**

*Glycine tabacina* (Labill.) Benth, one of the traditional Chinese herbal medicines, has been used for treatment of nephritis, osteoporosis, rheumatism, and menopausal syndrome. The aim of this study was to illuminate the therapeutic effect and mechanism of *Glycine tabacina* aqueous extract (GATE) in the treatment of nephrotic syndrome (NS).

**Methods:**

UHPLC-DAD-MS/MS was used to analyze the chemical profile of GATE. Adriamycin (ADR)-induced NS mouse model and network pharmacology methods were conducted to explore the protective effect and mechanism of GATE on NS treatment.

**Results:**

GATE administration significantly ameliorated symptoms of proteinuria and hyperlipidemia in NS mice, as evidenced by reduced excretion of urine protein and albumin, and decreased plasma levels of total cholesterol and triglyceride. Decreased blood urea nitrogen (BUN) and creatinine levels in NS mice suggested that GATE could prevent renal function decline caused by ADR. GATE treatment also inhibited ADR-induced pathological lesions of renal tissues as indicated by periodic acid Schiff staining. Six flavonoids of GATE were identified by using UHPLC-DAD-MS/MS. Network pharmacology analysis indicated that the protection of GATE in treating NS might be associated with the regulation of oxidative stress and inflammation. In addition, the in vivo experiment validated that treatment with GATE markedly decreased reactive oxygen species production, malonaldehyde level, and increased superoxide dismutase activity both in plasma and renal tissues. TNF-α level in plasma and protein expression in kidney were significantly decreased in GATE treatment groups.

**Conclusions:**

Combination of network pharmacology analysis and experimental verification revealed that GATE exerts anti-NS effect possibly through modulating oxidative stress and inflammation, suggesting the potential application of GATE or its derivatives in the prevention and treatment of NS and other related kidney diseases.

## Background

Nephrotic syndrome (NS) is a general term of various renal disorders which is manifested as massive proteinuria, hyperlipidemia, hypoalbuminemia and edema in clinic [1]. Various pathogenic factors, such as infection, lupus, diabetic nephropathy, drugs and cancer, can cause the nephrotic syndrome [2, 3]. According to its histological characteristics, NS can be classified into several types including minimal change disease, membranous proliferative glomerulo-nephritis, focal segmental glomerulosclerosis, and membranous nephropathy [4]. The annual incidence of NS has been estimated to be 3 per 100,000 adults and 2–7 per 100,000 children, posing an enormous burden on both society and individuals due to the high risk for the progression of end stage renal disease [5, 6]. The main therapeutic agents for NS are glucocorticoids, immunosuppressants and cytotoxic drugs. However, more than 10% of patients are steroid-resistant, and long-term use of steroids and some immunosuppressants may lead to serious side effects such as nephrotoxicity, bacterial infection, hyperglycemia, osteoporosis and dyslipidemia [6, 7].

The limited success of clinically available therapies for NS highlights the pressing requirement for the development of more effective and safer alternative agents. Thanks to the low side effects, rich resource and remarkable efficacy of traditional Chinese medicine (TCM), TCM, as one of the major modalities in complementary and alternative medicine, has a long history for treating a variety of nephropathies [6, 8, 9]. Network pharmacology is a systematic approach, which is used to understand the complexities among compounds, targets, diseases and biosystems [10]. The idea of this method conforms to the holistic and systemic views of TCM theory [11]. Nowadays, network pharmacology has been widely used to investigate the pharmacological action, safety and complex molecular mechanisms of TCM for treating various diseases, such as cardiovascular disorders, cancer, rheumatoid arthritis and chronic kidney disease [9, 12].

**Fig. 1:**
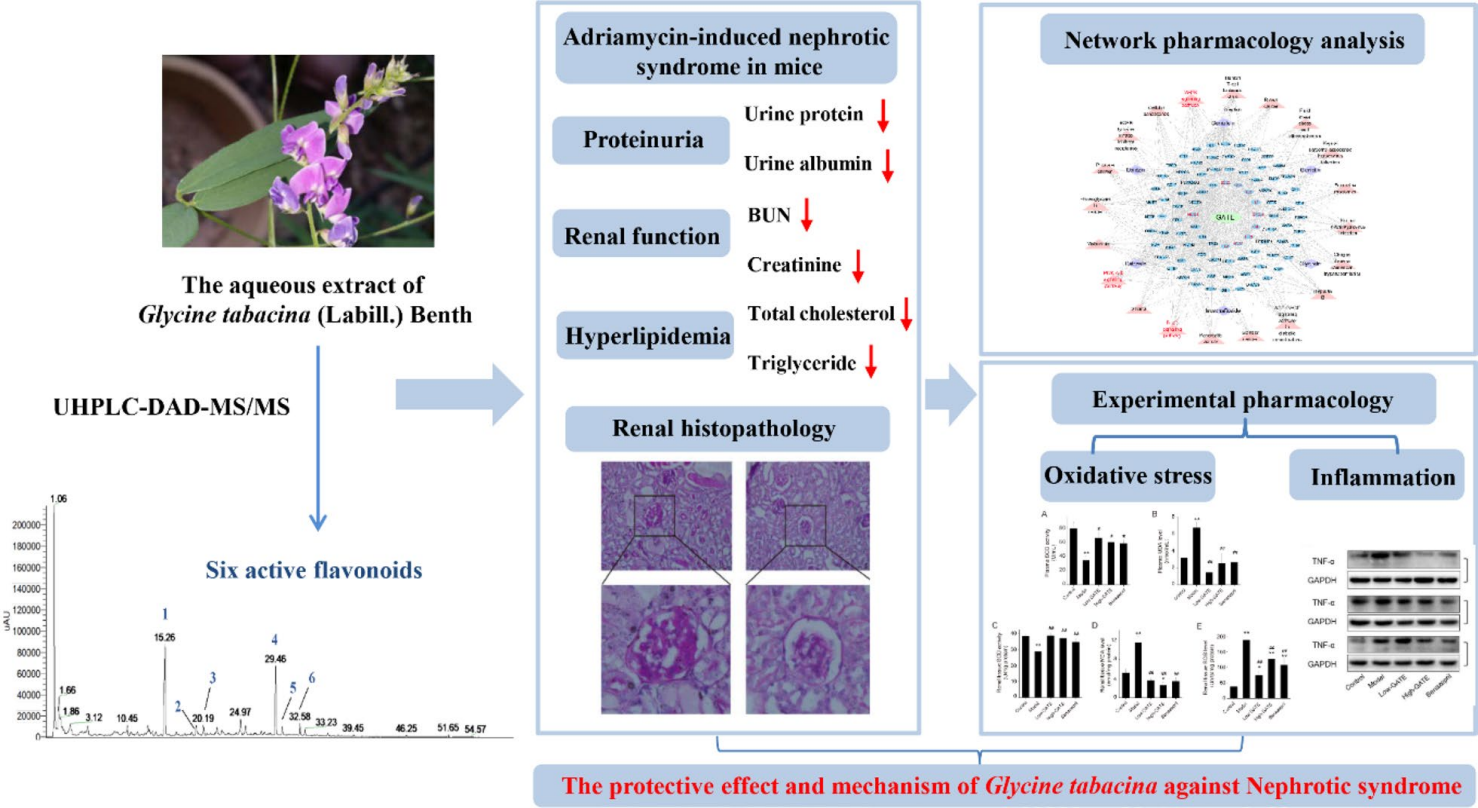
Scheme of the study

*Glycine tabacina* (Labill.) Benth, a well-known folk medicine and Chinese herbal medicine, is a leguminous plant which is mainly distributed in Australia, the Ryukyu Islands, southern China, and some South Pacific Islands. Its roots have been utilized for the treatment of osteoporosis, nephritis, rheumatism and menopausal syndrome in folk medicine [13, 14]. In addition, *G. tabacina* is also famous as one of the sources of the folk medicine ‘I-Tiao-Gung’ and serves as a herb tea in Taiwan [13]. However, few of phytochemical and pharmacological studies have been conducted and reported on this species [13, 15]. In vitro anti-inflammatory, anti-arthritic, antioxidant and antidiabetic activities of its extract have been reported [13, 16, 17]. Nevertheless, the therapeutic effect of *G. tabacina* aqueous extract (GATE) on NS is completely unknown. Therefore, in the current study, an adriamycin (ADR)-induced NS mouse model and a GATE-targets-pathways-NS network were used to investigate the potential effect of GATE on NS and reveal its preliminary mechanisms (Fig. 1). The results of in vivo experiments showed that GATE could alleviate the symptoms of heavy proteinuria, hyperlipidemia and renal function decline in NS mice. Subsequently, network pharmacology analysis revealed that the underlying mechanisms of GATE against NS were closely related to oxidative stress and inflammation, which have been validated by corresponding experiments. These results strongly suggest that GATE and its chemical constituents could be potential agents for the prevention or treatment of nephrotic syndrome.

## Methods

### Plant material and reagents

*Glycine tabacina* (Labill.) Benth, a well-known folk medicine, is recorded in http://www.theplantlist.org. The samples, authenticated by Dr. Jianping Han of Chinese Academy of Medical Sciences & Peking Union Medical College (Beijing, China) by a DNA barcoding method [18], was supplied by a local farmer (Fuqing, Fujian, China) and deposited in State Key Laboratory of Quality Research in Chinese Medicine, Institute of Chinese Medical Sciences, University of Macau (UM), Taipa, Macao SAR, China (voucher specimen: ICMS-2015-GT0012).

Adriamycin and benazepril·HCl were offered by Aladdin Inc. (Shanghai, China). PAS stain kit was provided by Leagene Biotechnology Co., Ltd. (Beijing, China). TNF-α antibody was supplied by Cell Signaling Technologies (Beverly, MA, USA). All other chemicals and reagents were of analytical grade.

### Preparation of GATE aqueous extract

The preparation of GATE aqueous extract was performed as follows. Briefly, the whole plant of *G. tabacina* (300 g) were powdered and then simmered with 3 L distilled water in a boiler at 90 °C for 30 min. The mixture was filtrated right after the simmer and the supernatants were collected, concentrated and lyophilized to obtain the lyophilized powder of GATE (32.2 g, 10.7% yield). Afterwards the powder was resuspended in water (150 mL) to acquire a stored suspension with a concentration of 2 g (raw herb)/mL for subsequent intragastric administration. The stock solutions were stored at − 80 °C until use. Moreover, a handful of powder was dissolved in methanol to prepare a 10 mg/mL GATE aqueous solution for the chemical composition analysis by UHPLC-DAD-MS/MS.

### Experimental animals

Male Balb/c mice (17–22 g) were provided by the Animal Facility of Faculty of Health Sciences at UM. Mice were maintained under controlled conditions of 22 ± 2 °C and 55 ± 5% humidity at a 12 h/12 h light/dark cycle, and provided with forage and tap water ad libitum. All mice were acclimatized for 7 days at experimental conditions before experiment. All procedures of animal care and animal experiments were authorized by the Animal Ethical and Welfare Committee of UM (Protocol ID: UMARE-046-2017) and conducted in compliance with *the Guide to Animal Use and Care of the UM*.

### Induction of NS and drug administration

In total 30 male balb/c mice were randomly divided into 5 groups (6 animals per group): normal group, untreated mice; model group, mice with NS; Low-GATE group, NS mice treated with GATE (2.5 g raw herb/kg body weight); High-GATE group, NS mice treated with GATE (5 g raw herb/kg); benazepril group, NS mice treated with benazepril (positive drug, 5 mg/kg). NS was induced by a single injection of ADR (11 mg/kg) through the tail vein of mice at day 0 [19], while mice in normal group were injected intravenously with saline only. Mice in drug-treated groups were administered with GATE or benazepril by oral gavage once daily for 25 days from day 0. The normal group and model group received equal volumes of water daily. The body weight was measured twice a week. The doses of GATE were set referring to our previous reports [16, 17] and the human dosage suggested by local herbalist. The endpoint of 25 days was chosen in the present study according to the results of our preliminary experiment and previous studies [19–21].

### Measurement of urine protein, urine albumin and urine creatinine levels

Twenty-four-hour urines of all mice were collected on day 0, 4, 7, 11, 18 and 25 after ADR injection. The levels of urine protein were measured by a BCA kit obtained from Thermo Fisher Scientific (Rockford, USA). The levels of urinary albumin and urine creatinine were measured by the corresponding assay kits obtained from Fosun Long March Medical Science Co., Ltd (Shanghai, China).

### Measurement of BUN, creatinine, total cholesterol and triglyceride levels in plasma

Blood samples were collected by retro-orbital puncture on day 0, 7, 14 and 25, and the plasma was separated by centrifugation at 3000 rpm for 15 min. All samples were frozen at − 80 °C until assayed. Levels of BUN, creatinine, total cholesterol and triglyceride in plasma were detected by the corresponding assay kits following the manufacturer’s instruction (Fosun Long March Medical Science Co., Ltd, Shanghai, China).

### Histopathological analysis

At the termination of experiment (day 25), all mice were euthanized. Right kidney was removed and immediately fixed in 10% phosphate-buffered formalin. Then the kidney tissues were dehydrated in alcohol and embedded in paraffin wax. Sections (4 μm) were obtained from the paraffin blocks and stained with PAS to evaluate the renal pathological changes. The pathological biopsies were examined under an Olympus CX41 light microscope (Olympus, Tokyo, Japan).

### UHPLC-DAD-MS/MS analysis of GATE

UHPLC-DAD-MS/MS analysis of GATE was implemented on the Dionex UltiMate 3000 Rapid Separation HPLC system (Thermo Fisher Scientific Inc., USA) coupled with a DAD-3000RS detector. An Agilent SB-C8 column (100 × 2.1 mm, 1.8 μm, Agilent, USA) was applied for chromatographic separation. The elution was conducted by using mobile phase A (water with 0.1% formic acid) and B (methanol) with the following gradient: 0–50 min, 5–80% B; 50–55 min, 80–100% B; 55–60 min, 100% B. The flow rate was 0.25 mL/min and UV spectra were recorded at 254 nm. For the ESI-HRMS analysis of GATE, LTQ-Orbitrap XL mass spectrometer (ThermoFisher Scientific, Bremen, Germany) fitted with an electro-spray ionization (ESI) source was used. The following experimental parameters setting were employed: ion spray voltage, 4 kV; tube lens voltage, − 67 V; capillary voltage, − 40 V; capillary temperature, 350 °C; auxiliary gas flow rate, 20 arbitrary units; sheath gas flow rate, 60 arbitrary units; and scan range, m/z 100–1500, collision energy, 35 eV. Mass spectra were automatic recorded in negative ion mode.

### Network pharmacology analysis of GATE against NS

#### Targets prediction of GATE against NS

The screened targets of GATE in the treatment of NS were obtained by the following protocol. All targets of compounds identified from GATE were obtained from the following four databases: PharmMapper [22] (z score > 1.5, http://www.lilab-ecust.cn/pharmmapper/), Stitch [23] (confidence score > 0.5, http://stitch.embl.de/, ver. 5.0), SwissTargetPrediction [24] (probability > 0.5, http://www.swisstargetprediction.ch/), and the Traditional Chinese Medicine Systems Pharmacology (TCMSP) Database [25] (http://tcmspw.com/tcmsp.php).

The data for the NS-related targets were screened from DisGeNET database [26] (score > 0.1, https://www.disgenet.org/home/), Genecards database [27] (relevance score > 5, https://www.genecards.org/), and Online Mendelian Inheritance in Man [28] (OMIM, https://omim.org/). The species was limited to “Homo sapiens”, and the names of the selected target proteins were converted to its official symbol according to the Uniprot website (https://www.uniprot.org/). All used dataset was collected by databases on December 17, 2019.

After the targets of GATE and NS from these databases were amalgamated, Draw Venn Diagram website (http://bioinformatics.psb.ugent.be/webtools/Venn/) was employed to analyze the intersection of GATE targets and NS targets. And these overlapping targets were considered as the therapeutic targets of GATE for NS treatment and used for subsequent data analysis.

#### Protein–protein interaction (PPI) analysis

The protein–protein interaction of the overlapping targets between GATE and NS was analyzed by using STRING [29] database (Version 11.0, https://string-db.org/). In STRING, the organism was set as “homo sapiens”, and the confidence score > 0.7 was chosen. Then, the PPI network of the overlapping targets between GATE and NS was constructed by Cytoscape 3.7.2 software. Meanwhiles, the degree centrality of each node, which reflects the combined number of targets, was calculated by analyzer plugin of Cytoscape. All nodes in the network represented predicted targets and the targets with highest degree values of the topology parameter represented the hub targets.

#### Enrichment analysis

To explore the role of predicted targets connected with GATE against NS, ClueGO and CluePedia, two plugins of Cytoscape, were used to perform Gene Ontology (GO) enrichment analysis of overlapping targets [30–32]. In ClueGo, Two-side hypergeometric test was used to evaluate the significance of each term. A Kappa score of 0.4 or 0.7 and an adjusted *p* value of 10^−2^ or 10^−6^ were set as threshold values. The functionally grouped networks with the nodes grouped and connected based on Kappa score were visualized by Cytoscape 3.7.2 software.

In addition, to explore the biological pathways conducted by GTAE for NS treatment, ClusterProfiler package of R3.6.2 was employed to perform KEGG pathway enrichment analysis of overlapping targets [33]. The GATE-related pathways were screened based on *p *< 0.01. And the top 20 KEGG pathways were shown by bubble map.

#### Construction of GATE-targets-pathways-NS network

The network of interactions among NS, targets, pathways and chemical components of GATE (abbreviated as GATE-targets-pathways-NS network) was built using Cytoscape 3.7.2 software to analyze the complex interactions between GATE and NS. In the network, ellipse, quadrangle and triangle nodes represent targets, chemical components and pathways, respectively. While edges stand for the interactions among chemical components of GATE, NS, targets and pathways.

### Experimental verification

#### Determination of renal oxidative stress

The mice were anesthetized and sacrificed on day 25, and the left kidney was removed and then homogenized as mentioned anteriorly [34]. The SOD activity and MDA level in plasma and kidney homogenate were detected according to the protocols of corresponding assay kit from Nanjing Jiancheng Bioengineering Institute (Nanjing, China), respectively. The ROS level in kidney homogenate was determined by a commercial ROS assay kit following manufacturer’s protocols (GENMED Scientifics, USA).

#### Measurement of TNF-α levels in plasma

Plasma TNF-α level on day 25 was determined using Enzyme-linked immuno-sorbent assay (ELISA) kit offered by Biolegend, Inc. (San Diego, USA).

#### Western blot analysis

Total proteins were obtained from kidney homogenates with RIPA buffer supplemented with phenylmethylsulfonyl fluoride and protease inhibitor cocktail. Equivalent protein of each sample (20 μg) was separated on 12% SDS-polyacrylamide gel by electrophoresis, and then transferred to PVDF membranes. After blocking with 5% skim milk in Tris-buffered saline/Tween 20 for 1 h, the membranes were incubated with TNF-α primary antibody and horseradish peroxidase conjugated secondary antibody following the protocol described previously [16]. The membranes were treated according to the protocol of the enhanced chemiluminescence detection kit (Bio-Rad, USA) and protein bands were observed by Bio-Rad ChemiDocTM MP Imaging System (Hercules, CA, USA).

### Statistical analysis

GraphPad Prism 5.0 (San Diego, USA) was utilized to conduct all statistical analyses in this study. Significant differences between multiple groups were assessed using a one-way ANOVA followed by Tukey’s post hoc test, while differences of two categorical variables on one continuous variable were evaluated by two-way ANOVA followed by Bonferroni test. Data were presented as mean ± standard error of the mean (S.E.M.), and *p* < 0.05 was regarded as statistically significant.

## Results

### Effects of GATE on body weight, kidney weight and proteinuria in NS mice

**Fig. 2:**
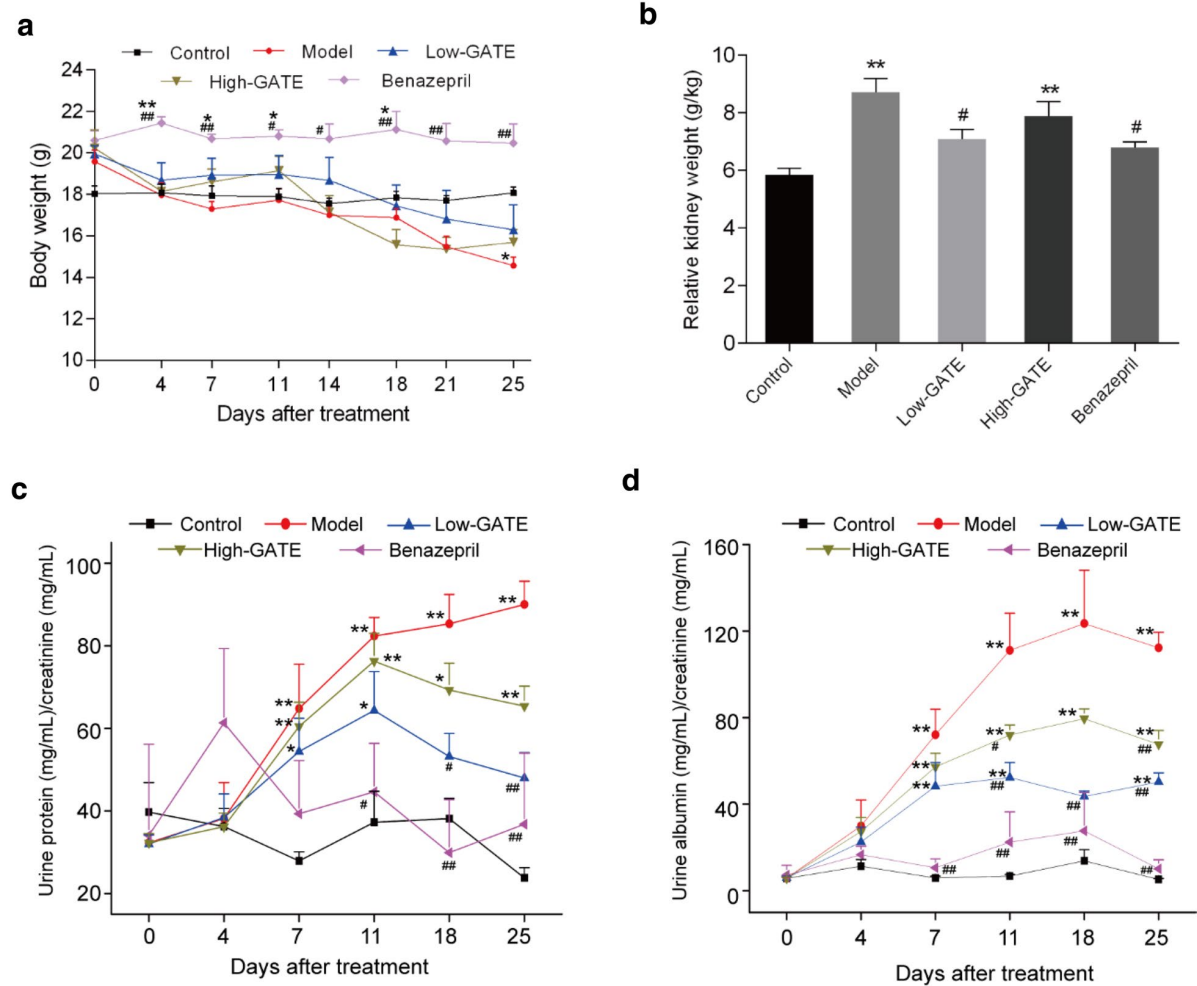
Effect of GATE on proteinuria in ADR-induced NS mice. Mice were orally administrated with GATE (2.5 and 5 g raw herb/kg body weight) or benazepril (5 mg/kg) once daily starting from day 0. Control and model group were given an equal volume of water. **a** Body weight was observed twice a week. **b** Relative kidney weight was determined at the end of the experiment. The excretion of 24-h urine protein (**c**) and urine albumin (**d**) was measured on day 0, 4, 7, 11, 18 and 25. The data were expressed as means ± SEM. *p < 0.05, **p < 0.01 compared with control group; ^#^*p* < 0.05, ^##^*p* < 0.01 compared with model group

As shown in Fig. 2a, mice in model group showed obvious loss of body weight compared with control group after ADR injection, which was significantly prevented by administration of GATE and benazepril. The increased kidney weight has been reported to be correlated with kidney tissue damage [35]. In the present study, the relative kidney weight of NS mice significantly increased versus control group at the end of experiment, but this increase was weakened by the treatment of benazepril and 2.5 g raw herb/kg body weight GATE (Fig. 2b).

Proteinuria is a main indicator for various renal diseases. To evaluate the therapeutic effect of GATE on proteinuria, the 24 h-urines of all mice were collected and then the levels of urine protein and urine albumin were determined. As expected, mice in model group developed severe proteinuria as indicated by notable increases in the excretion of urine protein and albumin from day 7 to day 25 compared with control group (*p* < 0.01). Oral administration of GATE at different doses (2.5 and 5 g raw herb/kg body weight) and benazepril markedly decreased the level of urine protein and albumin during the experiment as compared to model group (Fig. 2c, d), indicating that GATE treatment significantly alleviated the ADR-induced proteinuria in NS mice.

### Effects of GATE on renal function in NS mice

**Fig. 3:**
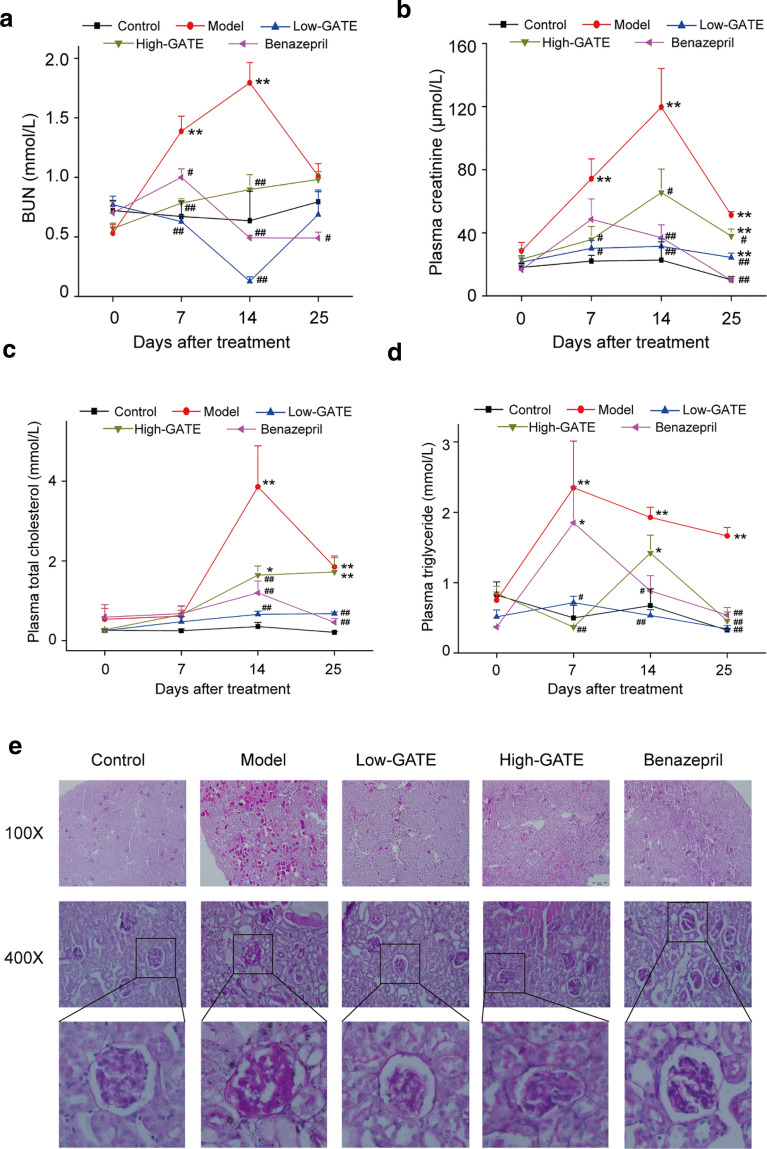
Effect of GATE on renal function, hyperlipidemia and renal histopathological changes in ADR-induced NS mice. Blood was collected on day 0, 7, 14 and 25 after ADR injection. Then plasma levels of BUN (**a**), creatinine (**b**), total cholesterol (**c**) and triglyceride (**d**) were determined using the corresponding assay kits. The data were expressed as means ± SEM. *p < 0.05, **p < 0.01 compared with control group; ^#^*p* < 0.05, ^##^*p* < 0.01 compared with model group. At the end of experiment, mice were sacrificed and the right kidney was removed for histological analysis (**e**)

Plasma BUN and creatinine are two important indicator indexes for renal function [36], thereby their plasma levels were determined to evaluate the therapeutic effect of GATE on the decline of renal function induced by ADR (Fig. 3a, b). The data showed that injection of ADR at 11 mg/kg remarkably increased the plasma levels of BUN and creatinine in model group when compared to control group. However, obvious decreases of BUN level were observed in Low-GATE, High-GATE and benazepril treatment groups. Oral administration of GATE and benazepril also significantly reduced the plasma creatinine levels from day 7 to day 25 compared to model group. The results suggested that GATE treatment could significantly ameliorate ADR-induced renal function decline in NS mice.

### Effects of GATE on hyperlipidemia in NS mice

Hyperlipidemia is one of the primary pathological characters in ADR-induced renal diseases [37]. As shown in Fig. 3c, d, the NS mice injected with ADR showed elevated plasma levels of total cholesterol and triglyceride in comparison with control group (*p* < 0.01), which indicated the occur of hyperlipidemia in model group. However, the increase of total cholesterol level was significantly inhibited by treatment with GATE. The dramatic reduction in triglyceride level was also observed in GATE and benazepril groups from day 7 to day 25 compared to model group. Notably, the efficacy of Low-GATE treatment on ADR-induced hyperlipidemia was better than that of positive drug, benazepril.

### Effects of GATE on renal histopathological changes in NS mice

Therapeutic effects of GATE treatment on ADR-induced histopathological alterations of kidney were evaluated by staining with PAS. As shown in Fig. 3e, control group showed normal tubulointerstitial and glomerular architectures in the renal cortex. In contrary, it was clearly found that NS mice in model group showed typical renal pathologic alterations with tubulointerstitial lesions and glomerular injury, e.g. hyperemia, thickened glomerular basement membrane, mesangial expansion, increased accumulation of mesangial matrix, etc. However, these histopathological indications were significantly ameliorated after treatment with Low-GATE or High-GATE, which were consistent with the results of renal function. The results demonstrated that GATE could moderate the renal injury in ADR-induced NS mice.

### Chemical composition analysis of GATE

**Fig. 4:**
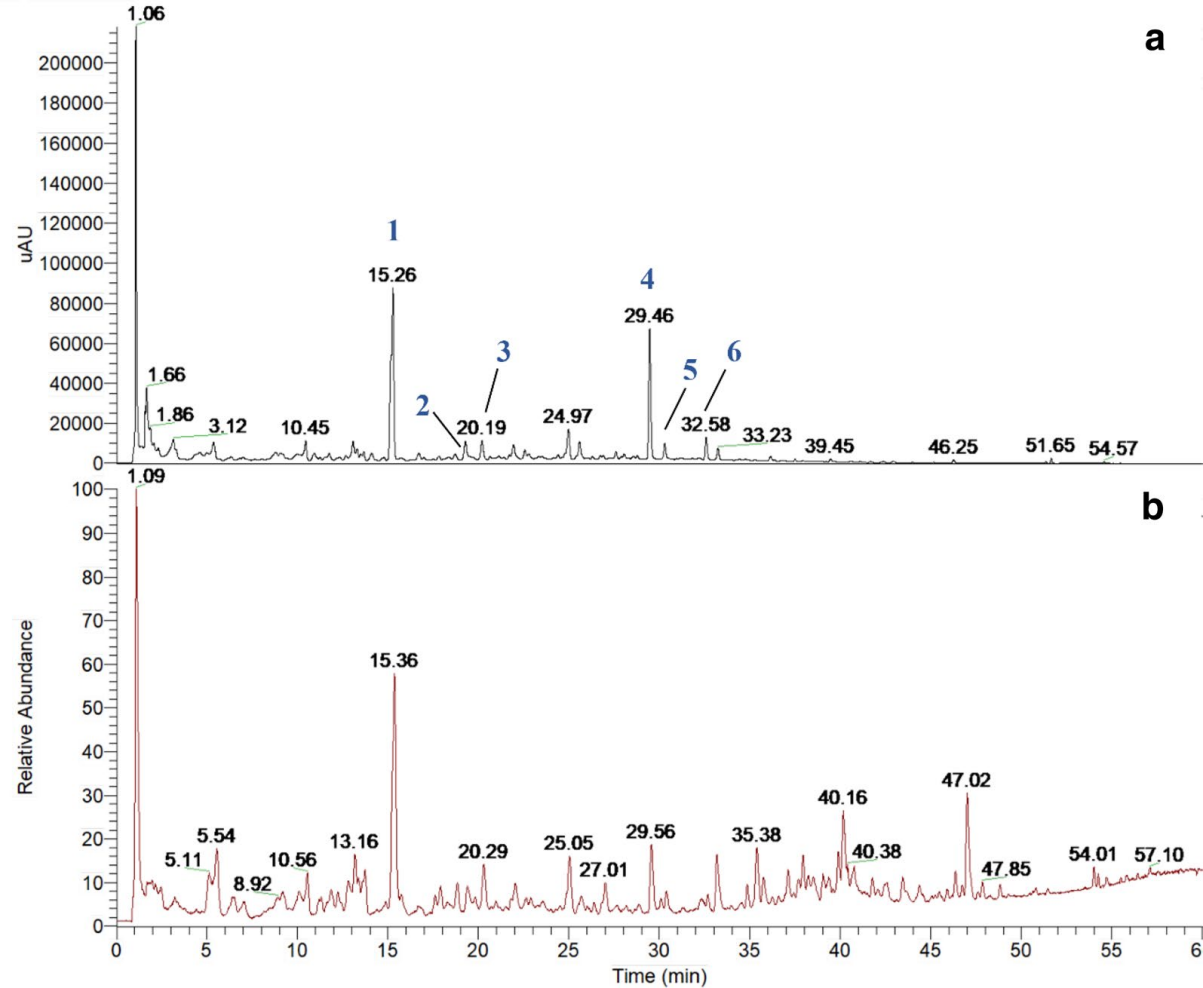
UHPLC-UV chromatogram (**a**) and total ion chromatogram (**b**) of GATE. The compounds were identified as genistin (1), daidzin (2), isoschaftoside (3), daidzein (4), glycitein (5), genistein (6), respectively

The UHPLC-UV chromatogram and total ion chromatogram of GATE were shown in Fig. 4. Six major peaks were observed in the UHPLC-UV chromatograms of GATE at 254 nm (Fig. 4a), and their retention time, UV absorption maxima, molecular formula, experimental [M−H]^−^, theoretical [M−H]^−^, delta and main MS^2^ fragment ions were summarized in Table 1. By comparison with the exact mass, MS^2^ fragment information and UV spectra of previous reports and databases [38–40], 6 peaks recorded in chromatograms were deduced as genistin, daidzin, isoschaftoside, daidzein, glycitein, and genistein.

**Table 1:** Identification of the chemical components in GATE by UHPLC-DAD-MS/MS analysis (negative ion mode)

Peak	Retention time (min)	Experimental [M−H]^−^ (m/z)	Theoretical [M−H]^−^ (m/z)	Delta (ppm)	Main MS^2^ fragments	UV max. (nm)	Molecular formula	Proposed compound
1	15.26	431.09823	431.09837	− 0.325	369.18481, 329.13257, 269.13925, 203.05310	260	C_21_H_20_O_10_	Genistin
2	19.31	415.10405	415.10346	1.421	253.10510	255	C_21_H_20_O_9_	Daidzin
3	20.19	563.14050	563.14063	− 0.228	503.28558, 473.20331, 443.26880, 383.15466, 353.13293	273, 334	C_26_H_28_O_14_	Isoschaftoside
4	29.46	253.05037	253.05063	− 1.036	224.17744, 209.02998, 197.07283, 134.95865	250, 303	C_15_H_10_O_4_	Daidzein
5	30.30	283.06104	283.06120	− 0.554	268.09027	255	C_16_H_12_O_5_	Glycitein
6	32.58	269.04538	269.04555	− 0.619	241.05565, 225.15002, 181.10497	261	C_15_H_10_O_5_	Genistein

### Network pharmacology analysis

#### Identification of overlapping targets for GATE against NS

**Fig. 5:**
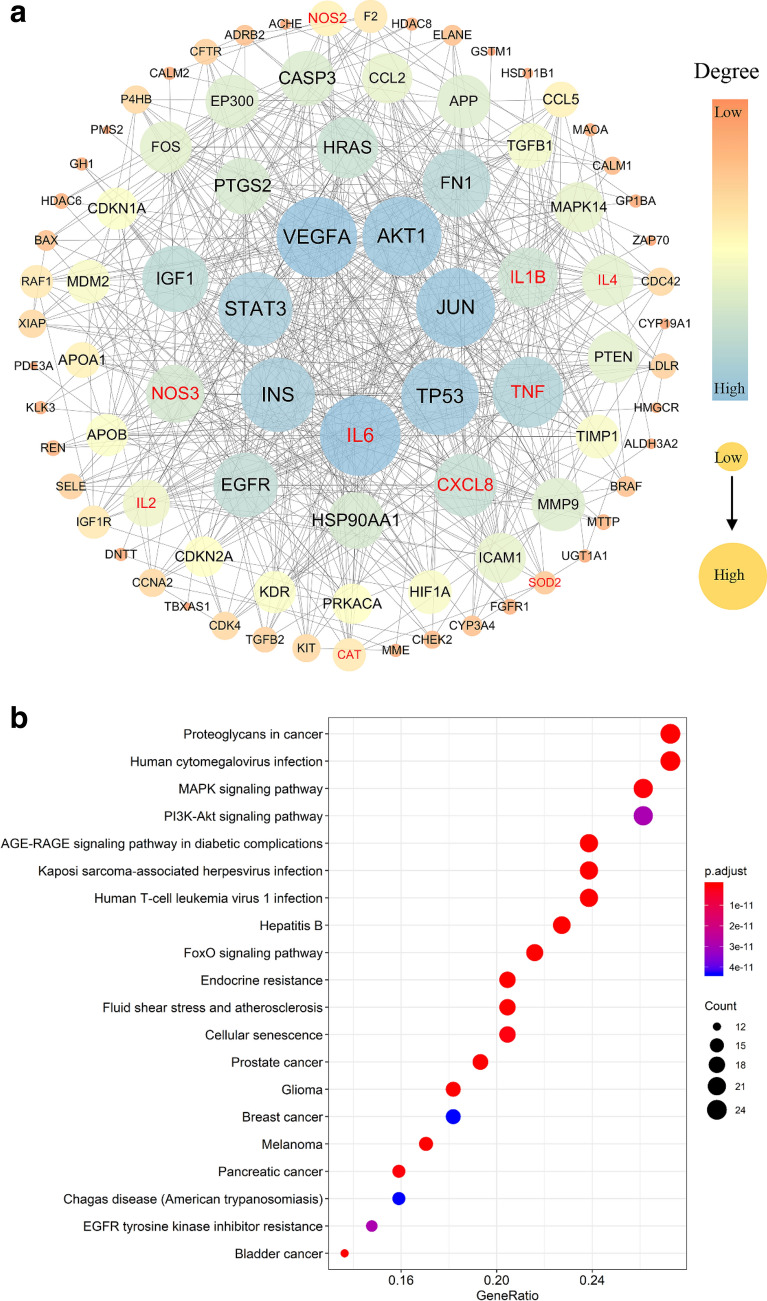
PPI network (**a**) and KEGG enrichment analysis (**b**) of GATE targets against NS. **a** Nodes in the network indicate proteins, where edges refer to the protein–protein interactions. The size and different colors of the circle represent to the degree. Number of nodes: 92; number of edges: 589; average degree: 12.8. **b** The y-axis of bubble map shows significantly KEGG pathways of the overlapping targets, and the x-axis is the GeneRatio. The higher GeneRatio indicates the more targets belonging to a specific pathway. Size of spot indicates the counts of targets and color reflects the different adjusted *p* value

Based on the databases, 1806 NS-related targets were collected (Additional file 1: Table S1), and 280 corresponding targets of six flavonoids contained in GATE were identified (Additional file 1: Table S2). As shown in Additional file 1: Table S3, 92 overlapping targets were found by comparing the targets of GATE and NS, which were identified as the therapeutic targets of GATE for NS treatment. And the interaction of 92 overlapping targets analyzed by STRING database were visualized by Cytoscape 3.7.2. As shown in Fig. 5a, the PPI network of the overlapping targets included 92 nodes and 589 edges with an average degree of 12.8, where the edges represented the interactions between two proteins. After the degree value of each targets was calculated, 8 hub targets with a degree of more than two and a half times of the average node degree were selected and considered as the pivotal markers of GATE treatment on NS. Concretely Speaking, the 8 hub targets are as follows: IL-6, VEGFA, AKT1, JUN, TP53, INS, STAT3, TNF. In addition, some of the targets, highlighted in Fig. 5a, were associated with oxidative stress and inflammation, such as IL-6, TNF, NOS3, CXCL8, IL-1β, IL-4, IL-2, NOS2, SOD2 and CAT.

#### GO and KEGG enrichment analysis for GATE against NS

To understand the role of overlapping targets of GATE and NS in depth, GO and KEGG enrichment analyses were performed. GO enrichment analysis were illustrated based on the cell component (CC), biological process (BP), and molecular function (MF) terms. As Additional file 1: Tables S4–S6 showed, 202 BP, 5 CC, and 51 MF terms with an adjusted *p* value of 10^−2^ or 10^−6^ were enriched for the overlapping targets. Depending on the GO enrichment results, genes enriched in BP were mostly involved in positive regulation of cell adhesion, neuroinflammatory response, neurotransmitter metabolic process, ameboidal-type cell migration, cell cycle arrest, positive regulation of kinase activity, nitric-oxide synthase activity, alcohol metabolic process, hormone secretion and cellular response to oxidative stress (Additional file 1: Fig. S1). The enriched MF terms were dominated by positive regulation of kinase activity, kinase regulator activity, nitric-oxide synthase regulator activity, nitric-oxide synthase activity, monooxygenase activity, positive regulation of MAP kinase activity, cysteine-type endopeptidase activity involved in apoptotic process, positive regulation of DNA-binding transcription factor activity, regulation of lipase activity and heat shock protein binding (Additional file 1: Fig. S2). The highly enriched terms in CC included platelet alpha granule lumen, clathrin-coated vesicle membrane, low-density lipoprotein particle, clathrin-coated endocytic vesicle membrane and peroxisomal membrane (Additional file 1: Fig. S3).

In addition, 141 pathways of GATE on NS treatment were enriched by KEGG pathway analysis (adjusted *p* < 0.01). Detailed pathway information was presented in Additional file 1: Table S7. Based on the GeneRatio values and the adjusted *p* value, top 20 significantly pathways of GATE on NS, including MAPK signaling pathway, proteoglycans in cancer, Hhuman cytomegalovirus infection, AGE-RAGE signaling pathway in diabetic complications, endocrine resistance, glioma, FoxO signaling pathway, Kaposi sarcoma-associated herpesvirus infection, prostate cancer, Melanoma, fluid shear stress and atherosclerosis, bladder cancer, human T-cell leukemia virus 1 infection, hepatitis B, cellular senescence, pancreatic cancer, EGFR tyrosine kinase inhibitor resistance, PI3K–Akt signaling pathway, breast cancer and Chagas disease (American trypanosomiasis), were shown in Fig. 5b.

#### Construction of GATE-targets-pathways-NS network

**Fig. 6:**
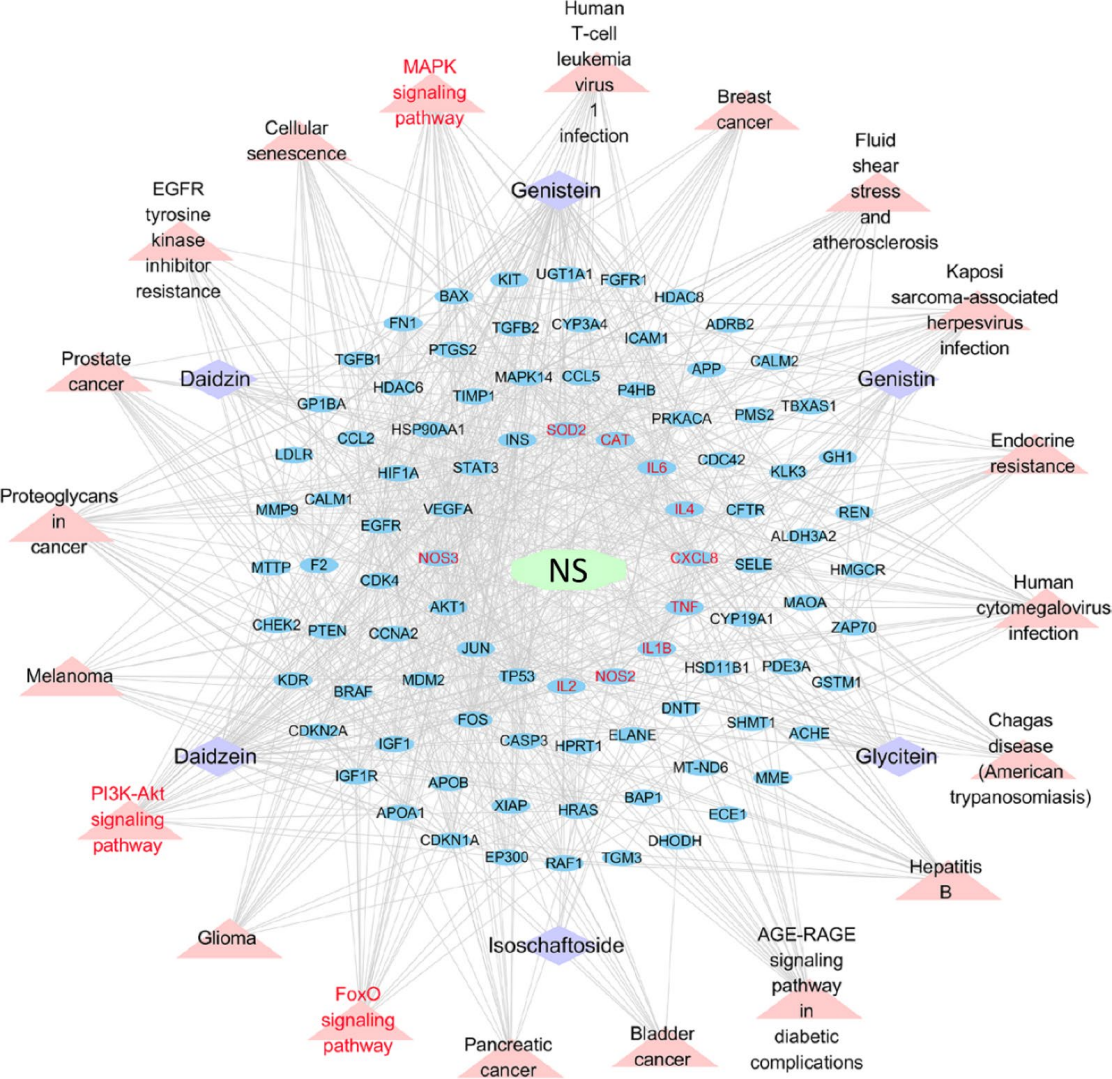
A “GATE-targets-pathways-NS” network built by Cytoscape 3.7.2. In the network, ellipse, quadrangle and triangle nodes represent targets, chemical components and pathways, respectively. While edges stand for the interactions among chemical components of GATE, NS, targets and pathways

To clarify the molecular mechanism of GATE for treating NS, the GATE-targets-pathways-NS network was built by Cytoscape 3.7.2. As shown in Fig. 6, the network consisted of 120 nodes (1 Chinese medicines, 6 compounds, top 20 signaling pathways, 92 targets, 1 disease) and 1208 edges. The detailed network information was shown in Additional file 1: Table S8. According to this network, the mechanism of GATE against NS was preliminary illuminated, suggesting the multi-targets and multi-pathways effect of GATE on the emergence and development of NS.

### Experiment validation

#### Effects of GATE on oxidative stress in NS mice

**Fig. 7:**
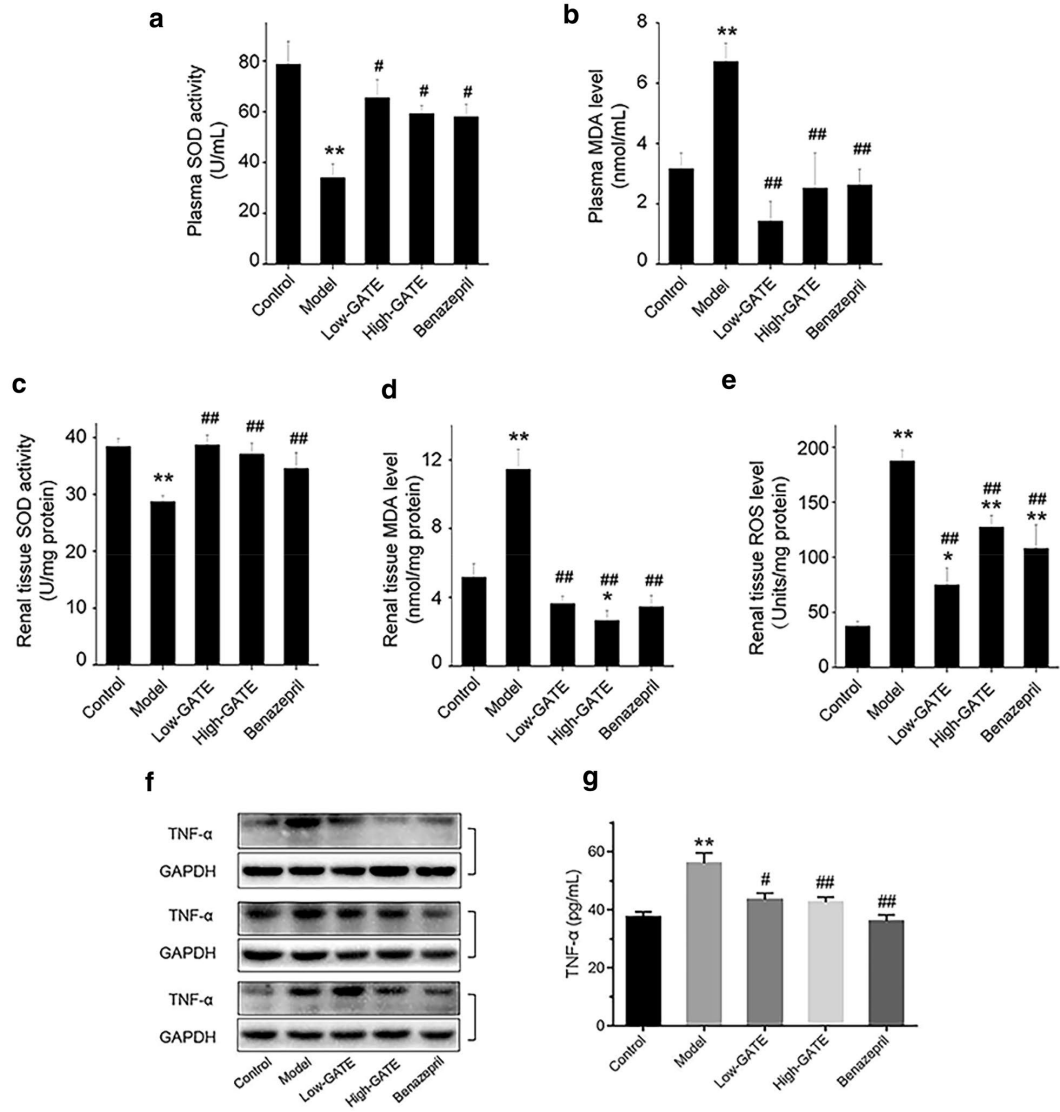
Effect of GATE on oxidative stress and TNF-α expression in ADR-induced NS mice. At the end of experiment, blood and left kidneys of mice from different groups were collected. Then plasma SOD activity (**a**) and MDA level (**b**), renal tissue SOD activity (**c**), MDA level (**d**) and ROS level (**e**) were measured using the corresponding assay kits. **f** Representative Western blots of TNF-α expression in kidney tissues from different groups on day 25. **g** The plasma TNF-α level of mice in each group was determined by ELISA on day 25. The data were expressed as means ± SEM. *p < 0.05, **p < 0.01 compared with control group; ^#^*p* < 0.05, ^##^*p* < 0.01 compared with model group

In line with previous reports [41, 42], the network pharmacology analysis indicated that oxidative stress plays a vital role in the etiopathogenesis of ADR-induced NS. To validate whether GATE showed anti-oxidative effects on ADR-induced NS, the SOD activity and MDA level were determined in plasma and renal tissue of mice in the present study (Fig. 7a–d). Compared with control group, ADR injection resulted in an oxidative stress as indicated by the diminished SOD activity and increased MDA level in the plasma and renal tissue of model group (all *p* < 0.01). Nevertheless, these detrimental effects related to NS condition were found to be greatly ameliorated by GATE treatment. Oral administration of Low-GATE, High-GATE or benazepril significantly increased the SOD activities in plasma and renal tissue compared to the model group (*p* < 0.05). Moreover, ADR-induced increases in tissue and plasma levels of MDA were markedly attenuated in GATE or benazepril treatment groups. ROS have been emerged as a principal mediator in the progression of NS induced by ADR [43]. Therefore, we next determined the ROS levels of renal tissue in all groups (Fig. 7e). As a result, ROS generation was markedly elevated in model group versus control group, which was significantly weakened by GATE or benazepril treatment (*p* < 0.01 versus model group). These results indicated that GATE might exhibit its anti-NS effect by alleviating oxidative stress in ADR-induced NS mice.

#### Effects of GATE on renal inflammation in NS mice

Along with the results of network pharmacology analysis, chemokines and cytokines, in particular TNF-α, have been reported to be critical for inflammatory responses and subsequent tissue injury in nephritis [37]. The results of ELISA assay demonstrated that there was an increased plasma level of TNF-α in model group compared to control group at day 25 (*p* < 0.01), while GATE and benazepril groups showed significant decrease in TNF-α level compared to model group (*p* < 0.05) (Fig. 7g). In addition, TNF-α protein level in renal tissue was also dramatically suppressed by GATE treatment in contrast to model group, as evidenced by western blot analysis (Fig. 7f). These results suggested that GATE could effectively suppress the inflammation in ADR-induced NS mice.

## Discussion

Nephrotic syndrome refers to series renal dysfunctional syndromes which is frequently seen in both children and adults [2]. It severely threatens the health and life quality of patients, but remains therapeutic challenge due to the limited effectiveness of currently available drugs [3]. Medicinal herbs and their active compounds as the rich sources for novel drug discovery and treatment of kidney diseases have attracted more attention in recent years [6, 8]. Thus, inspired by the folk remedy of *G. tabacina* in the treatment of nephritis, we evaluated the anti-NS effect of *G. tabacina* aqueous extract in ADR-induced NS mice, and explored its potential underlying mechanisms by the means of network pharmacology and experimental validation. To the best of our knowledge, it is the first work to reveal GATE could significantly alleviate the symptoms of heavy proteinuria, hyperlipidemia and renal function decline with reduced oxidative stress and inflammatory status in NS mice, suggesting its potential on the prevention and treatment of NS or other relative kidney diseases.

Heavy proteinuria is a main clinical manifestation of NS. Although there are various different causes of NS, they all share a common pathophysiology: massive loss of protein in the urine caused by damage of glomerular filtration barrier [44]. Therefore, proteinuria is a most common clinical predictor of glomerular diseases including NS, which indicates the progress of glomerular damage and plays a key role in the subsequent oxidative stress, inflammatory response and fibrosis, eventually giving rise to the development of end-stage renal disease [36]. In ADR-induced NS, ADR can increase glomerular permeability and directly injure the podocytes, resulting in heavy proteinuria [45]. In the present study, mice were observed for the weight loss, movement hysteresis and varying degrees of reduced water and food intake after ADR injection. Most importantly, the excretion of urine protein and urine albumin was progressively increased from day 0 to day 18 after ADR induction, confirming successful establishment of NS model in mice. However, in all GATE treatment groups, notable reductions on urine protein and albumin excretion were detected from day 7 compared with model group, which was accompanied with improved food intake, movement, and reduced weight loss. These results indicated that oral administration of GATE ameliorated symptom of proteinuria which was attributed to ADR-induced glomerular injury in NS mice.

Urea and creatinine, the breakdown products of protein metabolism and creatine respectively, are removed from the body by kidneys. However, they will be retained in the body due to the dysfunction of glomerular filtration function, leading to an increase in plasma levels of BUN and creatinine [46]. Therefore, BUN and creatinine levels are two specific markers to ascertain the renal function of patients in clinic [36]. Hyperlipidemia, which is characterized by hypercholesterolemia and increased triglyceride, is another major feature of ADR-induced NS [42]. Increasing evidences suggest it is an important pathogenic factor for kidney damage and may conduce to the development of ADR-induced renal diseases [37, 47]. Moreover, hyperlipidemia is thought to play a significant role in the deterioration of renal function [48]. In this study, mice in model group showed notable increases in the plasma BUN, creatinine, total cholesterol and triglyceride after ADR injection. However, these increases were significantly weakened in all GATE treatment groups, indicating oral administration of GATE ameliorated symptom of renal function decline and hyperlipidemia induced by ADR in NS mice. In addition, histological studies revealed that ADR caused significant damages of renal tissues in model group, including tubular damages, glomerular deformation, asymmetric mesangial proliferation and mesangial expansion. GATE treatment lessened ADR-induced histopathological lesions, suggesting its protective effect in ADR-induced renal injury.

Flavonoids which are abundant in various medicinal herbs and food sources are well-known for their protective roles in renal disorders such as diabetic nephropathy, glomerulonephritis and kidney insufficiency [49]. High total polyphenols content and total flavonoids content have been reported in aqueous extract of *G. tabacina* and four common dietary flavonoids (daidzein, genistein, daidzin and genistin) were identified by HPLC analysis [13]. In the present study, we revealed that six flavonoids, i.e. genistin, daidzin, isoschaftoside, daidzein, glycitein, genistein, were the major components in GATE using a UHPLC-DAD-MS/MS method. Therefore, the therapeutic effect of GATE on NS might be attributed to the presence of these flavonoids and unrevealed flavonoids in this species.

Network pharmacology is a systematic approach which emphasizes the integration of systems biology, bioinformatics, and pharmacology [10]. This method interprets the underlying complex relationships among diseases, components and biological systems [50, 51]. Nowadays, network pharmacology is deemed to as be a powerful tool which widely applied in effective drug discovery and mechanism study of TCM on complex diseases treatment [52]. In the current study, network pharmacology was applied to comprehensive understand the relationships between NS treatment and GATE administration. The data revealed that 92 targets were closely bound up with GATE on NS, in which IL6, VEGFA, AKT1, JUN, TP53, INS, STAT3, TNF were considered as center targets. It has been reported that VEGFA, AKT1, JUN, TP53, STAT3 and INS were closely bound up with the pathogenesis of various nephropathies, and the development and function of kidney [53–58]. In addition to the targets mentioned above, other targets, such as CXCL8, IL1β, NOS3, IL-4, IL-2, NOS2, CAT and SOD2, were involved in the pathogenic process of NS through modulating oxidative stress and inflammatory response. Herein, SOD2 and CAT, separately encoded the antioxidant enzyme superoxide dismutase and catalase, were known as one of the cellular defense mechanisms against free radicals in the development process of NS [46]. NOS2 and NOS3, separately encoded the inducible nitric oxide synthase (iNOS) and endothelial nitric oxide synthase (eNOS), are involved in the synthesis of the nitric oxide (NO) and considered as a significant nephroprotective factor [59]. Interleukin, the multifunctional inflammatory cytokines are closely associated with various renal diseases and the injury of kidney residential cells, such as IL-6, CXCL8, IL-1β, IL-2 and IL-4 [60–64]. TNF-α, an important regulatory factor, which can promote the production of IL-6, IL-1β, and other cytokines, participates in inflammation, oxidative stress and damage in various renal diseases [65].

Furthermore, 20 KEGG pathways were markedly enriched by KEGG enrichment analysis, including MAPK signaling pathway, PI3K–Akt signaling pathway and FoxO signaling pathway. MAPK signaling pathways, activated by peptide growth factors, cytokines and oxidative stress, modulate multiple cellular processes including immune response, inflammation, oxidative stress, and various signal transduction [66, 67]. In recent years, evidences showed that MAPK signaling pathways, are strongly linked renal injury, renal oxidative stress and glomerular disease, p38MAPK signaling pathway in particular [68, 69]. The PI3K/Akt signaling pathway, activated by integrins, growth factors and cytokines, is crucial for the functional integrity of podocytes, podocyte apoptosis, renal fibrosis and the progression of renal disease [70, 71]. The FoxO signaling pathway involves in the maintenance of homeostasis, including immune responses, oxidative stress responses, inflammation, cell cycle progression and apoptosis [72]. The FoxO signaling pathway, regulated by oxidative stress and PI3K–AKT signaling pathway, was thought to be one of the mechanisms for the treatment of kidney diseases [73]. Moreover, others pathways notably enriched in this study were thought to be connected with the occurrence of NS, such as some pathways related to cancer and viral [74, 75]. According to the results of network pharmacology, we found that multiple pathways and multiple targets were involved in the modulation of GATE on NS. Meanwhile, we noticed that the predicted targets and significantly pathways of GATE on NS were closely bound up with inflammation and oxidative stress. To a degree, this is in accordance with the pathogenic mechanism of NS.

It is generally recognized that inflammation and oxidative stress are central pathogenic factors in the progression of ADR-induced NS [34, 37]. ADR has been reported to result in direct oxidative damage to DNA and induce generation of lipid peroxidation [41]. Metabolism of ADR in kidneys leads to the excessive production of free radicals, ROS and NO, and decrease of antioxidases, which contributes to the progress of renal oxidative stress [41, 76]. Meanwhile the increased oxidative stress caused by ADR further induces the generation of various inflammatory cytokines and chemokines, leading to renal inflammation and subsequent kidney injury [76, 77]. Therefore, combined with the results of network pharmacology, we speculate that the potential mechanism of GATE against NS may be due to its coordinated modulation of oxidative stress and inflammation.

In this study, as expected, the intracellular ROS production was markedly enhanced in the renal tissue of ADR-injected mice compared with control group, whereas GATE treatment significantly reversed this change induced by ADR. Moreover, the elevated level of MDA, the end product of lipid peroxidation, was observed both in plasma and renal tissues of model group, suggesting the increased lipid peroxidation in ADR-induced NS mice. Oral administration of GATE prominently reduced the MDA levels in comparison with model group. Antioxidases are the first line to protect organisms from free radicals. In particular, SOD functions as an important enzyme for the removal of superoxide ion and ROS [78]. In model group, the reduced SOD activity in plasma and renal tissues was found under NS condition, which was restored by GATE treatment. These results indicated that GATE could protect mice from renal injury through alleviating ADR-induced oxidative stress in NS mice, which were in accordance with those of network pharmacology.

TNF-α, mainly produced by renal mesangial, tubular epithelial cells and macrophages, plays a key role in renal damage as it not only exerts direct effect on renal cells but also induces the generation of ROS, NOS and other inflammatory mediators, thereby amplifying the inflammatory response [46, 79, 80]. Anti-TNF-α blocker has been proved to reduce glomerular inflammation, tubulointerstitial scarring, and restore renal function in experimental crescentic glomerulonephritis [81]. Our results suggested that excessive generation of pro-inflammatory cytokine TNF-α in ADR-induced NS mice, as indicated by elevated TNF-α level in plasma and protein expression in renal tissues. However, significant decreases of TNF-α level were detected in all GATE groups compared with model group. Moreover, Huang et al. [13] have reported aqueous extract of *G. tabacina* could inhibit NO production and expression of iNOS and cyclooxygenase-2 in LPS-activated RAW 264.7 macrophages. These results illuminated that anti-NS effect of GATE was partially via its anti-inflammatory role and down-regulation of pro-inflammatory cytokines in NS mice, which were consistent with the results predicted by network pharmacology.

Taken together, our findings illuminated that GATE exerted renoprotective effect possibly via the regulation of oxidative stress and inflammation. Furthermore, the systematic isolation of active compounds from *G. tabacina* and detailed pharmacological mechanisms of *G. tabacina* against NS will be investigated in our further research.

## Conclusion

In summary, in the present study, the protective effect and potential molecular mechanisms of GATE against NS were explored with the combination of network pharmacology and experimental verification. Network pharmacology analysis demonstrated that the mechanisms of GATE against NS were multi-component, multi-target, and multi-pathway. Moreover, TNF, IL-6, CXCL8, IL1β, NOS3, IL-4, IL-2, NOS2, CAT, SOD2 and several pathways, which were closely related to inflammation and oxidative stress, were thought to play a significant role in the mechanism of action of GATE against NS. Further experimental evidences revealed that the oral administration of GATE notably ameliorated the NS symptoms and renal injury possibly through inhibiting oxidative stress and inflammation in ADR-induced NS mouse model, which at least partially verified the predicted consequences of network pharmacology. These findings provide preliminary evidence for the development of potential novel renoprotective agent(s) for the prevention and treatment of nephrotic syndrome from *G. tabacina*.

## Supplementary information

**Additional file 1: Figure S1.** Biological process enrichment analysis for the overlapping targets of GATE on NS. **Figure S2.** Molecular function enrichment analysis for the overlapping targets of GATE on NS. **Figure S3.** Cell component enrichment analysis for the overlapping targets of GATE on NS. **Figure S4**. Full-length blots for Fig. 6f are presented and cropping lines are indicated in red color. **Table S1.** 1806 NS-related targets selected from Genecards, DisGeNET and OMIM databases. **Table S2.** 280 corresponding targets of GATE collected from PharmMapper, Stitch, SwissTargetPrediction and TCMSP database. **Table S3.** 92 overlapping targets of GATE against NS. **Table S4.** The data of GO (biological process) enrichment analysis of GATE candidate targets on NS treatment. **Table S5.** The data of GO (molecular function) enrichment analysis of GATE candidate targets on NS treatment. **Table S6.** The data of GO (cell component) enrichment analysis of GATE candidate targets on NS treatment. **Table S7.** The data of KEGG pathway enrichment analysis of GATE candidate targets on NS treatment. **Table S8.** Detailed information of the interactions among chemical components of GATE, NS, targets and pathways.

## Data Availability

The datasets used and/or analyzed during the current study are available from the corresponding author on reasonable request.

## Abbreviations

NS: Nephrotic syndrome
GATE: *Glycine tabacina* aqueous extract
ADR: Adriamycin
ELISA: Enzyme-linked immuno-sorbent assay
TCM: Traditional Chinese medicine
TCMSP: Traditional Chinese Medicine Systems Pharmacology
OMIM: Online Mendelian Inheritance in Man
PPI: Protein–protein interaction
GO: Gene Ontology
KEGG: Kyoto Encyclopedia of Genes and Genomes
CC: Cell component
BP: Biological process
MF: Molecular function
PAS: Periodic acid Schiff
ROS: Reactive oxygen species
MDA: Malonaldehyde
BUN: Blood urea nitrogen
SOD: Superoxide dismutase
TNF-α: Tumor necrosis factor-α
NO: Nitric oxide
iNOS: Inducible nitric oxide synthase
eNOS: Endothelial nitric oxide synthase
