# Supplementary material for: Exploring protective effect of Glycine tabacina aqueous extract against nephrotic syndrome by network pharmacology and experimental verification
Source: Chin Med. 2020 Aug 1;15:79. doi: 10.1186/s13020-020-00361-7 (PMC7395350; doi:10.1186/s13020-020-00361-7)
Supplement: Supplementary file 1 — Additional file 1: Figure S1. Biological process enrichment analysis for the overlapping targets of GATE on NS. Figure S2. Molecular function enrichment analysis for the overlapping targets of GATE on NS. Figure S3. Cell component enrichment analysis for the overlapping targets of GATE on NS. Figure S4. Full-length blots for Fig. 6f are presented and cropping lines are indicated in red color. Table S1. 1806 NS-related targets selected from Genecards, DisGeNET and OMIM databases. Table S2. 280 corresponding targets of GATE collected from PharmMapper, Stitch, SwissTargetPrediction and TCMSP database. Table S3. 92 overlapping targets of GATE against NS. Table S4. The data of GO (biological process) enrichment analysis of GATE candidate targets on NS treatment. Table S5. The data of GO (molecular function) enrichment analysis of GATE candidate targets on NS treatment. Table S6. The data of GO (cell component) enrichment analysis of GATE candidate targets on NS treatment. Table S7. The data of KEGG pathway enrichment analysis of GATE candidate targets on NS treatment. Table S8. Detailed information of the interactions among chemical components of GATE, NS, targets and pathways. [file 13020_2020_361_MOESM1_ESM.docx]

**Exploring protective effect of *Glycine tabacina* aqueous extract against nephrotic syndrome by network pharmacology and experimental verification**

Lihua Tan^1†^, Yanbei Tu^1†^, Kai Wang^1†^, Bing Han^1^, Hongquan Peng^2^, Chengwei He^1*^

**Affiliation**

^1^*State Key Laboratory of Quality Research in Chinese Medicine, Institute of Chinese Medical Sciences,* *University of Macau, Taipa, Macao SAR 999078, China*

^2^*Renal Division,* *Kiang Wu Hospital, Macao SAR 999078, China*

^†^These authors contributed equally to this work.

***Correspondence**

Chengwei He, State Key Laboratory of Quality Research in Chinese Medicine, Institute of Chinese Medical Sciences, University of Macau, Taipa, Macao SAR 999078, China. E-mail: [chengweihe@um.edu.mo](mailto:chengweihe@um.edu.mo)

**List of Contents**

**Figure S1** Biological process enrichment analysis for the overlapping targets of GATE on NS.

**Figure S2** Molecular function enrichment analysis for the overlapping targets of GATE on NS.

**Figure S3** Cell component enrichment analysis for the overlapping targets of GATE on NS.

**Figure S4** Full-length blots for Figure 6F are presented and cropping lines are indicated in red color.

**Table S1** 1806 NS-related targets selected from Genecards, DisGeNET and OMIM databases.

**Table S2**  280 corresponding targets of GATE collected from PharmMapper, Stitch, SwissTargetPrediction and TCMSP database.

**Table S3** 92 overlapping targets of GATE against NS.

**Table S4** The data of GO (biological process) enrichment analysis of GATE candidate targets on NS treatment.

**Table S5** The data of GO (molecular function) enrichment analysis of GATE candidate targets on NS treatment.

**Table S6** The data of GO (cell component) enrichment analysis of GATE candidate targets on NS treatment.

**Table S7** The data of KEGG pathway enrichment analysis of GATE candidate targets on NS treatment.

**Table S8** Detailed information of the interactions among chemical components of GATE, NS, targets and pathways.

**
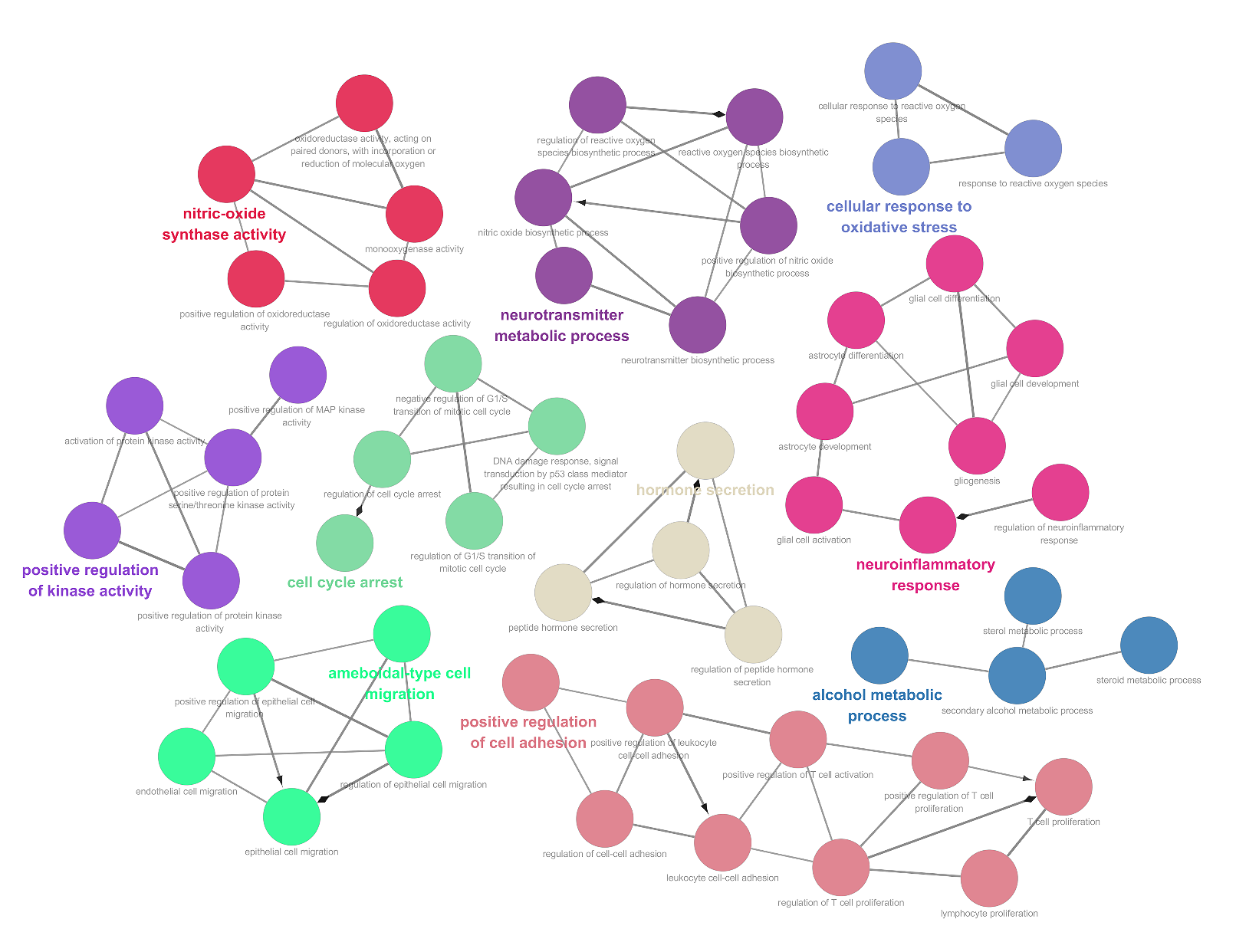
**

**Figure S1.** Biological process enrichment analysis for the overlapping targets of GATE on NS. GO terms are interconnected and functionally grouped based on the Kappa score. Nodes in the network indicate GO terms, and size of the nodes stand for the significance of GO terms after Benjamini-Hochberg correction. The most significant GO term of each group has been highlighted.

**
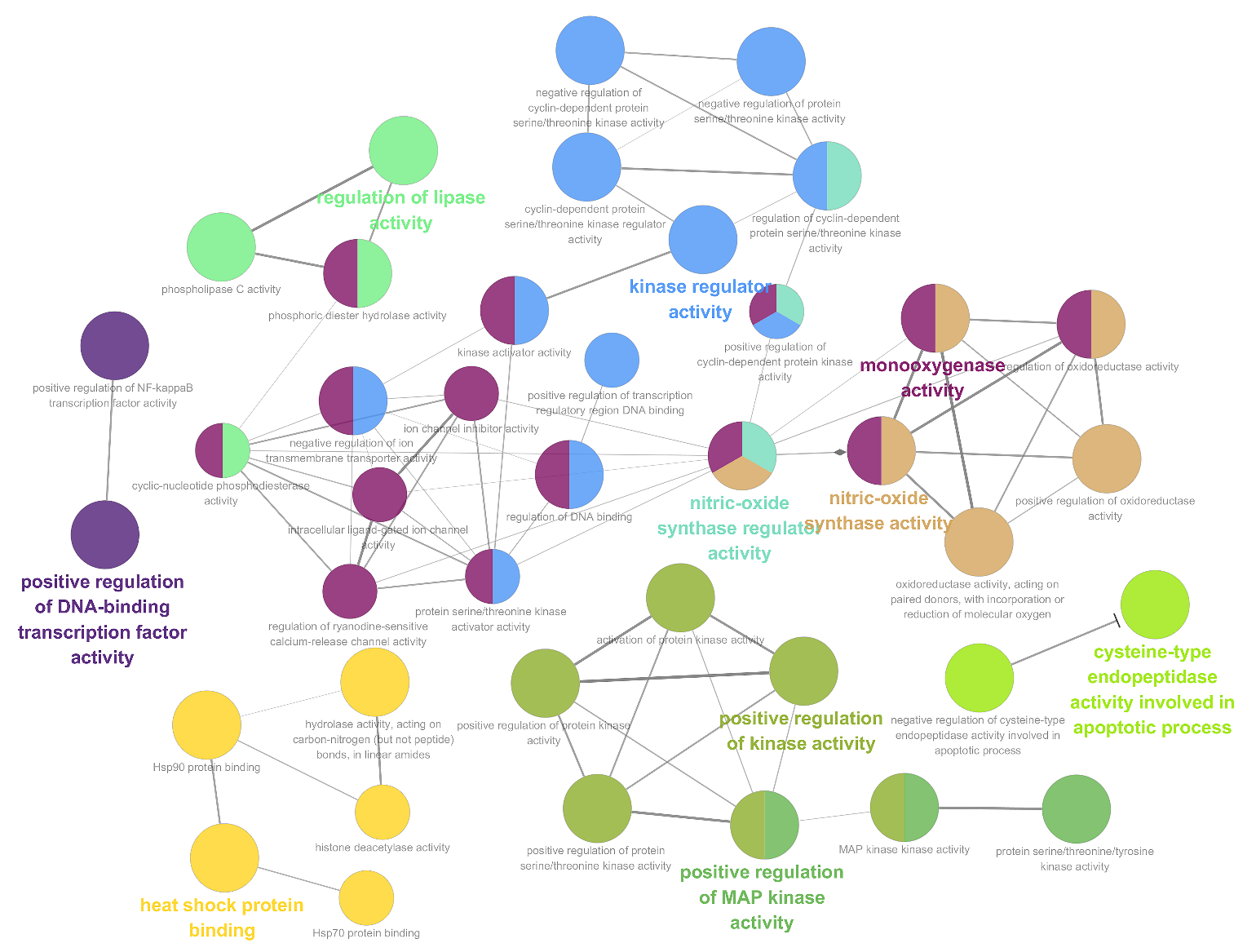
**

**Figure S2.** Molecular function enrichment analysis for the overlapping targets of GATE on NS. GO terms are interconnected and functionally grouped based on the Kappa score. Nodes in the network indicate GO terms, and size of the nodes stand for the significance of GO terms after Benjamini-Hochberg correction. The most significant GO term of each group has been highlighted.

**
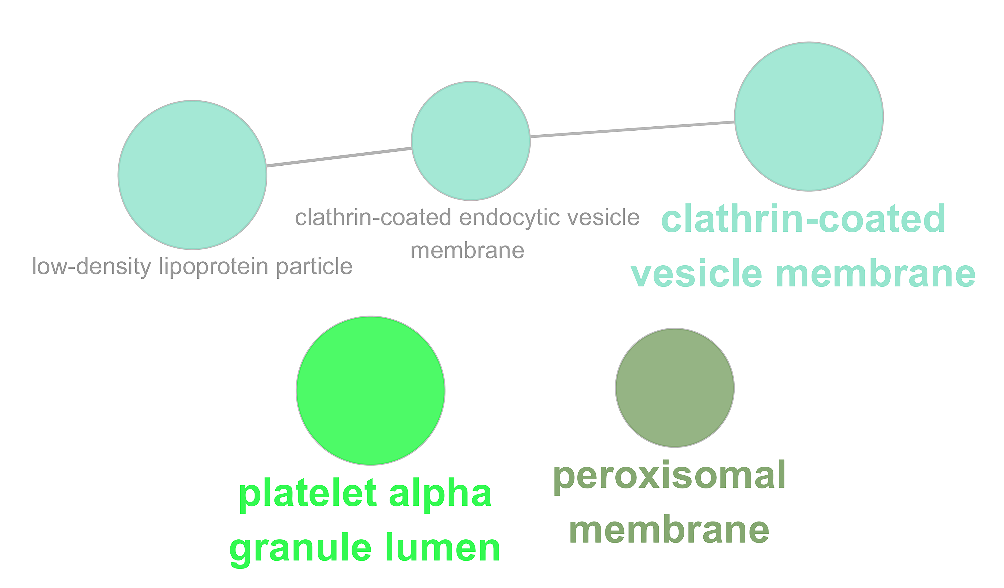
**

**Figure S3.** Cell component enrichment analysis for the overlapping targets of GATE on NS. GO terms are interconnected and functionally grouped based on the Kappa score. Nodes in the network indicate GO terms, and size of the nodes stand for the significance of GO terms after Benjamini-Hochberg correction. The most significant GO term of each group has been highlighted.

TNF-α 17-26 kDa

**
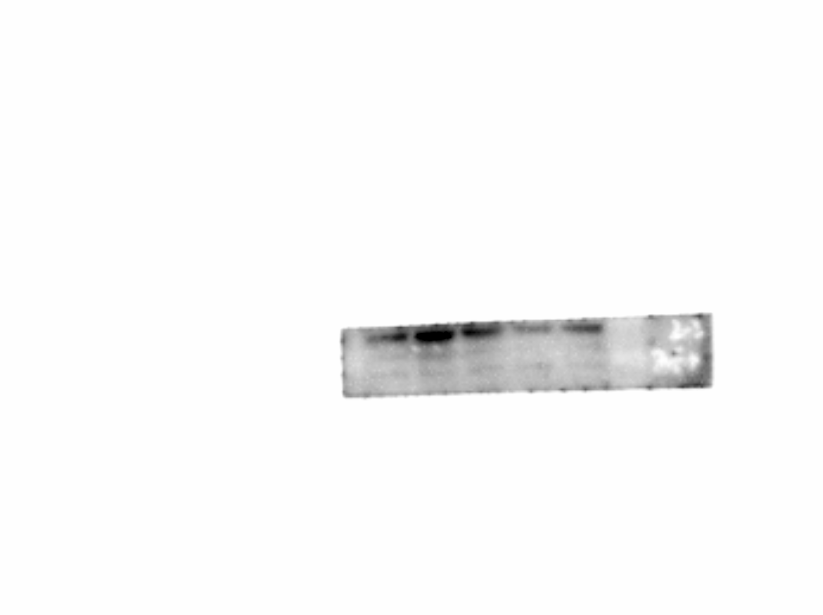

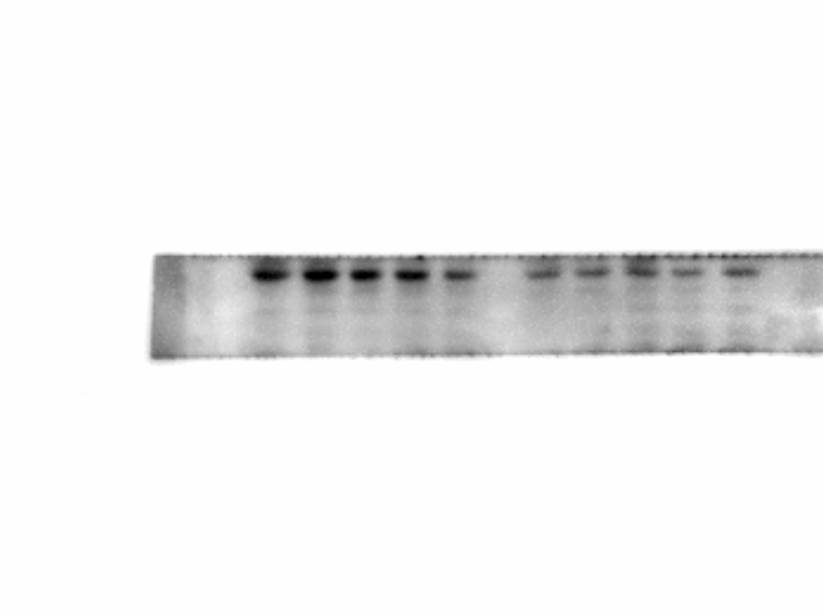
**

**
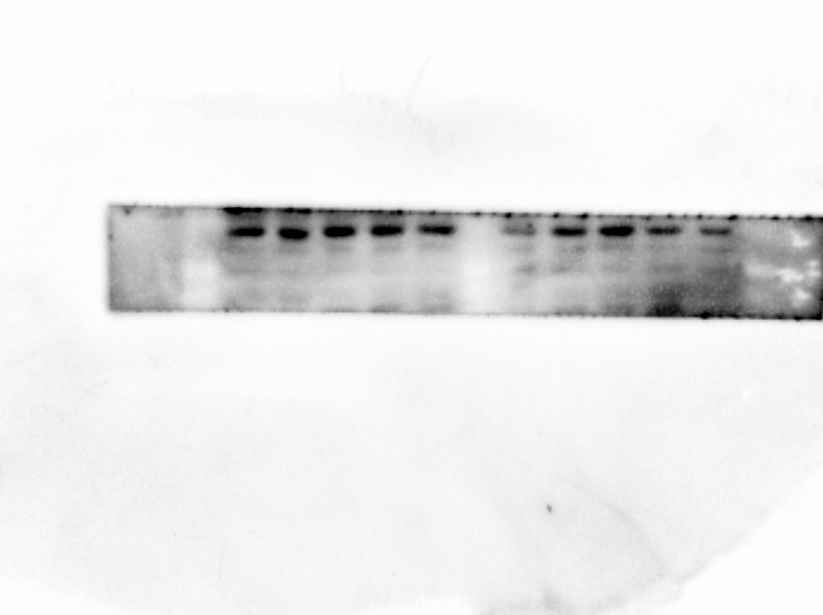
**

GAPDH 37 kDa

**
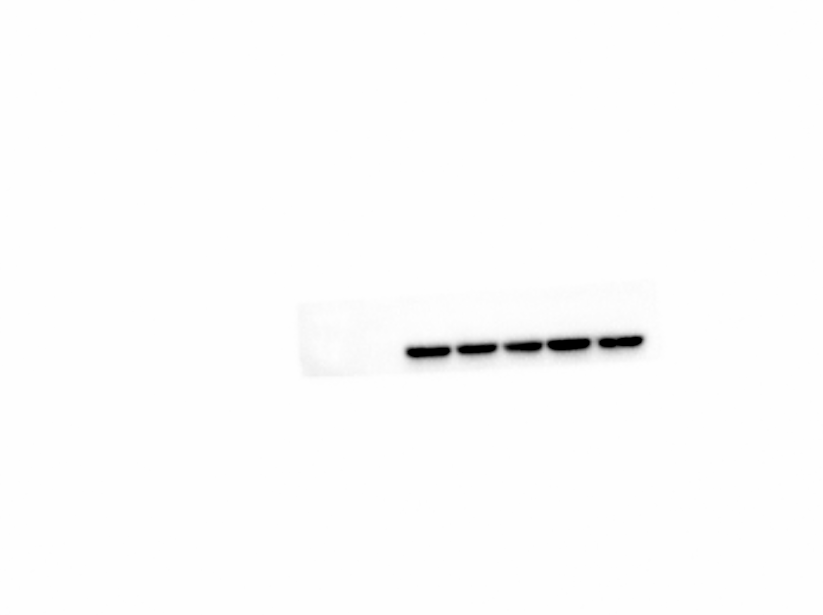

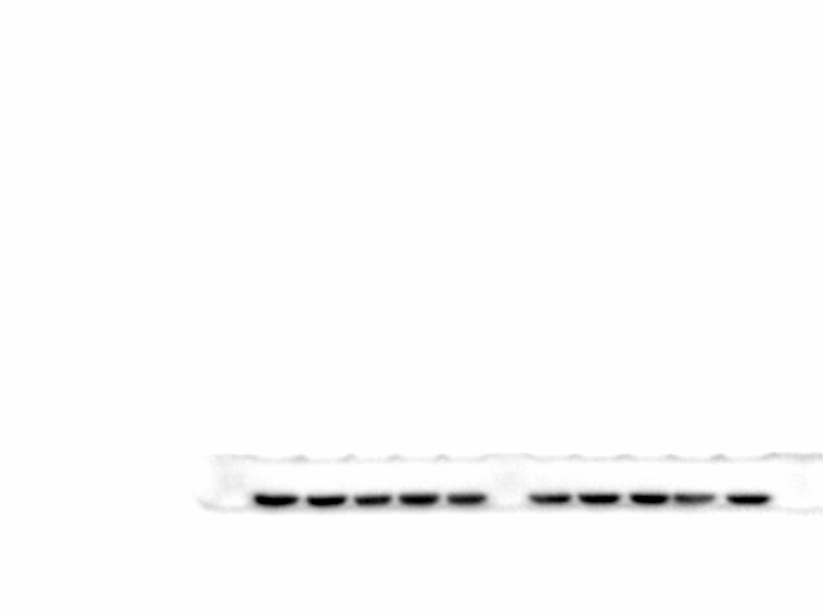

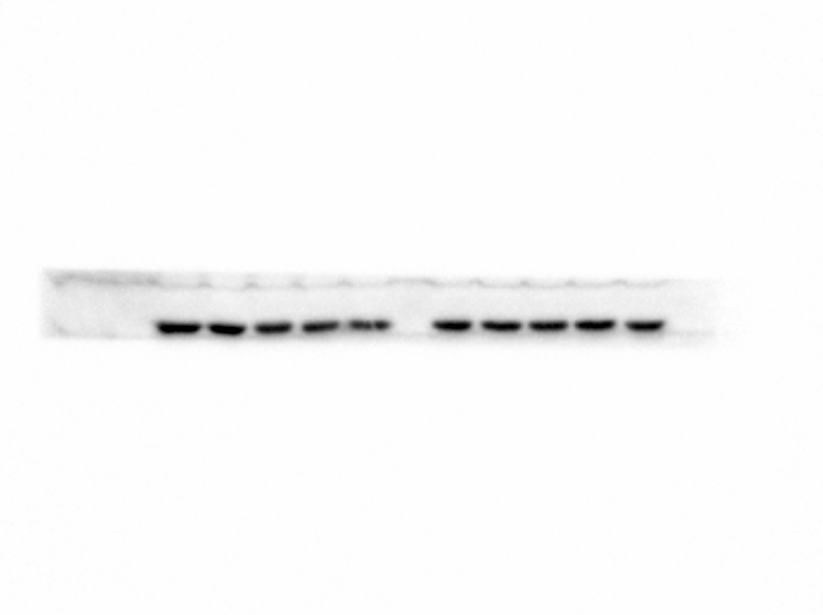
**

**Figure S4** Full-length blots for Figure 6F are presented and cropping lines are indicated in red color. From left to right are Control, Model, Low-GATE, High-GATE and Benazepril groups. The unmarked blots had no connection with this assay.

**Table S1** 1806 NS-related targets selected from Genecards, DisGeNET and OMIM databases.

| CLMP ACTN4 HPSE2 CD44 MMP2 DONSON PLCE1 TMEM216 SLC30A9 DLG1 KCNJ2 MIR4677 VPS33A ERCC5 CNTNAP1 LPP PSTPIP1 MEFV FIBP NBAS ERAL1 ATRX EDN1 CHST8 ABCB7 HSPB1 CYP11A1 INPP5E NBN AHDC1 CXCR4 SALL1 CUBN WASL RMRP IRAK1 AIFM1 SEC24D CFHR5 POLR2L SOS1 PLAU GAS5 C16DELq22 KMT2E CHRNE RAC1 FRAS1 DDB1 MYMK C1R LZTR1 ATP1A3 B9D1 NKX2-5 WS2B DEL16p12.1p11.2 MITF POLR1C GPSM2 NID1 SETD2 DEL1q21 KIRREL1 ROGDI KIF7 DEL10q26 CXDUPq26.3 DDX11 DTNBP1 SEC23B COL4A5 RFX5 SMARCC2 HMGB3 FAT4 NLRC4 MDLS PADI3 MIR15A PKP1 JBS C17orf67 CCDC28B GNAI3 KTWS COX15 KCNE1 XK POLG2 CD4 DSP IRF1 NSMCE2 SPECC1L CLCNKA TTC37 FAM20A SDCCAG8 CLRN1 SOX4 HOXA1 MBL2 CECR DSCR8 LONP1 SH2D1A MUSK EVC FRTS1 HOXA13 FWS ALOX5 NR2F1 CHD1 MRXS17 APMR1 NLRP3 LEP GJA1 MRXSAB PKD1 FIT2 DDOST PUF60 HMX1 CHSY1 DEL16p13.2 DEL9p DEL8q21.11 IRX5 RAB3GAP1 NSD2 HSD11B2 ITGA3 EXT2 DUP16p13.3 MECP2 NOS1 SMO MSL3 BMPR1A A2M PRL COL9A2 TEMPS CIB2 LMNB2 STAG2 DEL17q11.2 GPC5 BBS12 MT-ATP6 TCHH LARP7 LVSKS COG6 DUP2q31.1 SQSTM1 SNORD3A DSCAML1 EED SMC3 RAC2 COL1A1 LARS1 HPS6 DGUOK AP1S2 DEL17q23.1q23.2 MTHFR ACAT1 MEOX1 CECR2 SIX5 SLC18A3 DSPS INTU PTPN11 SMAD3 IPW RIEG2 CMIP NUP93 KDM5C KCNA1 DUP22q13 PDCD4 ANLN TBX5 FOXP3 DEL13q14 SELL GNAS PLCG1 NPHP1 COQ7 DDB2 RBM10 SYT2 MDH1 ITGB4 GDF6 DICER1 FOXE1 APOC3 BBS7 C2CD3 AMMEC CD38 GLE1 MIR19A IKZF1 RIPK4 GTF2H2 GGA3 IFNA2 RBPJ ROR2 ANGPTL3 ALK CASP10 SLC25A1 EZH2 DOCK8 APOE PF4 VAMP1 MSH6 MUC16 ZFP37 EVC2 NR2E3 AIRE RFXAP SGPL1 SCZD16 FAS C3 EMP2 ALG2 DEL4q21 KATNIP PKDTS PON1 USH1H PI4KA CDKN1B PRBNS FOXC1 DMRT3 COQ9 DSCR6 DUP7q11.23 ABCA1 SEMA3E PQBP1 RLS3 SMARCB1 PGAP2 DEL8q13 MYH6 ARL13B ORC1 TNFRSF11B MIR27A GMPPA IFT140 SON CD36 PIBF1 MCOPS8 PDE6D COL18A1 NUP37 MYH9 TNXB A4GALT HSD17B4 IDH2 SPRTN WBSCR26 TF CLCN4 HLA-DRA SOS2 DNMT3B MYBPC1 ASS1 ICR1 OTDD DEL3q29 WDR19 RSPH4A EDNRA NIPBL PKS PIGV STIM1 KCNH2 CEP120 POLG POGZ TMEM237 TFR2 ALG14 RLS2 F9 RAG1 B4GALT1 CHD3 SRP72 RPS27A APMR3 WT1-AS ATR FKBP14 STAT4 TNNI3 CD40 MRXSCS NR0B1 RYR1 LAGE3 KCNE3 PPBP BACE2 ACTC1 CASP8 TAP1 CFH CYP11B2 TSC2 USP19 EPX DCR SCN4A KDM6A WNT3 EZR NCAM1 MYMY4 OFD1 FGFR3 IL5 AGT CGA KRAS MLSM7 ATP7A SPTAN1 BBS1 EMG1 C4A CDC6 CST3 AQP4 COL7A1 BBS10 COX10 TFAP2B JAG1 ACF GCA IRF6 CIITA HPS4 SERPINB7 CRP OCRL GLA SMC1A RARB CALCA PRKCD QRICH1 ZBTB20 NEU1 SERPINA1 ANTXR2 COL4A3 MGS DUPXq25 ADAMTS2 PEX1 DPF2 ASPG4 NPHS1 TAPBP RET NAGA LZTFL1 MAPT COLEC11 KLHL7 HOXD13 GSTP1 XPC AOMS2 TCF4 MID1 C8orf37 MED12 NAALADL2 HLA-DQB1 FXN DEL6pter NRAS PNPLA6 AP3B1 ZDHHC24 NOC3L AHI1 KIAA0586 GRHL2 AFP KIF14 SCN11A FHL1 C12orf29 GATA1 OSGEP PBX1 ABCB1 PHOX2B ALPP RSPO2 ATPLS TTC21B LOXL1 PWAR5 USP9X FTL PACD SLC34A1 TGM5 SPINK5 MIR181A2 THBD NR3C1 NR5A1 PIK3C2A CHKB BRF1 HUWE1 SCN1A HP OPA1 CDH17 RPL10 POLA1 IL17A PIGL ASPH IDS COG2 YY1 CSTA RRAS2 LEPR LTBP2 MTOR BMP6 DEL2p21 CDSN NPPA KCNJ6 PPARGC1A SLC19A3 CCCSX ASXL2 WT1 ILK CD151 KCNQ1OT1 EYA1 PITX2 PLA2G6 CYP3A5 NPTX2 SOX9 ZFPM2 CCN2 PIEZO2 CUL4B CENPJ SATB2 WHS PITX1 PDGFRB EIF2S3 KLLN SNAP25 CHRNB1 TH EEC1 RSCIS DCTN1 TMTC3 COG4 GRIP1 AFF4 C1QBP INF2 CRB2 LYST KLRK1 FURIN IKBKG GSN NSD1 AMMECR1 GLDN EDAR BOS2 SLC2A10 DEL11p15p14 PCOS1 IL1A GNRH1 ERCC1 MYH3 CAV3 EDN3 YY1AP1 KCND3 BLM ANK2 RAB23 KCNE5 CREBBP ASTN2 FKBP10 VLDLR PAX1 MAP2K2 ABCC2 COL5A2 AVIL INVS HPS1 MPO TECT1 MIR32 ELN SELP CSPP1 CALR VIM RJBS NCAPG2 OFD8 DES HPS3 FGFR2 SOX10 SAMD9L PCNA CSF3 HBHR HLA-A LOC105378438 C1GALT1C1 EDNRB DUPXp11.22 CD27 DSE NUP85 SCN4B JAK2 SRS MIR106B ANTXR1 CHUK CHAT HS6ST2 DGAT1 TNFRSF1A KYNU CASP1 DCAF1 CD8A DEL3q13.31 INDX CD2AP MED25 SPP1 GLI1 MIR17HG DGCR5 PHGDH CTBP1 WSHC5 LOR WNT5A APOA2 TRPA1 VAX1 AGK ATP6V0A2 ANKRD11 MT-ND4 SLC29A3 EBF3 ITPR1 TBC1D20 USH2A B2M CFHR1 NF1 LRP5 SPRED1 MYO5A CNNM4 MEGF8 CD2 SPG7 PRPS1 NUP133 CTNNB1 SLC26A4 MT-CO3 CHST14 KIAA0753 H19 GAMT SNTA1 HNRNPK TGFBR1 CLIP2 DUP14q32 KANSL1 DNMT3A WDR4 FSHR VWF NAA10 VTN LRPPRC TBX1 IL18 NCK2 SLC16A2 CCND1 IGFBP2 CRLF1 FLNB NOTCH2 MT-TW USP18 SMCHD1 PSMD12 WAS MGP CLDN10 PCDH15 TRAIP KCNN3 BLOC1S3 BCS1L ERCC8 LIG4 SIN3A BMP4 AXDND1 ACE2 DEL6q24q25 WBSCR17 MCAM AGTR1 DVL1 EDA2R CDCA7 DNA2 RNF168 MAB21L2 ASPG2 SCNN1B GRHL3 MCM5 ALG9 CISD2 ANAPC1 DEL22q11.2 SRRT PCGF2 DEL15q25 APTX SLC52A3 IBA57 RAPSN SNCA ITK CSNK1D TJP1 ZFHX2 KCNJ10 FASTKD2 JAGN1 CD46 PER2 ICOSLG RNF43 RNU4ATAC ITGB2 SCA20 XYLT2 CTU2 HJV SMAD4 TXNL4A DHCR7 PDZD7 PSMB8 POR DELYq11 LAMA1 APOL1 SETBP1 PER3 CDH11 PIK3CD PGBD3 IL3 DEL19q13.11p TTC7A GLI3 TWNK DEL18q PTCH1 ERCC3 APEX1 SHANK3 DELXp11.3 PAX2 TFRC SCARF2 TINF2 SLC5A3 DEL17q12 DUP16p11.2 PRKAG2 MYC WDR26 NRXN1 BCOR AVPR2 NLGN3 NUMBL PLCE1-AS1 SERPINF2 RLS7 FBN1 C12orf57 WWOX PLOD3 RLS6 ARID1B ABCA12 PLOD2 KONDS ISCA1 HARS1 DFNA51 LEMD3 PEX6 B3GLCT LOC105371841 WDPCP NIPA2 NKX2-1 LAMA3 BGN SLC35A1 MAP3K1 ARID2 IL13 RDH11 DUPXq27.3q28 MT-ND1 IQCB1 LAMA5 PIP5K1C SKIV2L DIS3L2 GPD1L FREM2 TNFAIP3 PIGT TBX15 ADAMTS17 MT-TS1 GPT CNKSR2 CLCNKB WAGRO ATAD3A MIR125A PRNP NPHP3 TRIP13 MMP1 CEP104 B9D2 DEL2q31 ADA MAGI2-AS3 DUPXq28 HLA-DQA1 EIF2AK3 COL13A1 LRSL DUP2q35 KCNK4 SSNA1 ANXA5 TK2 DLL4 GTPBP2 CCDC8 NMLFS SLC2A1 NSDHL DUP17q21.31 MKKS KCNQ1 SMARCA1 MAST1 MRXSA EPOR ZBTB24 RHO BANF1 CCDC47 EBP SLC25A24 SLC25A15 TGDS RAB39B DEL1q41q42 TIMM8A FOXG1 TCTN3 DYRK1B WFS1 MIR335 DOCK6 ENTPD5 ADGRG6 SYP PARP1 KIAA1109 SUCLG1 PTPRC TMEM67 MYO1E MDNS KCNH1 SNAP29 VDR ACE CDH3 IFT74 AK1 TBC1D24 CD34 AAAS L1CAM NR3C2 TGM1 FAM111A C4B DGCR8 DEL2p12p11.2 WDR81 CACNA1C DEL18p COL3A1 FIG4 FASLG ZBTB42 GAPVD1 ARHGAP31 UBR1 COL9A1 GGT1 LCS1 DUP17q12 GPC4 CCL11 ZC4H2 COL4A2 TBC1D4 SRCAP KRT14 GLUD1 SYT1 OBSL1 BBS2 AIC C1S IL1RAPL2 MRM2 FCGR2A ASXL3 PHIP NECTIN1 FOXP2 NBEAL2 SERPINB8 GTS NLGN4X POLR3A PCDH12 COQ6 EXT1 HOTAIR NPHP4 PECAM1 VPS26C ABHD5 TAF6 GUCA2B TNC CPLANE1 ACRPV NXN ADA2 KMT2D MMP3 BGLAP CC2D2A MRAS TBX4 SACS AXL TELO2 RINT1 TRPS2 DVL3 GATA3 DUP3q29 TFAM IL2RA ATP6V1B2 ALG10B PLCG2 FMR1 TKCR KCNJ1 ARX PRG4 TTR BBS9 CCR1 CLDN5 SLC12A3 GTF2H1 SHFLD3 MPZ FLCN PSAP MYOM2 SLC5A7 ERCC4 ATP1A2 FOXC2 SHFM3 IFT172 ASPG1 TGFB3 POMP GDNF CTNNA1 CTNS RNASEH2C RBP4 MYO9A DEL16p11.2 IL10 AGRN DHH CEP290 TBCE PLCB4 RAI1 MT-TF LEPQTL1 LAMB1 MYRF IGF2 MCOPS4 SERPINA7 CHD7 CEP63 CD80 CHRNA1 GSX2 PHF6 GFND1 DEL16p13.3 EFL1 NHLRC2 DER22t8-22 SOX5 GP1BB OTX2 DSCAM DEL15q15.3 WS2C NFIX CFI DUP17p13.3 SH3PXD2B SAMHD1 OCLN ACRPS ATP13A2 MBS DMXL2 NAGLU MYSM1 MMP14 UBE3B PLAT EDSS2 ANOS1 KCNK9 BCORL1 DEL11p13 PIGA LPA COL4A4 NDUFAF2 TAP2 TNFSF11 TPP1 RPGRIP1L AKT2 MT-TS2 BRAT1 SLC25A4 LIFR CBL HSPA9 AXIN2 GP9 SURF1 MBCS MYLK SOD1 TAF1 PACS1 CD79A ZDHHC9 CLCN5 RLS1 MAGI2 PIK3CA USH1E COLEC10 SLC9A6 CTLA4 MIRLET7B HTT NUP160 DEL3pterp25 FGF9 ERBB3 APOH MFF-DT CREB1 NIN MRXSMP FGA SMAD1 FLG2 IGFBP1 NPHS2 GUST SPG21 RAB3GAP2 CDH1 PSAT1 CCNQ ANKFY1 IFNG IQGAP1 NECTIN4 EDA PSS LPIN2 PDGFA RETN CDT1 TNFSF13B HELLPAR SLC12A1 CFL1 CD28 SMARCA4 IGES WNT7A ARMC9 FIP1L1 MASP2 TTC8 GNAQ BBS4 XPA TENM3 FGS5 MGME1 KIRREL2 FGF2 AMH CLU STAT5B KIFBP CXCL12 MIR93 SLC39A13 SKI EPG5 TET2 SLC35A2 COL2A1 GABRD MSX1 WNT10A DIAPH1 TBL1XR1 G6PD AKAP9 MT-ND3 DEL17p13.1 DLX3 SIL1 TPK1 SLC27A4 FEN1 RBBP8 PVALB GJB2 PGAP3 DEL19p13.13 SF3B1 SCN1B RNASEH2B STAR F2R RFC1 BCL2L1 CD19 HELLS TNFRSF1B TMEM260 ABL1 PIK3R2 SCARB2 PIGN AEBP1 CRBN LOC107986083 VSPA MT-TQ CALCR SLC6A8 SNRPN CDC45 TBX3 DNAJC21 SLC9A1 TAZ CHRND SRD5A3 COQ8A CTSL ASPG3 ZNF423 PSMB4 HLA-G PPP1CB IL6ST LRP2 ARSG FGD1 RLS4 RAD50 HTGH SYNE1 CFSS MYCN POLR1D MBTPS2 TBX18 MVK TSPYL1 DUP1q21 UVSSA ABCB4 TMEM138 TRPC6 MT-TH MCP PIGY GUSB PMPCB HYLS1 CRELD1 DNM2 EHMT1 HNF4A CNTNAP2 MYH7 USH1G BZX PLA2G7 DELXp21 TMCO1 CHD4 TMEM231 CEP19 SHFM1 TRIM32 AP3D1 STRA6 HTRA1 ADCY6 MRXS12 SLC25A19 DDC PRS PTLS GDF5 DEL19q13.11d CDC73 B3GALT6 CETP APMR2 VSX1 APOC2 MT-ND5 WBSCR27 ADAR DUP17q23.1q23.2 MPV17 SERAC1 WRN LAS1L CACNB2 MIR25 GPDS1 CD70 MSH2 KMT2A SAA1 DCPS RIT1 ZEB2 CYLD SHOC2 SRC CASR GPAM CANT1 DEL2p16.1-p15 DUPXp11.23p11.22 TFAP2A BUB1B MIR16-1 BMIQ16 NUP107 ERF MT-CO1 ARL3 FGF10 IL1RN ERCC6 THOC6 DGCR6 MAFB SALL4 BAZ1A RFX6 PHF8 PTPRO CDKN1C POLE BBS5 COL17A1 TUKLS IFT27 NIPA1 COL4A6 IL7R ACTB TWIST2 ESCO2 TLR4 DDX59 B4GALT7 BSCL2 BDMR NSMCE3 SBDS DEL1p36 CEP41 MAN1B1 ADAMTS13 DEL16p12.1 PLG RLS5 ATP6AP2 TYR RNASEH2A CKAP2L HFE COL4A1 SERPINE1 SUFU VCAN SPATA5 UBE2A ADAMTS3 THAS DSCR4 TBC1D7 PANK2 MYO7A NHS STS CD96 MDS1 MIR17 IL4R KANK2 GLI2 DGCR6L SHROOM4 DEL15q26qter EDARADD ALG3 IFNGR1 MAGEL2 COQ8B MIR140 STK11 RBMX AOMS1 SAA4 MYH8 INSR TGFBR2 CDH23 TTN DWS PRDM5 MIR184 MT-TK SCLL FLG LAMB2 IFNA1 HNF1B DEL14q11q22 SLITRK1 HAAO UMOD HLA-DRB1 MAP3K7 MEG3 EHD1 DEL15q14 C19orf61 RAD51D IER3IP1 FERMT1 DYRK1A ZNF469 NCK1 LPL LAMP2 DGKE NOG HLA-B HCN4 DURS1 MARS1 CUL7 LIPC WBSCR28 CTRL KIRREL3 DELXq21 TCTN2 YUHAL CTNND1 MT-CO2 NONO ARHGDIA F5 LYZ RBM8A VANGL1 ACSL6 TCPT SCNN1A PIGO KCNE2 GATA2 SLC2A2 RCHTS CCND2 DDX58 CFB ZNF462 PIK3R1 GPC3 MAF PDGFRA RNF125 ADM STAMBP RPGR RRM2B PREPL FGS3 CCR5 ZNHIT3 HIGD2B OTULIN GMNN TRPV3 IFIH1 ASXL1 HADHA IDH1 WDR37 CCBE1 RAB27A PITX3 PAX3 MRXS9 AHSG YAP1 SMARCAD1 MIR342 MALAT1 LMX1B LRP4 RAD21 SNRPB HMPS1 MASP1 MIF MLXIPL SHOX MAP2K1 ORC6 KAOGS HPS5 BOLA3 SOX11 SIX1 ITGB3 CDAGS CEP152 CFHR3 NUP205 CHRM3 NEK9 TBX22 DCHS1 HARS2 DEL5q12 TPRKB CCDC22 UPF3B COQ4 SMARCA2 DEL6q11q14 WIPF1 ADNP ATP8A2 POLD1 ADAMTS10 BSND LCAT PAND1 POMC BLOC1S6 WNT4 BCL11A SLSN3 TMEM107 DUP8q22.1 UBE3A RLIM SAMD9 NLRP12 KITLG LCN2 VWSM ST3GAL5 ORC4 DDR2 NFKBIA IGFBP3 COL5A1 ACTA2 TRAPPC3 ARHGAP24 ALB FREM1 IFT57 C1QA ALMS1 SAA2 ZNF592 HAMP ALDOB SERPINC1 DCN TRIP4q32.1q32.2 F3 NOD2 CFP ARSL COL11A1 USH1K SCNN1G SYNPO MLH1 RAB18 KCNJ5 LMNA HNRNPH2 VAMP7 PDSS2 CLIC2 BBIP1 SCN10A MKS1 DEL8q12q21 POU3F3 LARS2 LTA ASCL1 PMP22 PSEN1 APC TLR2 RC14S MDS2 GALNS CHN1 FAM20C RAG2 ADGRV1 PMM2 RECQL4 DPAGT1 DUP22q11.2 MAGED2 BCL10 TRPS1 ZNF341 SERPINA3 CCL3 DOK7 DEL9p24.3 TAM EOGT NOTCH3 PTH1R MLPH DEL15q13.3 FBXL4 TREX1 TGFBI FRIASS ESPN CLPP CEP57 MCOR ACTG1 NFU1 CHRNG SNAI2 ALG1 DGCR2 DEL7q11.23 COL27A1 GATM MYO18B PLAUR WBS CDKN3 MESP2 CLCF1 SPINT2 CASK CSNK2A1 HDC HSPG2 IQCC FLNA TWIST1 WAC RBM28 EPO WHRN DER22t11-22 NOTCH1 COL1A2 SLC52A2 CP IGHE G6PC3 AKT3 SUCLA2 IL1R1 TP53RK AMHR2 ANO6 SMARCAL1 ERCC6L2 MAB21L1 TBC1D8B TCOF1 GSTT1 EHHADH KMT2C CD40LG SLC17A5 COQ2 HGF TRAF3IP1 USH1C SMARCE1 SOX18 ATP8B1 TYMP CELA2A PYCARD LRIG2 ARID1A MRXS7 ACSL4 WDR73 IL7 PRUNE1 PPM1D SCN9A SCN5A KAT6B SOX2 FLVCR2 IGHR AP1S1 TP63 PAK2 SDHA H1-4 DEL10q23 GATA4 VHL MNX1 LMBR1 ADFN PDLIM5 CENPF RPS6KA3 DACT1 CDK10 FGF23 MT-TL1 COLQ APC2 PTCH2 ERCC2 SLC19A2 C1DELp35 VPS13B MROS THM DCLRE1C ISCA2 SPART NDN DEL15q11.2 PSMB9 CCDC88A ARL6 DUP5p13 MEF2C CSF2 SMCR8 ARNT2 KCTD1 GDF3 LBR SOAT2 SCN3B POLR3B IL2RG SRY DARS2 RLS8 PLXNA3 CTSC PLOD1 ADIPOQ ZAP70 IGF1 TGFB2 GP1BA PTEN EP300 NOS2 SOD2 FGFR1 UGT1A1 ELANE ALDH3A2 MAOA APOB MTTP CXCL8 SELE FN1 ECE1 CYP19A1 MAPK14 TNF RAF1 LDLR KIT STAT3 F2 XIAP TBXAS1 PTGS2 TGM3 CDC42 CDK4 HRAS PDE3A ADRB2 VEGFA TGFB1 MME CCNA2 TIMP1 MT-ND6 CALM2 ACHE CCL2 SHMT1 IL6 HSP90AA1 CASP3 HMGCR KDR INS GSTM1 MDM2 EGFR IL2 FOS IL4 IGF1R HDAC8 ICAM1 HPRT1 CHEK2 HIF1A NOS3 CFTR BAP1 CCL5 TP53 CDKN1A CAT AKT1 PRKACA GH1 IL1B REN P4HB HSD11B1 DNTT APOA1 CDKN2A JUN DHODH CALM1 BRAF BAX KLK3 CYP3A4 APP HDAC6 PMS2 MMP9 |
| --- |

**Table S2**  280 corresponding targets of GATE collected from PharmMapper, Stitch, SwissTargetPrediction and TCMSP database.

| ZAP70 IGF1 TGFB2 GP1BA PTEN EP300 NOS2 SOD2 FGFR1 UGT1A1 ELANE ALDH3A2 MAOA APOB MTTP CXCL8 SELE FN1 ECE1 CYP19A1 MAPK14 TNF RAF1 LDLR KIT STAT3 F2 XIAP TBXAS1 PTGS2 TGM3 CDC42 CDK4 HRAS PDE3A ADRB2 VEGFA TGFB1 MME CCNA2 TIMP1 MT-ND6 CALM2 ACHE CCL2 SHMT1 IL6 HSP90AA1 CASP3 HMGCR KDR INS GSTM1 MDM2 EGFR IL2 FOS IL4 IGF1R HDAC8 ICAM1 HPRT1 CHEK2 HIF1A NOS3 CFTR BAP1 CCL5 TP53 CDKN1A CAT AKT1 PRKACA GH1 IL1B REN P4HB HSD11B1 DNTT APOA1 CDKN2A JUN DHODH CALM1 BRAF BAX KLK3 CYP3A4 APP HDAC6 PMS2 MMP9 HSD17B1 EIF6 GSTA1 ERI1 MMP13 DCK CCNB1 GALK1 B3GAT1 PCOLCE MAPKAPK2 HNMT RXRA NT5M BIRC5 PLK1 HTR2C GHR DPP4 GSTA2 TPI1 ALDH2 MAPK12 BDNF CDK1 PNMT GNPDA1 CYP21A2 SHBG FGG CRYAB CHEK1 BCHE BCL2 RHEB GCK GPI LARS F7 ADORA2A ESRRA CYP1A1 CDC25C PPARG RHOA AHCY ESRRG BST1 PTGFR AHR PIM1 F11 BTK GABBR1 CDK6 UMPS WARS ITPKA AMY2A HSD3B1 BARD1 TNK2 PCK1 HPGDS CYP2C9 HK1 HEXB TRPM2 CLEC4M ATM ESR1 PDE4D ESRRB CDC20 ANG ACPP HPGD FKBP1B CHIT1 BUB1 PBK GSK3B B4GALT4 CA12 STAT1 LSS EIF4G1 CA7 CLK1 LGALS2 MGAM SULT1E1 ABCG2 MDC1 EIF4E PTPN1 PML PPARA AMY1A PLK2 PTGER2 NCS1 MMP8 AHSA1 TEP1 MAP2K5 CA4 MAPK1 PYGL GADD45A RBM45 RNASE3 PIK3CG CD5L SP1 SIRT5 ADORA1 ERBB2 SYK ALDH3A1 LGALS3 TRDMT1 TPSB2 DTYMK CCNB1IP1 HSD3B2 FKBP3 MAPK8 F10 CAV1 MAPK3 NCOR1 NCOA1 GSTA3 RELA DUT CDK2 CDC37 NR1H4 GFAP CALM3 CD209 ARG2 MKI67 VCAM1 MSLN CASP9 RAD51 RAB11A ATP5F1B GSTZ1 ADAM17 GCG FCER2 IMPA1 FABP4 SRM GSTA5 TGFB1I1 CPT1A PTGS1 HTR2A FOXO3 AKR1A1 KCNJ11 RASGRF2 GLB1 GSTM2 UAP1 SULT2B1 MT2A CDA TFF1 PPARGC1B AR HINT1 ESR2 CYP24A1 ARL5A CFD KAT2B PRSS1 PAH SAFB UCK2 CBR1 OAT |
| --- |

**Table S3** 92 overlapping targets of GATE against NS.

| **No.** | **Target** | **Symbol** | **No.** | **Target** | **Symbol** |
| --- | --- | --- | --- | --- | --- |
| 1 | Tyrosine-protein kinase ZAP-70 | ZAP70 | 47 | Interleukin-6 | IL6 |
| 2 | Insulin-like growth factor I | IGF1 | 48 | Heat shock protein HSP 90-alpha | HSP90AA1 |
| 3 | Transforming growth factor beta-2 proprotein | TGFB2 | 49 | Caspase-3 | CASP3 |
| 4 | Platelet glycoprotein Ib alpha chain | GP1BA | 50 | 3-hydroxy-3-methylglutaryl-coenzyme A reductase | HMGCR |
| 5 | Phosphatidylinositol 3,4,5-trisphosphate 3-phosphatase and dual-specificity protein phosphatase PTEN | PTEN | 51 | Vascular endothelial growth factor receptor 2 | KDR |
| 6 | Histone acetyltransferase p300 | EP300 | 52 | Insulin | INS |
| 7 | Nitric oxide synthase, inducible | NOS2 | 53 | Glutathione S-transferase Mu 1 | GSTM1 |
| 8 | Superoxide dismutase [Mn], mitochondrial | SOD2 | 54 | E3 ubiquitin-protein ligase Mdm2 | MDM2 |
| 9 | Fibroblast growth factor receptor 1 | FGFR1 | 55 | Epidermal growth factor receptor erbB1 | EGFR |
| 10 | UDP-glucuronosyltransferase 1-1 | UGT1A1 | 56 | Interleukin-2 | IL2 |
| 11 | Neutrophil elastase | ELANE | 57 | Proto-oncogene c-Fos | FOS |
| 12 | Aldehyde dehydrogenase family 3 member A2 | ALDH3A2 | 58 | Interleukin-4 | IL4 |
| 13 | Amine oxidase [flavin-containing] A | MAOA | 59 | Insulin-like growth factor 1 receptor | IGF1R |
| 14 | Apolipoprotein B-100 | APOB | 60 | Histone deacetylase 8 | HDAC8 |
| 15 | Microsomal triglyceride transfer protein large subunit | MTTP | 61 | Intercellular adhesion molecule 1 | ICAM1 |
| 16 | Interleukin-8 | CXCL8 | 62 | Hypoxanthine-guanine phosphoribosyltransferase | HPRT1 |
| 17 | E-selectin | SELE | 63 | Serine/threonine-protein kinase Chk2 | CHEK2 |
| 18 | Fibronectin | FN1 | 64 | Hypoxia-inducible factor 1-alpha | HIF1A |
| 19 | Endothelin-converting enzyme 1 | ECE1 | 65 | Nitric oxide synthase, endothelial | NOS3 |
| 20 | Aromatase | CYP19A1 | 66 | Cystic fibrosis transmembrane conductance regulator | CFTR |
| 21 | Mitogen-activated protein kinase 14 | MAPK14 | 67 | Ubiquitin carboxyl-terminal hydrolase BAP1 | BAP1 |
| 22 | Tumor necrosis factor | TNF | 68 | C-C motif chemokine 5 | CCL5 |
| 23 | RAF proto-oncogene serine/threonine-protein kinase | RAF1 | 69 | Cellular tumor antigen p53 | TP53 |
| 24 | Low-density lipoprotein receptor | LDLR | 70 | Cyclin-dependent kinase inhibitor 1 | CDKN1A |
| 25 | Mast/stem cell growth factor receptor | KIT | 71 | Catalase | CAT |
| 26 | Signal transducer and activator of transcription 3 | STAT3 | 72 | RAC-alpha serine/threonine-protein kinase | AKT1 |
| 27 | Thrombin | F2 | 73 | mRNA of PKA Catalytic Subunit C-alpha | PRKACA |
| 28 | Baculoviral IAP repeat-containing protein 4 | XIAP | 74 | Somatotropin | GH1 |
| 29 | Thromboxane-A synthase | TBXAS1 | 75 | Interleukin-1 beta | IL1β |
| 30 | Prostaglandin G/H synthase 2 | PTGS2 | 76 | Renin | REN |
| 31 | Protein-glutamine gamma-glutamyltransferase E | TGM3 | 77 | Protein disulfide-isomerase | P4HB |
| 32 | Cell division control protein 42 homolog | CDC42 | 78 | Corticosteroid 11-beta-dehydrogenase isozyme 1 | HSD11B1 |
| 33 | Cyclin-dependent kinase 4 | CDK4 | 79 | DNA nucleotidylexotransferase | DNTT |
| 34 | GTPase HRas | HRAS | 80 | Apolipoprotein A-I | APOA1 |
| 35 | CGMP-inhibited 3',5'-cyclic phosphodiesterase A | PDE3A | 81 | Cyclin-dependent kinase inhibitor 2A, isoforms 1/2/3 | CDKN2A |
| 36 | Beta-2 adrenergic receptor | ADRB2 | 82 | Transcription factor AP-1 | JUN |
| 37 | Vascular endothelial growth factor A | VEGFA | 83 | Dihydroorotate dehydrogenase, mitochondrial | DHODH |
| 38 | Transforming growth factor beta-1 | TGFB1 | 84 | Calmodulin | CALM1 |
| 39 | Neprilysin | MME | 85 | B-Raf proto-oncogene serine/threonine-protein kinase | BRAF |
| 40 | Cyclin-A2 | CCNA2 | 86 | Apoptosis regulator BAX | BAX |
| 41 | Metalloproteinase inhibitor 1 | TIMP1 | 87 | Prostate-specific antigen | KLK3 |
| 42 | NADH-ubiquinone oxidoreductase chain 6 | MT-ND6 | 88 | Cytochrome P450 3A4 | CYP3A4 |
| 43 | Calmodulin | CALM2 | 89 | Amyloid beta A4 protein | APP |
| 44 | Acetylcholinesterase | ACHE | 90 | Histone deacetylase 6 | HDAC6 |
| 45 | C-C motif chemokine 2 | CCL2 | 91 | PMS1 protein homolog 2 | PMS2 |
| 46 | Serine hydroxymethyltransferase, cytosolic | SHMT1 | 92 | Matrix metalloproteinase-9 | MMP9 |

**Table S4** The data of GO (biological process) enrichment analysis of GATE candidate targets on NS treatment.

| GOID | GOTerm | Ontology Source | Term PValue | Term PValue Corrected with Benjamini-Hochberg | Group PValue | Group PValue Corrected with Benjamini-Hochberg | GOGroups | % Associated Genes | Nr. Genes | Associated Genes Found |
| --- | --- | --- | --- | --- | --- | --- | --- | --- | --- | --- |
| GO:0030335 | positive regulation of cell migration | GO_BiologicalProcess-EBI-UniProt-GOA_27.02.2019_00h00 | 3.82E-26 | 1.64E-23 | 3.82E-26 | 4.12E-24 | Group060 | 5.29 | 34 | [AKT1, APP, CCL5, CCNA2, CXCL8, EGFR, ELANE, FGFR1, FN1, HDAC6, HIF1A, HRAS, ICAM1, IGF1, IGF1R, IL1B, IL6, INS, JUN, KDR, KIT, MAPK14, MDM2, MMP9, MTTP, NOS3, PRKACA, PTGS2, SOD2, STAT3, TGFB1, TGFB2, TNF, VEGFA] |
| GO:0032496 | response to lipopolysaccharide | GO_BiologicalProcess-EBI-UniProt-GOA_27.02.2019_00h00 | 8.03E-22 | 1.72E-19 | 8.03E-22 | 4.34E-20 | Group064 | 5.90 | 27 | [ADRB2, AKT1, APOB, CASP3, CCL2, CCL5, CDK4, CXCL8, ELANE, FOS, HSP90AA1, ICAM1, IL1B, IL6, JUN, MAPK14, MTTP, NOS2, NOS3, PRKACA, PTGS2, REN, SELE, TGFB1, TIMP1, TNF, UGT1A1] |
| GO:0033674 | positive regulation of kinase activity | GO_BiologicalProcess-EBI-UniProt-GOA_27.02.2019_00h00 | 2.98E-21 | 4.27E-19 | 2.98E-21 | 1.07E-19 | Group106 | 4.13 | 32 | [ADRB2, AKT1, BRAF, CALM1, CALM2, CCL5, CCNA2, CDKN1A, EGFR, ELANE, F2, FGFR1, GH1, HRAS, HSP90AA1, IGF1, IGF1R, IL1B, IL2, IL4, IL6, INS, KIT, MAPK14, MTTP, PRKACA, RAF1, TGFB1, TGFB2, TIMP1, TNF, VEGFA] |
| GO:0043410 | positive regulation of MAPK cascade | GO_BiologicalProcess-EBI-UniProt-GOA_27.02.2019_00h00 | 4.52E-21 | 4.85E-19 | 4.52E-21 | 1.22E-19 | Group012 | 4.30 | 31 | [ADRB2, APP, BRAF, CCL2, CCL5, CDKN1A, EGFR, ELANE, FGFR1, GH1, HMGCR, HRAS, ICAM1, IGF1, IGF1R, IL1B, IL6, INS, JUN, KDR, KIT, MAPK14, MTTP, PRKACA, PTEN, RAF1, TGFB1, TGFB2, TIMP1, TNF, VEGFA] |
| GO:0045860 | positive regulation of protein kinase activity | GO_BiologicalProcess-EBI-UniProt-GOA_27.02.2019_00h00 | 5.55E-20 | 4.77E-18 | 2.98E-21 | 1.07E-19 | Group106 | 4.17 | 30 | [ADRB2, AKT1, BRAF, CALM1, CALM2, CCL5, CCNA2, CDKN1A, EGFR, ELANE, FGFR1, GH1, HRAS, HSP90AA1, IGF1, IGF1R, IL1B, IL4, IL6, INS, KIT, MAPK14, MTTP, PRKACA, RAF1, TGFB1, TGFB2, TIMP1, TNF, VEGFA] |
| GO:0009636 | response to toxic substance | GO_BiologicalProcess-EBI-UniProt-GOA_27.02.2019_00h00 | 3.97E-19 | 2.84E-17 | 3.97E-19 | 8.57E-18 | Group052 | 4.11 | 29 | [BAX, CALM1, CALM2, CASP3, CAT, CCL5, CDK4, CDKN1A, FOS, GSTM1, HDAC6, HMGCR, HPRT1, ICAM1, IL2, IL6, JUN, KDR, MDM2, MTTP, NOS3, PTEN, PTGS2, SOD2, STAT3, TBXAS1, TIMP1, TNF, UGT1A1] |
| GO:0050727 | regulation of inflammatory response | GO_BiologicalProcess-EBI-UniProt-GOA_27.02.2019_00h00 | 1.54E-18 | 9.43E-17 | 1.54E-18 | 2.37E-17 | Group017 | 5.42 | 24 | [ADRB2, APOA1, APP, BAP1, CCL5, CYP19A1, EGFR, ELANE, F2, IGF1, IL1B, IL2, IL4, IL6, INS, KLK3, LDLR, MAPK14, MMP9, NOS2, PTGS2, SELE, TNF, XIAP] |
| GO:0032147 | activation of protein kinase activity | GO_BiologicalProcess-EBI-UniProt-GOA_27.02.2019_00h00 | 2.26E-18 | 1.21E-16 | 2.98E-21 | 1.07E-19 | Group106 | 4.95 | 25 | [ADRB2, AKT1, BRAF, CALM1, CALM2, CCL5, CDKN1A, EGFR, GH1, IGF1, IGF1R, IL1B, IL4, IL6, INS, KIT, MAPK14, MTTP, PRKACA, RAF1, TGFB1, TGFB2, TIMP1, TNF, VEGFA] |
| GO:0072593 | reactive oxygen species metabolic process | GO_BiologicalProcess-EBI-UniProt-GOA_27.02.2019_00h00 | 3.15E-18 | 1.50E-16 | 3.15E-18 | 4.25E-17 | Group100 | 6.18 | 22 | [AKT1, CAT, CCNA2, CDKN1A, EGFR, F2, HDAC6, HIF1A, HSP90AA1, ICAM1, IL1B, INS, MAPK14, NOS2, NOS3, PTGS2, SOD2, STAT3, TGFB1, TIMP1, TNF, TP53] |
| GO:0009314 | response to radiation | GO_BiologicalProcess-EBI-UniProt-GOA_27.02.2019_00h00 | 8.92E-18 | 3.83E-16 | 8.92E-18 | 1.07E-16 | Group085 | 4.37 | 26 | [AKT1, APP, BAX, BRAF, CALM1, CALM2, CASP3, CAT, CDKN1A, CHEK2, EGFR, ELANE, EP300, FOS, HIF1A, HMGCR, HRAS, ICAM1, JUN, KIT, MAPK14, MDM2, MME, PTGS2, TGFB1, TP53] |
| GO:0045785 | positive regulation of cell adhesion | GO_BiologicalProcess-EBI-UniProt-GOA_27.02.2019_00h00 | 9.61E-17 | 3.75E-15 | 6.31E-19 | 1.14E-17 | Group111 | 4.52 | 24 | [AKT1, APOA1, BRAF, CCL2, CCL5, CCNA2, CDC42, ELANE, FN1, ICAM1, IGF1, IL1B, IL2, IL4, IL6, KDR, MTTP, PRKACA, TGFB1, TGFB2, TIMP1, TNF, VEGFA, ZAP70] |
| GO:0009416 | response to light stimulus | GO_BiologicalProcess-EBI-UniProt-GOA_27.02.2019_00h00 | 1.89E-16 | 6.74E-15 | 8.92E-18 | 1.07E-16 | Group085 | 5.09 | 22 | [AKT1, APP, BAX, BRAF, CALM1, CALM2, CASP3, CAT, CDKN1A, CHEK2, EGFR, ELANE, EP300, FOS, HIF1A, HMGCR, HRAS, KIT, MDM2, MME, PTGS2, TP53] |
| GO:0014065 | phosphatidylinositol 3-kinase signaling | GO_BiologicalProcess-EBI-UniProt-GOA_27.02.2019_00h00 | 3.27E-16 | 1.08E-14 | 3.27E-16 | 3.54E-15 | Group086 | 8.13 | 17 | [AKT1, CAT, CCL5, EGFR, F2, FGFR1, GH1, IGF1, IGF1R, IL6, INS, KDR, KIT, PTEN, TGFB2, TIMP1, TNF] |
| GO:0018108 | peptidyl-tyrosine phosphorylation | GO_BiologicalProcess-EBI-UniProt-GOA_27.02.2019_00h00 | 3.64E-16 | 1.12E-14 | 3.64E-16 | 3.58E-15 | Group095 | 4.57 | 23 | [APP, BRAF, CCL5, EGFR, FGFR1, GH1, ICAM1, IGF1, IGF1R, IL2, IL4, IL6, KDR, KIT, MME, MTTP, STAT3, TGFB1, TIMP1, TNF, TP53, VEGFA, ZAP70] |
| GO:0035690 | cellular response to drug | GO_BiologicalProcess-EBI-UniProt-GOA_27.02.2019_00h00 | 5.13E-16 | 1.47E-14 | 5.13E-16 | 4.61E-15 | Group006 | 4.50 | 23 | [BRAF, CCNA2, CDK4, CDKN1A, CDKN2A, CFTR, CHEK2, EGFR, HDAC6, ICAM1, IL1B, IL6, KDR, MDM2, MTTP, NOS2, PTEN, PTGS2, REN, TGFB1, TNF, TP53, UGT1A1] |
| GO:0014068 | positive regulation of phosphatidylinositol 3-kinase signaling | GO_BiologicalProcess-EBI-UniProt-GOA_27.02.2019_00h00 | 1.29E-15 | 3.47E-14 | 3.27E-16 | 3.54E-15 | Group086 | 11.48 | 14 | [CAT, CCL5, F2, FGFR1, GH1, IGF1, IGF1R, IL6, INS, KDR, KIT, TGFB2, TIMP1, TNF] |
| GO:0070371 | ERK1 and ERK2 cascade | GO_BiologicalProcess-EBI-UniProt-GOA_27.02.2019_00h00 | 3.51E-15 | 8.85E-14 | 3.51E-15 | 2.70E-14 | Group074 | 4.78 | 21 | [APP, BRAF, CCL2, CCL5, CCNA2, EGFR, FN1, HMGCR, HRAS, ICAM1, IGF1, IL1B, JUN, KDR, KIT, MTTP, PRKACA, PTEN, TGFB1, TIMP1, TNF] |
| GO:0004517 | nitric-oxide synthase activity | GO_BiologicalProcess-EBI-UniProt-GOA_27.02.2019_00h00 | 5.65E-15 | 1.35E-13 | 1.44E-15 | 1.20E-14 | Group107 | 15.00 | 12 | [AKT1, CALM1, CALM2, EGFR, HIF1A, HRAS, HSP90AA1, IL1B, INS, NOS2, NOS3, TNF] |
| GO:0001667 | ameboidal-type cell migration | GO_BiologicalProcess-EBI-UniProt-GOA_27.02.2019_00h00 | 7.82E-15 | 1.68E-13 | 7.82E-15 | 5.28E-14 | Group108 | 4.26 | 22 | [AKT1, APOA1, BRAF, FGFR1, FN1, HDAC6, HIF1A, IGF1, IL4, JUN, KDR, KIT, MMP9, MTTP, NOS3, PRKACA, PTEN, PTGS2, TGFB1, TGFB2, TIMP1, VEGFA] |
| GO:0048872 | homeostasis of number of cells | GO_BiologicalProcess-EBI-UniProt-GOA_27.02.2019_00h00 | 7.52E-15 | 1.70E-13 | 7.52E-15 | 5.41E-14 | Group016 | 5.48 | 19 | [AKT1, BAP1, BAX, CASP3, HIF1A, HRAS, IGF1, IL2, IL6, KIT, KLK3, LDLR, MAPK14, MTTP, NOS3, STAT3, TGFB1, TIMP1, VEGFA] |
| GO:0048660 | regulation of smooth muscle cell proliferation | GO_BiologicalProcess-EBI-UniProt-GOA_27.02.2019_00h00 | 8.81E-15 | 1.72E-13 | 8.81E-15 | 5.60E-14 | Group066 | 8.62 | 15 | [AKT1, CCL5, CDKN1A, EGFR, ELANE, HMGCR, IGF1, IL6, JUN, MDM2, MMP9, PTEN, PTGS2, SOD2, TNF] |
| GO:0034599 | cellular response to oxidative stress | GO_BiologicalProcess-EBI-UniProt-GOA_27.02.2019_00h00 | 8.71E-15 | 1.78E-13 | 1.29E-14 | 7.77E-14 | Group092 | 4.96 | 20 | [AKT1, CAT, CCNA2, CDKN1A, CDKN2A, EGFR, FOS, HDAC6, HIF1A, IL6, INS, JUN, MDM2, MMP9, MTTP, NOS3, P4HB, SOD2, TNF, TP53] |
| GO:0071902 | positive regulation of protein serine/threonine kinase activity | GO_BiologicalProcess-EBI-UniProt-GOA_27.02.2019_00h00 | 1.19E-14 | 2.22E-13 | 2.98E-21 | 1.07E-19 | Group106 | 4.50 | 21 | [ADRB2, AKT1, BRAF, CALM1, CALM2, CDKN1A, EGFR, ELANE, FGFR1, GH1, HRAS, IGF1, IGF1R, IL1B, IL6, KIT, MAPK14, RAF1, TGFB1, TNF, VEGFA] |
| GO:0016705 | oxidoreductase activity, acting on paired donors, with incorporation or reduction of molecular oxygen | GO_BiologicalProcess-EBI-UniProt-GOA_27.02.2019_00h00 | 2.49E-14 | 4.44E-13 | 1.44E-15 | 1.20E-14 | Group107 | 6.27 | 17 | [AKT1, CALM1, CALM2, CYP19A1, CYP3A4, EGFR, HIF1A, HRAS, HSP90AA1, IL1B, INS, NOS2, NOS3, P4HB, PTGS2, TBXAS1, TNF] |
| GO:2000377 | regulation of reactive oxygen species metabolic process | GO_BiologicalProcess-EBI-UniProt-GOA_27.02.2019_00h00 | 4.05E-14 | 6.95E-13 | 3.15E-18 | 4.25E-17 | Group100 | 6.84 | 16 | [AKT1, CDKN1A, EGFR, F2, HDAC6, HIF1A, HSP90AA1, ICAM1, IL1B, INS, MAPK14, PTGS2, STAT3, TGFB1, TNF, TP53] |
| GO:0004497 | monooxygenase activity | GO_BiologicalProcess-EBI-UniProt-GOA_27.02.2019_00h00 | 6.01E-14 | 9.92E-13 | 1.44E-15 | 1.20E-14 | Group107 | 7.58 | 15 | [AKT1, CALM1, CALM2, CYP19A1, CYP3A4, EGFR, HIF1A, HRAS, HSP90AA1, IL1B, INS, NOS2, NOS3, TBXAS1, TNF] |
| GO:0070374 | positive regulation of ERK1 and ERK2 cascade | GO_BiologicalProcess-EBI-UniProt-GOA_27.02.2019_00h00 | 6.38E-14 | 1.01E-12 | 3.51E-15 | 2.70E-14 | Group074 | 5.92 | 17 | [APP, BRAF, CCL2, CCL5, EGFR, HMGCR, HRAS, ICAM1, JUN, KDR, KIT, MTTP, PRKACA, PTEN, TGFB1, TIMP1, TNF] |
| GO:0070482 | response to oxygen levels | GO_BiologicalProcess-EBI-UniProt-GOA_27.02.2019_00h00 | 8.09E-14 | 1.24E-12 | 8.09E-14 | 4.16E-13 | Group096 | 4.08 | 21 | [AKT1, CASP3, CAT, CCNA2, CDK4, CDKN1A, EP300, HIF1A, ICAM1, KLK3, MDM2, NOS2, P4HB, PTEN, PTGS2, RAF1, TGFB1, TGFB2, TIMP1, TP53, VEGFA] |
| GO:0051341 | regulation of oxidoreductase activity | GO_BiologicalProcess-EBI-UniProt-GOA_27.02.2019_00h00 | 8.82E-14 | 1.30E-12 | 1.44E-15 | 1.20E-14 | Group107 | 10.00 | 13 | [AKT1, CALM1, CALM2, EGFR, HDAC6, HIF1A, HRAS, HSP90AA1, IL1B, INS, MTTP, NOS3, TNF] |
| GO:0018105 | peptidyl-serine phosphorylation | GO_BiologicalProcess-EBI-UniProt-GOA_27.02.2019_00h00 | 9.33E-14 | 1.33E-12 | 9.33E-14 | 4.58E-13 | Group090 | 4.76 | 19 | [AKT1, APP, BAX, BRAF, CDKN1A, CDKN2A, CHEK2, EGFR, HDAC6, HSP90AA1, IL6, MAPK14, PRKACA, PTGS2, RAF1, TGFB1, TIMP1, TNF, VEGFA] |
| GO:0022407 | regulation of cell-cell adhesion | GO_BiologicalProcess-EBI-UniProt-GOA_27.02.2019_00h00 | 1.09E-13 | 1.51E-12 | 6.31E-19 | 1.14E-17 | Group111 | 4.02 | 21 | [AKT1, APOA1, CASP3, CCL2, CCL5, CDC42, CDKN2A, ELANE, ICAM1, IGF1, IL1B, IL2, IL4, IL6, MAPK14, MTTP, PRKACA, TGFB1, TIMP1, TNF, ZAP70] |
| GO:0032103 | positive regulation of response to external stimulus | GO_BiologicalProcess-EBI-UniProt-GOA_27.02.2019_00h00 | 1.17E-13 | 1.56E-12 | 1.17E-13 | 5.48E-13 | Group062 | 4.70 | 19 | [APP, BRAF, CCL5, CXCL8, EGFR, F2, FGFR1, IL1B, IL2, IL6, KDR, KLK3, LDLR, MAPK14, PRKACA, PTGS2, TGFB1, TNF, VEGFA] |
| GO:0000302 | response to reactive oxygen species | GO_BiologicalProcess-EBI-UniProt-GOA_27.02.2019_00h00 | 1.63E-13 | 2.12E-12 | 1.29E-14 | 7.77E-14 | Group092 | 5.59 | 17 | [AKT1, CASP3, CAT, CCNA2, CDKN1A, CDKN2A, EGFR, FOS, HDAC6, IL6, JUN, MDM2, MMP9, MTTP, NOS3, SOD2, TNF] |
| GO:0071216 | cellular response to biotic stimulus | GO_BiologicalProcess-EBI-UniProt-GOA_27.02.2019_00h00 | 2.36E-13 | 2.98E-12 | 2.36E-13 | 1.06E-12 | Group075 | 5.47 | 17 | [ADRB2, AKT1, CCL2, CCL5, CDK4, CXCL8, ICAM1, IL1B, IL6, MAPK14, MTTP, NOS2, NOS3, PRKACA, TGFB1, TNF, TP53] |
| GO:0034614 | cellular response to reactive oxygen species | GO_BiologicalProcess-EBI-UniProt-GOA_27.02.2019_00h00 | 3.93E-13 | 4.81E-12 | 1.29E-14 | 7.77E-14 | Group092 | 6.67 | 15 | [AKT1, CCNA2, CDKN1A, CDKN2A, EGFR, FOS, HDAC6, IL6, JUN, MDM2, MMP9, MTTP, NOS3, SOD2, TNF] |
| GO:0010631 | epithelial cell migration | GO_BiologicalProcess-EBI-UniProt-GOA_27.02.2019_00h00 | 4.53E-13 | 5.40E-12 | 7.82E-15 | 5.28E-14 | Group108 | 4.76 | 18 | [AKT1, APOA1, FGFR1, HDAC6, HIF1A, IL4, JUN, KDR, KIT, MMP9, MTTP, NOS3, PRKACA, PTEN, PTGS2, TGFB1, TGFB2, VEGFA] |
| GO:0051384 | response to glucocorticoid | GO_BiologicalProcess-EBI-UniProt-GOA_27.02.2019_00h00 | 5.08E-13 | 5.73E-12 | 5.08E-13 | 2.19E-12 | Group022 | 6.55 | 15 | [CALM1, CALM2, CASP3, CDKN1A, EGFR, FOS, HRAS, ICAM1, IL6, PTGS2, TBXAS1, TGFB1, TIMP1, TNF, UGT1A1] |
| GO:0071222 | cellular response to lipopolysaccharide | GO_BiologicalProcess-EBI-UniProt-GOA_27.02.2019_00h00 | 5.22E-13 | 5.74E-12 | 2.36E-13 | 1.06E-12 | Group075 | 5.80 | 16 | [ADRB2, AKT1, CCL2, CCL5, CDK4, CXCL8, ICAM1, IL1B, IL6, MAPK14, MTTP, NOS2, NOS3, PRKACA, TGFB1, TNF] |
| GO:0048661 | positive regulation of smooth muscle cell proliferation | GO_BiologicalProcess-EBI-UniProt-GOA_27.02.2019_00h00 | 5.02E-13 | 5.82E-12 | 8.81E-15 | 5.60E-14 | Group066 | 10.43 | 12 | [AKT1, CCL5, EGFR, ELANE, HMGCR, IGF1, IL6, JUN, MDM2, MMP9, PTGS2, TNF] |
| GO:0010634 | positive regulation of epithelial cell migration | GO_BiologicalProcess-EBI-UniProt-GOA_27.02.2019_00h00 | 6.47E-13 | 6.94E-12 | 7.82E-15 | 5.28E-14 | Group108 | 7.37 | 14 | [AKT1, FGFR1, HDAC6, HIF1A, JUN, KDR, MMP9, MTTP, NOS3, PRKACA, PTGS2, TGFB1, TGFB2, VEGFA] |
| GO:0097193 | intrinsic apoptotic signaling pathway | GO_BiologicalProcess-EBI-UniProt-GOA_27.02.2019_00h00 | 7.37E-13 | 7.71E-12 | 7.37E-13 | 2.95E-12 | Group101 | 4.63 | 18 | [AKT1, BAX, CASP3, CDKN1A, CHEK2, EP300, HIF1A, HRAS, INS, MDM2, MMP9, MTTP, P4HB, PTGS2, SOD2, TIMP1, TNF, TP53] |
| GO:0071214 | cellular response to abiotic stimulus | GO_BiologicalProcess-EBI-UniProt-GOA_27.02.2019_00h00 | 8.57E-13 | 8.75E-12 | 8.57E-13 | 3.31E-12 | Group076 | 4.20 | 19 | [AKT1, BAX, CALM1, CALM2, CASP3, CDKN1A, CHEK2, EGFR, EP300, HRAS, IL1B, MAPK14, MDM2, MME, PTEN, PTGS2, TGFB1, TIMP1, TP53] |
| GO:0050890 | cognition | GO_BiologicalProcess-EBI-UniProt-GOA_27.02.2019_00h00 | 9.95E-13 | 9.93E-12 | 9.95E-13 | 3.71E-12 | Group069 | 4.55 | 18 | [APP, BRAF, CALM2, CASP3, EGFR, EP300, FOS, HIF1A, HMGCR, HRAS, INS, JUN, KIT, LDLR, MME, PTEN, PTGS2, TNF] |
| GO:0010632 | regulation of epithelial cell migration | GO_BiologicalProcess-EBI-UniProt-GOA_27.02.2019_00h00 | 1.11E-12 | 1.06E-11 | 7.82E-15 | 5.28E-14 | Group108 | 5.52 | 16 | [AKT1, FGFR1, HDAC6, HIF1A, IL4, JUN, KDR, MMP9, MTTP, NOS3, PRKACA, PTEN, PTGS2, TGFB1, TGFB2, VEGFA] |
| GO:0050730 | regulation of peptidyl-tyrosine phosphorylation | GO_BiologicalProcess-EBI-UniProt-GOA_27.02.2019_00h00 | 1.10E-12 | 1.07E-11 | 3.64E-16 | 3.58E-15 | Group095 | 4.97 | 17 | [APP, CCL5, EGFR, GH1, ICAM1, IGF1, IL2, IL4, IL6, KIT, MTTP, STAT3, TGFB1, TIMP1, TNF, TP53, VEGFA] |
| GO:0090068 | positive regulation of cell cycle process | GO_BiologicalProcess-EBI-UniProt-GOA_27.02.2019_00h00 | 1.18E-12 | 1.10E-11 | 5.54E-13 | 2.30E-12 | Group077 | 4.50 | 18 | [AKT1, APP, BAX, CDC42, CDK4, CDKN1A, CDKN2A, CHEK2, EGFR, EP300, FGFR1, IGF1, IL1B, INS, MDM2, PRKACA, TGFB1, TP53] |
| GO:0001666 | response to hypoxia | GO_BiologicalProcess-EBI-UniProt-GOA_27.02.2019_00h00 | 1.21E-12 | 1.11E-11 | 8.09E-14 | 4.16E-13 | Group096 | 4.12 | 19 | [AKT1, CASP3, CAT, CCNA2, EP300, HIF1A, ICAM1, KLK3, MDM2, NOS2, P4HB, PTEN, PTGS2, RAF1, TGFB1, TGFB2, TIMP1, TP53, VEGFA] |
| GO:2000379 | positive regulation of reactive oxygen species metabolic process | GO_BiologicalProcess-EBI-UniProt-GOA_27.02.2019_00h00 | 1.52E-12 | 1.35E-11 | 3.15E-18 | 4.25E-17 | Group100 | 9.52 | 12 | [AKT1, CDKN1A, EGFR, F2, HSP90AA1, ICAM1, IL1B, MAPK14, PTGS2, TGFB1, TNF, TP53] |
| GO:0050731 | positive regulation of peptidyl-tyrosine phosphorylation | GO_BiologicalProcess-EBI-UniProt-GOA_27.02.2019_00h00 | 1.61E-12 | 1.41E-11 | 3.64E-16 | 3.58E-15 | Group095 | 6.05 | 15 | [CCL5, GH1, ICAM1, IGF1, IL2, IL4, IL6, KIT, MTTP, STAT3, TGFB1, TIMP1, TNF, TP53, VEGFA] |
| GO:0006066 | alcohol metabolic process | GO_BiologicalProcess-EBI-UniProt-GOA_27.02.2019_00h00 | 1.77E-12 | 1.52E-11 | 7.11E-14 | 3.84E-13 | Group104 | 4.03 | 19 | [ADRB2, ALDH3A2, APOA1, APOB, APP, CALM1, CALM2, CAT, CFTR, CYP3A4, FGFR1, HMGCR, IGF1, IL1B, IL4, LDLR, PTEN, TNF, ZAP70] |
| GO:2001234 | negative regulation of apoptotic signaling pathway | GO_BiologicalProcess-EBI-UniProt-GOA_27.02.2019_00h00 | 2.40E-12 | 2.02E-11 | 2.40E-12 | 8.65E-12 | Group041 | 5.25 | 16 | [AKT1, BAX, HIF1A, ICAM1, IGF1, IL1B, IL6, INS, MDM2, MMP9, NOS3, PTGS2, RAF1, SOD2, TIMP1, TNF] |
| GO:0002526 | acute inflammatory response | GO_BiologicalProcess-EBI-UniProt-GOA_27.02.2019_00h00 | 2.64E-12 | 2.18E-11 | 2.64E-12 | 9.21E-12 | Group084 | 7.69 | 13 | [ELANE, F2, FN1, ICAM1, IL1B, IL6, INS, KLK3, PTGS2, STAT3, TIMP1, TNF, UGT1A1] |
| GO:0008202 | steroid metabolic process | GO_BiologicalProcess-EBI-UniProt-GOA_27.02.2019_00h00 | 2.78E-12 | 2.25E-11 | 7.11E-14 | 3.84E-13 | Group104 | 4.28 | 18 | [ADRB2, APOA1, APOB, APP, CAT, CFTR, CYP19A1, CYP3A4, FGFR1, HMGCR, HSD11B1, IL1B, IL4, KIT, LDLR, TNF, UGT1A1, ZAP70] |
| GO:0032102 | negative regulation of response to external stimulus | GO_BiologicalProcess-EBI-UniProt-GOA_27.02.2019_00h00 | 3.67E-12 | 2.91E-11 | 3.67E-12 | 1.24E-11 | Group067 | 4.21 | 18 | [ADRB2, APOA1, CCL2, CDKN1A, CYP19A1, ELANE, F2, GP1BA, HMGCR, IGF1, IL2, IL4, IL6, INS, KLK3, LDLR, NOS3, PTEN] |
| GO:0043406 | positive regulation of MAP kinase activity | GO_BiologicalProcess-EBI-UniProt-GOA_27.02.2019_00h00 | 4.20E-12 | 3.28E-11 | 2.98E-21 | 1.07E-19 | Group106 | 4.57 | 17 | [BRAF, CDKN1A, EGFR, ELANE, FGFR1, GH1, HRAS, IGF1, IGF1R, IL1B, IL6, KIT, MAPK14, RAF1, TGFB1, TNF, VEGFA] |
| GO:0097305 | response to alcohol | GO_BiologicalProcess-EBI-UniProt-GOA_27.02.2019_00h00 | 4.75E-12 | 3.64E-11 | 4.75E-12 | 1.55E-11 | Group029 | 5.02 | 16 | [AKT1, CALM1, CALM2, CAT, CDK4, CDKN1A, CFTR, FOS, HMGCR, ICAM1, IL2, PTEN, STAT3, TBXAS1, TGFB1, UGT1A1] |
| GO:0007159 | leukocyte cell-cell adhesion | GO_BiologicalProcess-EBI-UniProt-GOA_27.02.2019_00h00 | 5.60E-12 | 4.21E-11 | 6.31E-19 | 1.14E-17 | Group111 | 4.10 | 18 | [AKT1, CASP3, CCL2, CCL5, CDC42, CDKN2A, ELANE, ICAM1, IGF1, IL1B, IL2, IL4, IL6, SELE, TGFB1, TIMP1, TNF, ZAP70] |
| GO:0007584 | response to nutrient | GO_BiologicalProcess-EBI-UniProt-GOA_27.02.2019_00h00 | 7.56E-12 | 5.59E-11 | 7.56E-12 | 2.33E-11 | Group046 | 4.86 | 16 | [ADRB2, APOA1, CAT, DHODH, DNTT, EGFR, HMGCR, IL1B, INS, MDM2, PTEN, PTGS2, TBXAS1, TGFB1, TIMP1, UGT1A1] |
| GO:0046777 | protein autophosphorylation | GO_BiologicalProcess-EBI-UniProt-GOA_27.02.2019_00h00 | 8.29E-12 | 6.02E-11 | 8.29E-12 | 2.49E-11 | Group038 | 4.83 | 16 | [AKT1, CALM1, CALM2, CHEK2, EGFR, FGFR1, IGF1R, INS, JUN, KDR, KIT, MME, MTTP, PRKACA, VEGFA, ZAP70] |
| GO:0043491 | protein kinase B signaling | GO_BiologicalProcess-EBI-UniProt-GOA_27.02.2019_00h00 | 1.24E-11 | 8.87E-11 | 1.24E-11 | 3.53E-11 | Group072 | 4.71 | 16 | [AKT1, CCL2, CCL5, EGFR, FGFR1, HSP90AA1, IGF1, IGF1R, IL1B, IL6, INS, KDR, KIT, PTEN, TGFB1, TNF] |
| GO:0150076 | neuroinflammatory response | GO_BiologicalProcess-EBI-UniProt-GOA_27.02.2019_00h00 | 1.27E-11 | 8.94E-11 | 1.66E-14 | 9.45E-14 | Group110 | 11.90 | 10 | [APP, EGFR, IGF1, IL1B, IL4, IL6, JUN, LDLR, MMP9, TNF] |
| GO:1903039 | positive regulation of leukocyte cell-cell adhesion | GO_BiologicalProcess-EBI-UniProt-GOA_27.02.2019_00h00 | 1.31E-11 | 9.07E-11 | 6.31E-19 | 1.14E-17 | Group111 | 5.23 | 15 | [AKT1, CCL2, CCL5, CDC42, ELANE, ICAM1, IGF1, IL1B, IL2, IL4, IL6, TGFB1, TIMP1, TNF, ZAP70] |
| GO:0007611 | learning or memory | GO_BiologicalProcess-EBI-UniProt-GOA_27.02.2019_00h00 | 1.54E-11 | 1.05E-10 | 9.95E-13 | 3.71E-12 | Group069 | 4.64 | 16 | [APP, BRAF, CALM2, CASP3, EGFR, EP300, FOS, HIF1A, HMGCR, HRAS, JUN, KIT, LDLR, MME, PTEN, PTGS2] |
| GO:0001776 | leukocyte homeostasis | GO_BiologicalProcess-EBI-UniProt-GOA_27.02.2019_00h00 | 1.90E-11 | 1.27E-10 | 1.90E-11 | 5.25E-11 | Group089 | 9.24 | 11 | [AKT1, BAP1, BAX, CASP3, HIF1A, IGF1, IL2, IL6, KLK3, MTTP, TGFB1] |
| GO:0097191 | extrinsic apoptotic signaling pathway | GO_BiologicalProcess-EBI-UniProt-GOA_27.02.2019_00h00 | 2.13E-11 | 1.41E-10 | 2.13E-11 | 5.76E-11 | Group083 | 5.05 | 15 | [AKT1, BAX, CASP3, FGFR1, ICAM1, IGF1, IL1B, IL2, IL6, NOS3, PTEN, RAF1, TGFB1, TGFB2, TNF] |
| GO:0006953 | acute-phase response | GO_BiologicalProcess-EBI-UniProt-GOA_27.02.2019_00h00 | 2.29E-11 | 1.47E-10 | 2.64E-12 | 9.21E-12 | Group084 | 11.24 | 10 | [F2, FN1, IL1B, IL6, INS, PTGS2, STAT3, TIMP1, TNF, UGT1A1] |
| GO:0009743 | response to carbohydrate | GO_BiologicalProcess-EBI-UniProt-GOA_27.02.2019_00h00 | 2.35E-11 | 1.48E-10 | 2.35E-11 | 6.03E-11 | Group091 | 5.02 | 15 | [ADRB2, APOB, CASP3, CAT, CFTR, HIF1A, HMGCR, ICAM1, IGF1R, IL1B, PRKACA, PTEN, PTGS2, RAF1, TGFB1] |
| GO:1901214 | regulation of neuron death | GO_BiologicalProcess-EBI-UniProt-GOA_27.02.2019_00h00 | 2.28E-11 | 1.48E-10 | 2.28E-11 | 6.01E-11 | Group032 | 4.11 | 17 | [AKT1, BAX, BRAF, CALM2, CASP3, CCL2, CCL5, FOS, HIF1A, HRAS, JUN, MTTP, SOD2, STAT3, TGFB2, TIMP1, TNF] |
| GO:0033135 | regulation of peptidyl-serine phosphorylation | GO_BiologicalProcess-EBI-UniProt-GOA_27.02.2019_00h00 | 3.06E-11 | 1.90E-10 | 9.33E-14 | 4.58E-13 | Group090 | 6.34 | 13 | [AKT1, APP, BAX, BRAF, EGFR, HDAC6, HSP90AA1, IL6, PTGS2, RAF1, TGFB1, TNF, VEGFA] |
| GO:0007259 | receptor signaling pathway via JAK-STAT | GO_BiologicalProcess-EBI-UniProt-GOA_27.02.2019_00h00 | 3.68E-11 | 2.25E-10 | 3.68E-11 | 9.23E-11 | Group043 | 6.25 | 13 | [CCL2, CCL5, F2, GH1, IGF1, IL2, IL4, IL6, KIT, KLK3, STAT3, TIMP1, TNF] |
| GO:0002685 | regulation of leukocyte migration | GO_BiologicalProcess-EBI-UniProt-GOA_27.02.2019_00h00 | 3.86E-11 | 2.34E-10 | 3.86E-11 | 9.49E-11 | Group070 | 5.45 | 14 | [AKT1, APP, CCL2, CCL5, CXCL8, CYP19A1, ELANE, ICAM1, IGF1, IL6, MAPK14, TGFB1, TNF, VEGFA] |
| GO:1902652 | secondary alcohol metabolic process | GO_BiologicalProcess-EBI-UniProt-GOA_27.02.2019_00h00 | 4.67E-11 | 2.78E-10 | 7.11E-14 | 3.84E-13 | Group104 | 6.13 | 13 | [ADRB2, APOA1, APOB, APP, CAT, CFTR, CYP3A4, FGFR1, HMGCR, IL1B, IL4, LDLR, TNF] |
| GO:0042063 | gliogenesis | GO_BiologicalProcess-EBI-UniProt-GOA_27.02.2019_00h00 | 5.15E-11 | 3.03E-10 | 1.66E-14 | 9.45E-14 | Group110 | 4.28 | 16 | [AKT1, APP, CCL2, CDKN1A, EGFR, F2, HRAS, IL1B, IL6, LDLR, MTTP, PTEN, STAT3, TGFB1, TGFB2, TNF] |
| GO:0045931 | positive regulation of mitotic cell cycle | GO_BiologicalProcess-EBI-UniProt-GOA_27.02.2019_00h00 | 5.25E-11 | 3.04E-10 | 5.54E-13 | 2.30E-12 | Group077 | 6.07 | 13 | [AKT1, APP, CDK4, CDKN1A, EGFR, FGFR1, IGF1, IL1B, INS, MDM2, MTTP, PRKACA, TGFB1] |
| GO:0010001 | glial cell differentiation | GO_BiologicalProcess-EBI-UniProt-GOA_27.02.2019_00h00 | 9.05E-11 | 5.18E-10 | 1.66E-14 | 9.45E-14 | Group110 | 5.11 | 14 | [AKT1, APP, CDKN1A, EGFR, F2, HRAS, IL1B, IL6, LDLR, MTTP, PTEN, STAT3, TGFB1, TNF] |
| GO:0046879 | hormone secretion | GO_BiologicalProcess-EBI-UniProt-GOA_27.02.2019_00h00 | 9.22E-11 | 5.20E-10 | 9.22E-11 | 2.07E-10 | Group103 | 4.11 | 16 | [ADRB2, CCL5, CFTR, CYP19A1, EGFR, FGFR1, HIF1A, HMGCR, IL1B, IL6, INS, NOS2, PRKACA, RAF1, REN, TNF] |
| GO:0150077 | regulation of neuroinflammatory response | GO_BiologicalProcess-EBI-UniProt-GOA_27.02.2019_00h00 | 9.59E-11 | 5.34E-10 | 1.66E-14 | 9.45E-14 | Group110 | 16.67 | 8 | [APP, IGF1, IL1B, IL4, IL6, LDLR, MMP9, TNF] |
| GO:0001935 | endothelial cell proliferation | GO_BiologicalProcess-EBI-UniProt-GOA_27.02.2019_00h00 | 1.11E-10 | 6.09E-10 | 4.48E-11 | 1.08E-10 | Group094 | 6.63 | 12 | [AKT1, APOA1, CCL2, FGFR1, HIF1A, HRAS, JUN, KDR, PRKACA, STAT3, TNF, VEGFA] |
| GO:0003018 | vascular process in circulatory system | GO_BiologicalProcess-EBI-UniProt-GOA_27.02.2019_00h00 | 1.16E-10 | 6.29E-10 | 1.16E-10 | 2.55E-10 | Group097 | 5.70 | 13 | [ADRB2, AKT1, ECE1, EGFR, HMGCR, ICAM1, INS, NOS3, PDE3A, PTGS2, SOD2, TBXAS1, TGFB1] |
| GO:0051353 | positive regulation of oxidoreductase activity | GO_BiologicalProcess-EBI-UniProt-GOA_27.02.2019_00h00 | 1.32E-10 | 7.06E-10 | 1.44E-15 | 1.20E-14 | Group107 | 12.00 | 9 | [AKT1, CALM1, CALM2, HIF1A, HRAS, IL1B, INS, MTTP, TNF] |
| GO:0042133 | neurotransmitter metabolic process | GO_BiologicalProcess-EBI-UniProt-GOA_27.02.2019_00h00 | 1.62E-10 | 8.57E-10 | 8.72E-12 | 2.55E-11 | Group109 | 6.42 | 12 | [ACHE, AKT1, CAT, HSP90AA1, ICAM1, IL1B, MAOA, NOS2, NOS3, PTGS2, SHMT1, TNF] |
| GO:0048708 | astrocyte differentiation | GO_BiologicalProcess-EBI-UniProt-GOA_27.02.2019_00h00 | 1.94E-10 | 1.02E-09 | 1.66E-14 | 9.45E-14 | Group110 | 9.09 | 10 | [APP, EGFR, F2, HRAS, IL1B, IL6, LDLR, MTTP, STAT3, TNF] |
| GO:0045834 | positive regulation of lipid metabolic process | GO_BiologicalProcess-EBI-UniProt-GOA_27.02.2019_00h00 | 2.07E-10 | 1.07E-09 | 5.85E-11 | 1.37E-10 | Group102 | 6.28 | 12 | [ADRB2, AKT1, APOA1, CCNA2, F2, IL1B, INS, KIT, LDLR, PTGS2, TGFB1, TNF] |
| GO:1904018 | positive regulation of vasculature development | GO_BiologicalProcess-EBI-UniProt-GOA_27.02.2019_00h00 | 2.30E-10 | 1.18E-09 | 2.30E-10 | 4.97E-10 | Group081 | 5.39 | 13 | [CXCL8, HIF1A, IL1B, IL6, KDR, KIT, MTTP, NOS3, PRKACA, PTGS2, SOD2, STAT3, VEGFA] |
| GO:0062013 | positive regulation of small molecule metabolic process | GO_BiologicalProcess-EBI-UniProt-GOA_27.02.2019_00h00 | 2.33E-10 | 1.18E-09 | 2.33E-10 | 4.94E-10 | Group098 | 6.22 | 12 | [ADRB2, AKT1, CDKN1A, HIF1A, IGF1, IL1B, INS, NOS2, NOS3, PTGS2, STAT3, TNF] |
| GO:0097237 | cellular response to toxic substance | GO_BiologicalProcess-EBI-UniProt-GOA_27.02.2019_00h00 | 2.41E-10 | 1.20E-09 | 2.41E-10 | 5.01E-10 | Group028 | 4.25 | 15 | [CAT, CDKN1A, GSTM1, HDAC6, IL6, KDR, MDM2, MTTP, NOS3, PTEN, PTGS2, SOD2, TIMP1, TNF, UGT1A1] |
| GO:0046683 | response to organophosphorus | GO_BiologicalProcess-EBI-UniProt-GOA_27.02.2019_00h00 | 2.48E-10 | 1.22E-09 | 7.42E-11 | 1.70E-10 | Group073 | 6.19 | 12 | [APP, BRAF, CFTR, DNTT, FOS, IL1B, JUN, PDE3A, PTEN, PTGS2, REN, TBXAS1] |
| GO:0051091 | positive regulation of DNA-binding transcription factor activity | GO_BiologicalProcess-EBI-UniProt-GOA_27.02.2019_00h00 | 2.93E-10 | 1.43E-09 | 2.93E-10 | 5.97E-10 | Group021 | 4.19 | 15 | [AKT1, APP, CAT, EP300, HRAS, ICAM1, IL1B, IL6, INS, KIT, PTEN, STAT3, TGFB1, TNF, VEGFA] |
| GO:0009411 | response to UV | GO_BiologicalProcess-EBI-UniProt-GOA_27.02.2019_00h00 | 3.52E-10 | 1.70E-09 | 3.52E-10 | 7.04E-10 | Group049 | 6.00 | 12 | [AKT1, BAX, CASP3, CAT, CDKN1A, EGFR, ELANE, EP300, MDM2, MME, PTGS2, TP53] |
| GO:0071887 | leukocyte apoptotic process | GO_BiologicalProcess-EBI-UniProt-GOA_27.02.2019_00h00 | 3.60E-10 | 1.71E-09 | 3.60E-10 | 7.06E-10 | Group082 | 7.05 | 11 | [AKT1, BAX, CASP3, CCL5, CDKN2A, HIF1A, IGF1, IL2, IL6, PTEN, TP53] |
| GO:0031663 | lipopolysaccharide-mediated signaling pathway | GO_BiologicalProcess-EBI-UniProt-GOA_27.02.2019_00h00 | 3.72E-10 | 1.75E-09 | 3.72E-10 | 7.18E-10 | Group061 | 10.71 | 9 | [AKT1, CCL2, CCL5, IL1B, MAPK14, NOS3, PRKACA, TGFB1, TNF] |
| GO:0042098 | T cell proliferation | GO_BiologicalProcess-EBI-UniProt-GOA_27.02.2019_00h00 | 3.80E-10 | 1.77E-09 | 6.31E-19 | 1.14E-17 | Group111 | 5.18 | 13 | [BAX, CASP3, CCL5, CDKN2A, IGF1, IL1B, IL2, IL4, IL6, MTTP, TGFB1, TIMP1, ZAP70] |
| GO:0046651 | lymphocyte proliferation | GO_BiologicalProcess-EBI-UniProt-GOA_27.02.2019_00h00 | 3.98E-10 | 1.84E-09 | 6.31E-19 | 1.14E-17 | Group111 | 4.10 | 15 | [BAX, CASP3, CCL5, CDKN2A, CXCL8, HPRT1, IGF1, IL1B, IL2, IL4, IL6, MTTP, TGFB1, TIMP1, ZAP70] |
| GO:0002687 | positive regulation of leukocyte migration | GO_BiologicalProcess-EBI-UniProt-GOA_27.02.2019_00h00 | 4.72E-10 | 2.15E-09 | 3.86E-11 | 9.49E-11 | Group070 | 6.88 | 11 | [APP, CCL5, CXCL8, ELANE, ICAM1, IGF1, IL6, MAPK14, TGFB1, TNF, VEGFA] |
| GO:0051054 | positive regulation of DNA metabolic process | GO_BiologicalProcess-EBI-UniProt-GOA_27.02.2019_00h00 | 5.89E-10 | 2.66E-09 | 5.89E-10 | 1.12E-09 | Group020 | 4.43 | 14 | [AKT1, BAX, CCL2, CDKN1A, CDKN2A, EGFR, FGFR1, HRAS, HSP90AA1, IL2, IL4, IL6, JUN, TGFB1] |
| GO:0014074 | response to purine-containing compound | GO_BiologicalProcess-EBI-UniProt-GOA_27.02.2019_00h00 | 6.17E-10 | 2.76E-09 | 7.42E-11 | 1.70E-10 | Group073 | 5.71 | 12 | [APP, BRAF, CFTR, DHODH, DNTT, FOS, IL1B, JUN, PDE3A, PTEN, PTGS2, REN] |
| GO:0048145 | regulation of fibroblast proliferation | GO_BiologicalProcess-EBI-UniProt-GOA_27.02.2019_00h00 | 6.38E-10 | 2.82E-09 | 6.38E-10 | 1.19E-09 | Group087 | 8.06 | 10 | [BAX, CCNA2, CDK4, CDKN1A, EGFR, FN1, IGF1, JUN, TGFB1, TP53] |
| GO:0042136 | neurotransmitter biosynthetic process | GO_BiologicalProcess-EBI-UniProt-GOA_27.02.2019_00h00 | 6.38E-10 | 2.82E-09 | 8.72E-12 | 2.55E-11 | Group109 | 8.06 | 10 | [ACHE, AKT1, HSP90AA1, ICAM1, IL1B, NOS2, NOS3, PTGS2, SHMT1, TNF] |
| GO:0031099 | regeneration | GO_BiologicalProcess-EBI-UniProt-GOA_27.02.2019_00h00 | 7.40E-10 | 3.24E-09 | 7.40E-10 | 1.33E-09 | Group088 | 4.91 | 13 | [APOA1, BRAF, CCNA2, CDK4, CDKN1A, EGFR, IGF1, IL6, JUN, PTEN, TBXAS1, TGFB1, UGT1A1] |
| GO:0034284 | response to monosaccharide | GO_BiologicalProcess-EBI-UniProt-GOA_27.02.2019_00h00 | 7.40E-10 | 3.24E-09 | 2.35E-11 | 6.03E-11 | Group091 | 4.91 | 13 | [ADRB2, CASP3, CAT, CFTR, HIF1A, HMGCR, ICAM1, IGF1R, PRKACA, PTEN, PTGS2, RAF1, TGFB1] |
| GO:0071456 | cellular response to hypoxia | GO_BiologicalProcess-EBI-UniProt-GOA_27.02.2019_00h00 | 7.40E-10 | 3.24E-09 | 8.09E-14 | 4.16E-13 | Group096 | 4.91 | 13 | [AKT1, CCNA2, EP300, HIF1A, ICAM1, KLK3, MDM2, P4HB, PTEN, PTGS2, TIMP1, TP53, VEGFA] |
| GO:0034764 | positive regulation of transmembrane transport | GO_BiologicalProcess-EBI-UniProt-GOA_27.02.2019_00h00 | 7.75E-10 | 3.36E-09 | 7.75E-10 | 1.37E-09 | Group003 | 4.89 | 13 | [ADRB2, AKT1, BAX, BRAF, CALM1, CALM2, CFTR, F2, GH1, IGF1, INS, MAPK14, MTTP] |
| GO:1901654 | response to ketone | GO_BiologicalProcess-EBI-UniProt-GOA_27.02.2019_00h00 | 8.49E-10 | 3.64E-09 | 8.49E-10 | 1.48E-09 | Group035 | 4.85 | 13 | [AKT1, CALM1, CALM2, CDK4, CDKN1A, CFTR, EGFR, FOS, ICAM1, TBXAS1, TGFB1, TGFB2, TIMP1] |
| GO:1902895 | positive regulation of pri-miRNA transcription by RNA polymerase II | GO_BiologicalProcess-EBI-UniProt-GOA_27.02.2019_00h00 | 8.70E-10 | 3.69E-09 | 8.70E-10 | 1.49E-09 | Group036 | 17.95 | 7 | [FOS, HIF1A, JUN, STAT3, TGFB1, TGFB2, TP53] |
| GO:0007050 | cell cycle arrest | GO_BiologicalProcess-EBI-UniProt-GOA_27.02.2019_00h00 | 8.84E-10 | 3.72E-09 | 5.39E-12 | 1.71E-11 | Group105 | 4.29 | 14 | [BAX, CDK4, CDKN1A, CDKN2A, CHEK2, CXCL8, EP300, HRAS, MDM2, MTTP, PRKACA, TGFB1, TGFB2, TP53] |
| GO:0071478 | cellular response to radiation | GO_BiologicalProcess-EBI-UniProt-GOA_27.02.2019_00h00 | 9.30E-10 | 3.87E-09 | 8.57E-13 | 3.31E-12 | Group076 | 4.81 | 13 | [BAX, CALM1, CALM2, CDKN1A, CHEK2, EP300, HRAS, MAPK14, MDM2, MME, PTGS2, TGFB1, TP53] |
| GO:2000045 | regulation of G1/S transition of mitotic cell cycle | GO_BiologicalProcess-EBI-UniProt-GOA_27.02.2019_00h00 | 9.47E-10 | 3.91E-09 | 5.39E-12 | 1.71E-11 | Group105 | 5.50 | 12 | [AKT1, BAX, CCL2, CDK4, CDKN1A, CDKN2A, CHEK2, EGFR, EP300, MDM2, PTEN, TP53] |
| GO:0002573 | myeloid leukocyte differentiation | GO_BiologicalProcess-EBI-UniProt-GOA_27.02.2019_00h00 | 1.02E-09 | 4.16E-09 | 1.02E-09 | 1.72E-09 | Group025 | 4.78 | 13 | [APP, CDC42, FOS, IGF1, IL4, JUN, KIT, MAPK14, MMP9, PRKACA, TGFB1, TNF, VEGFA] |
| GO:0045927 | positive regulation of growth | GO_BiologicalProcess-EBI-UniProt-GOA_27.02.2019_00h00 | 1.08E-09 | 4.36E-09 | 1.08E-09 | 1.79E-09 | Group068 | 4.23 | 14 | [AKT1, CDC42, CDKN1A, EGFR, F2, FGFR1, FN1, GH1, IGF1, IL2, INS, MAPK14, TGFB2, VEGFA] |
| GO:1903409 | reactive oxygen species biosynthetic process | GO_BiologicalProcess-EBI-UniProt-GOA_27.02.2019_00h00 | 1.10E-09 | 4.39E-09 | 8.72E-12 | 2.55E-11 | Group109 | 7.63 | 10 | [AKT1, HSP90AA1, ICAM1, IL1B, INS, NOS2, NOS3, PTGS2, STAT3, TNF] |
| GO:0046883 | regulation of hormone secretion | GO_BiologicalProcess-EBI-UniProt-GOA_27.02.2019_00h00 | 1.26E-09 | 4.99E-09 | 9.22E-11 | 2.07E-10 | Group103 | 4.18 | 14 | [ADRB2, CCL5, CFTR, CYP19A1, EGFR, FGFR1, HIF1A, HMGCR, IL1B, INS, NOS2, PRKACA, REN, TNF] |
| GO:1900544 | positive regulation of purine nucleotide metabolic process | GO_BiologicalProcess-EBI-UniProt-GOA_27.02.2019_00h00 | 1.36E-09 | 5.34E-09 | 2.33E-10 | 4.94E-10 | Group098 | 12.12 | 8 | [CDKN1A, HIF1A, IGF1, INS, NOS2, NOS3, PTGS2, STAT3] |
| GO:0005125 | cytokine activity | GO_BiologicalProcess-EBI-UniProt-GOA_27.02.2019_00h00 | 1.45E-09 | 5.65E-09 | 1.45E-09 | 2.37E-09 | Group034 | 4.64 | 13 | [CCL2, CCL5, CXCL8, IGF1, IL1B, IL2, IL4, IL6, TGFB1, TGFB2, TIMP1, TNF, VEGFA] |
| GO:0050728 | negative regulation of inflammatory response | GO_BiologicalProcess-EBI-UniProt-GOA_27.02.2019_00h00 | 1.65E-09 | 6.39E-09 | 3.67E-12 | 1.24E-11 | Group067 | 6.11 | 11 | [ADRB2, APOA1, CYP19A1, ELANE, F2, IGF1, IL2, IL4, IL6, INS, LDLR] |
| GO:0032355 | response to estradiol | GO_BiologicalProcess-EBI-UniProt-GOA_27.02.2019_00h00 | 1.97E-09 | 7.54E-09 | 1.97E-09 | 3.17E-09 | Group063 | 6.01 | 11 | [APOB, CASP3, CAT, CCNA2, EGFR, GH1, PTEN, PTGS2, STAT3, TGFB1, UGT1A1] |
| GO:0097746 | regulation of blood vessel diameter | GO_BiologicalProcess-EBI-UniProt-GOA_27.02.2019_00h00 | 2.34E-09 | 8.88E-09 | 1.16E-10 | 2.55E-10 | Group097 | 5.91 | 11 | [ADRB2, AKT1, ECE1, EGFR, HMGCR, ICAM1, INS, NOS3, PTGS2, SOD2, TBXAS1] |
| GO:0048146 | positive regulation of fibroblast proliferation | GO_BiologicalProcess-EBI-UniProt-GOA_27.02.2019_00h00 | 2.46E-09 | 9.18E-09 | 6.38E-10 | 1.19E-09 | Group087 | 11.27 | 8 | [CCNA2, CDK4, CDKN1A, EGFR, FN1, IGF1, JUN, TGFB1] |
| GO:1903708 | positive regulation of hemopoiesis | GO_BiologicalProcess-EBI-UniProt-GOA_27.02.2019_00h00 | 2.45E-09 | 9.22E-09 | 2.45E-09 | 3.89E-09 | Group099 | 5.06 | 12 | [FOS, HIF1A, IGF1, IL2, IL4, JUN, MAPK14, PRKACA, STAT3, TGFB1, TNF, ZAP70] |
| GO:0010821 | regulation of mitochondrion organization | GO_BiologicalProcess-EBI-UniProt-GOA_27.02.2019_00h00 | 2.69E-09 | 9.97E-09 | 2.69E-09 | 4.22E-09 | Group054 | 5.02 | 12 | [AKT1, BAP1, BAX, DHODH, HDAC6, HIF1A, IGF1, IL6, KDR, KLK3, MMP9, TP53] |
| GO:0046890 | regulation of lipid biosynthetic process | GO_BiologicalProcess-EBI-UniProt-GOA_27.02.2019_00h00 | 2.96E-09 | 1.08E-08 | 5.85E-11 | 1.37E-10 | Group102 | 4.98 | 12 | [ADRB2, AKT1, APOA1, APOB, CCNA2, CDK4, HMGCR, IL1B, INS, LDLR, PTGS2, TNF] |
| GO:0097153 | cysteine-type endopeptidase activity involved in apoptotic process | GO_BiologicalProcess-EBI-UniProt-GOA_27.02.2019_00h00 | 2.96E-09 | 1.08E-08 | 2.96E-09 | 4.56E-09 | Group079 | 4.38 | 13 | [AKT1, BAX, CASP3, CCNA2, CDKN2A, IL6, MDM2, MMP9, PTGS2, RAF1, TNF, VEGFA, XIAP] |
| GO:1904705 | regulation of vascular smooth muscle cell proliferation | GO_BiologicalProcess-EBI-UniProt-GOA_27.02.2019_00h00 | 3.08E-09 | 1.11E-08 | 3.08E-09 | 4.69E-09 | Group040 | 10.96 | 8 | [CDKN1A, IGF1, JUN, MDM2, MMP9, PTEN, SOD2, TNF] |
| GO:0046889 | positive regulation of lipid biosynthetic process | GO_BiologicalProcess-EBI-UniProt-GOA_27.02.2019_00h00 | 3.89E-09 | 1.39E-08 | 5.85E-11 | 1.37E-10 | Group102 | 8.26 | 9 | [ADRB2, AKT1, APOA1, CCNA2, IL1B, INS, LDLR, PTGS2, TNF] |
| GO:0042445 | hormone metabolic process | GO_BiologicalProcess-EBI-UniProt-GOA_27.02.2019_00h00 | 4.07E-09 | 1.44E-08 | 4.07E-09 | 6.11E-09 | Group009 | 4.26 | 13 | [ACHE, APOA1, CYP19A1, CYP3A4, ECE1, FGFR1, HIF1A, HSD11B1, IL1B, MME, REN, TNF, UGT1A1] |
| GO:0021782 | glial cell development | GO_BiologicalProcess-EBI-UniProt-GOA_27.02.2019_00h00 | 4.11E-09 | 1.45E-08 | 1.66E-14 | 9.45E-14 | Group110 | 6.67 | 10 | [AKT1, APP, EGFR, HRAS, IL1B, IL6, LDLR, PTEN, TGFB1, TNF] |
| GO:0007569 | cell aging | GO_BiologicalProcess-EBI-UniProt-GOA_27.02.2019_00h00 | 4.39E-09 | 1.53E-08 | 4.39E-09 | 6.49E-09 | Group045 | 6.62 | 10 | [CDKN1A, CDKN2A, CHEK2, HRAS, ICAM1, MAPK14, MME, MTTP, PTEN, TP53] |
| GO:0002262 | myeloid cell homeostasis | GO_BiologicalProcess-EBI-UniProt-GOA_27.02.2019_00h00 | 4.51E-09 | 1.56E-08 | 4.51E-09 | 6.58E-09 | Group004 | 5.56 | 11 | [BAP1, BAX, CASP3, HIF1A, IGF1, IL6, KIT, MAPK14, STAT3, TIMP1, VEGFA] |
| GO:0050679 | positive regulation of epithelial cell proliferation | GO_BiologicalProcess-EBI-UniProt-GOA_27.02.2019_00h00 | 4.90E-09 | 1.68E-08 | 4.48E-11 | 1.08E-10 | Group094 | 4.76 | 12 | [AKT1, EGFR, FGFR1, HIF1A, HRAS, IGF1, JUN, KDR, PRKACA, STAT3, TGFB1, VEGFA] |
| GO:0030072 | peptide hormone secretion | GO_BiologicalProcess-EBI-UniProt-GOA_27.02.2019_00h00 | 5.56E-09 | 1.89E-08 | 9.22E-11 | 2.07E-10 | Group103 | 4.15 | 13 | [ADRB2, CCL5, CFTR, EGFR, HIF1A, HMGCR, IL1B, IL6, INS, NOS2, PRKACA, RAF1, TNF] |
| GO:0002260 | lymphocyte homeostasis | GO_BiologicalProcess-EBI-UniProt-GOA_27.02.2019_00h00 | 6.46E-09 | 2.18E-08 | 1.90E-11 | 5.25E-11 | Group089 | 10.00 | 8 | [AKT1, BAX, CASP3, HIF1A, IL2, KLK3, MTTP, TGFB1] |
| GO:0071156 | regulation of cell cycle arrest | GO_BiologicalProcess-EBI-UniProt-GOA_27.02.2019_00h00 | 6.80E-09 | 2.28E-08 | 5.39E-12 | 1.71E-11 | Group105 | 6.33 | 10 | [BAX, CDK4, CDKN1A, CDKN2A, CHEK2, EP300, MDM2, PRKACA, TGFB1, TP53] |
| GO:2000134 | negative regulation of G1/S transition of mitotic cell cycle | GO_BiologicalProcess-EBI-UniProt-GOA_27.02.2019_00h00 | 6.80E-09 | 2.28E-08 | 5.39E-12 | 1.71E-11 | Group105 | 6.33 | 10 | [BAX, CCL2, CDK4, CDKN1A, CDKN2A, CHEK2, EP300, MDM2, PTEN, TP53] |
| GO:0001938 | positive regulation of endothelial cell proliferation | GO_BiologicalProcess-EBI-UniProt-GOA_27.02.2019_00h00 | 7.86E-09 | 2.61E-08 | 4.48E-11 | 1.08E-10 | Group094 | 7.63 | 9 | [AKT1, FGFR1, HIF1A, HRAS, JUN, KDR, PRKACA, STAT3, VEGFA] |
| GO:0050870 | positive regulation of T cell activation | GO_BiologicalProcess-EBI-UniProt-GOA_27.02.2019_00h00 | 9.36E-09 | 3.09E-08 | 6.31E-19 | 1.14E-17 | Group111 | 4.49 | 12 | [AKT1, CCL2, CCL5, CDC42, IGF1, IL1B, IL2, IL4, IL6, TGFB1, TIMP1, ZAP70] |
| GO:0045766 | positive regulation of angiogenesis | GO_BiologicalProcess-EBI-UniProt-GOA_27.02.2019_00h00 | 1.23E-08 | 4.03E-08 | 2.30E-10 | 4.97E-10 | Group081 | 5.05 | 11 | [CXCL8, HIF1A, IL1B, IL6, KDR, MTTP, NOS3, PRKACA, PTGS2, STAT3, VEGFA] |
| GO:0006809 | nitric oxide biosynthetic process | GO_BiologicalProcess-EBI-UniProt-GOA_27.02.2019_00h00 | 1.27E-08 | 4.11E-08 | 8.72E-12 | 2.55E-11 | Group109 | 9.20 | 8 | [AKT1, HSP90AA1, ICAM1, IL1B, NOS2, NOS3, PTGS2, TNF] |
| GO:0042129 | regulation of T cell proliferation | GO_BiologicalProcess-EBI-UniProt-GOA_27.02.2019_00h00 | 1.35E-08 | 4.36E-08 | 6.31E-19 | 1.14E-17 | Group111 | 5.00 | 11 | [CASP3, CCL5, CDKN2A, IGF1, IL1B, IL2, IL4, IL6, TGFB1, TIMP1, ZAP70] |
| GO:0043542 | endothelial cell migration | GO_BiologicalProcess-EBI-UniProt-GOA_27.02.2019_00h00 | 1.41E-08 | 4.52E-08 | 7.82E-15 | 5.28E-14 | Group108 | 4.33 | 12 | [AKT1, APOA1, FGFR1, HIF1A, KDR, MTTP, NOS3, PRKACA, PTEN, PTGS2, TGFB1, VEGFA] |
| GO:0031100 | animal organ regeneration | GO_BiologicalProcess-EBI-UniProt-GOA_27.02.2019_00h00 | 1.50E-08 | 4.77E-08 | 7.40E-10 | 1.33E-09 | Group088 | 7.09 | 9 | [APOA1, CCNA2, CDK4, CDKN1A, EGFR, IL6, TBXAS1, TGFB1, UGT1A1] |
| GO:0045639 | positive regulation of myeloid cell differentiation | GO_BiologicalProcess-EBI-UniProt-GOA_27.02.2019_00h00 | 1.50E-08 | 4.77E-08 | 2.45E-09 | 3.89E-09 | Group099 | 7.09 | 9 | [FOS, HIF1A, IGF1, JUN, MAPK14, PRKACA, STAT3, TGFB1, TNF] |
| GO:0097192 | extrinsic apoptotic signaling pathway in absence of ligand | GO_BiologicalProcess-EBI-UniProt-GOA_27.02.2019_00h00 | 1.52E-08 | 4.79E-08 | 1.52E-08 | 2.19E-08 | Group027 | 8.99 | 8 | [AKT1, BAX, CASP3, FGFR1, IGF1, IL1B, IL2, TNF] |
| GO:2001242 | regulation of intrinsic apoptotic signaling pathway | GO_BiologicalProcess-EBI-UniProt-GOA_27.02.2019_00h00 | 1.63E-08 | 5.10E-08 | 7.37E-13 | 2.95E-12 | Group101 | 4.91 | 11 | [AKT1, BAX, HIF1A, INS, MDM2, MMP9, P4HB, PTGS2, SOD2, TIMP1, TP53] |
| GO:0050806 | positive regulation of synaptic transmission | GO_BiologicalProcess-EBI-UniProt-GOA_27.02.2019_00h00 | 1.71E-08 | 5.31E-08 | 1.71E-08 | 2.43E-08 | Group018 | 4.89 | 11 | [ADRB2, APP, BRAF, CCL2, EGFR, INS, MME, MTTP, PTEN, PTGS2, TNF] |
| GO:0046686 | response to cadmium ion | GO_BiologicalProcess-EBI-UniProt-GOA_27.02.2019_00h00 | 1.98E-08 | 6.10E-08 | 1.98E-08 | 2.77E-08 | Group030 | 8.70 | 8 | [AKT1, CAT, CDKN1A, EGFR, FOS, JUN, KIT, MMP9] |
| GO:0014002 | astrocyte development | GO_BiologicalProcess-EBI-UniProt-GOA_27.02.2019_00h00 | 2.01E-08 | 6.14E-08 | 1.66E-14 | 9.45E-14 | Group110 | 11.67 | 7 | [APP, EGFR, HRAS, IL1B, IL6, LDLR, TNF] |
| GO:0060191 | regulation of lipase activity | GO_BiologicalProcess-EBI-UniProt-GOA_27.02.2019_00h00 | 2.25E-08 | 6.85E-08 | 2.25E-08 | 3.12E-08 | Group080 | 6.77 | 9 | [APOA1, CCL5, CCNA2, EGFR, FGFR1, HRAS, KIT, MTTP, SELE] |
| GO:0010212 | response to ionizing radiation | GO_BiologicalProcess-EBI-UniProt-GOA_27.02.2019_00h00 | 3.41E-08 | 1.03E-07 | 3.41E-08 | 4.60E-08 | Group053 | 5.35 | 10 | [BAX, CASP3, CDKN1A, CHEK2, HRAS, ICAM1, MAPK14, MDM2, TGFB1, TP53] |
| GO:0061900 | glial cell activation | GO_BiologicalProcess-EBI-UniProt-GOA_27.02.2019_00h00 | 3.54E-08 | 1.06E-07 | 1.66E-14 | 9.45E-14 | Group110 | 10.77 | 7 | [APP, EGFR, IL1B, IL6, JUN, LDLR, TNF] |
| GO:0043271 | negative regulation of ion transport | GO_BiologicalProcess-EBI-UniProt-GOA_27.02.2019_00h00 | 3.59E-08 | 1.07E-07 | 7.26E-10 | 1.33E-09 | Group093 | 5.32 | 10 | [AKT1, CALM1, CALM2, ICAM1, MMP9, NOS3, PTEN, PTGS2, TGFB1, TIMP1] |
| GO:0030235 | nitric-oxide synthase regulator activity | GO_BiologicalProcess-EBI-UniProt-GOA_27.02.2019_00h00 | 4.21E-08 | 1.25E-07 | 4.21E-08 | 5.61E-08 | Group058 | 25.00 | 5 | [AKT1, CALM1, CALM2, EGFR, HSP90AA1] |
| GO:0048469 | cell maturation | GO_BiologicalProcess-EBI-UniProt-GOA_27.02.2019_00h00 | 4.45E-08 | 1.31E-07 | 4.45E-08 | 5.86E-08 | Group014 | 4.45 | 11 | [APP, CDKN1A, CFTR, FGFR1, HIF1A, PDE3A, PRKACA, REN, TBXAS1, TIMP1, VEGFA] |
| GO:0002367 | cytokine production involved in immune response | GO_BiologicalProcess-EBI-UniProt-GOA_27.02.2019_00h00 | 4.49E-08 | 1.31E-07 | 4.49E-08 | 5.85E-08 | Group008 | 6.25 | 9 | [APOA1, IL1B, IL4, IL6, KIT, MAPK14, TGFB1, TGFB2, TNF] |
| GO:1903426 | regulation of reactive oxygen species biosynthetic process | GO_BiologicalProcess-EBI-UniProt-GOA_27.02.2019_00h00 | 4.83E-08 | 1.40E-07 | 8.72E-12 | 2.55E-11 | Group109 | 7.77 | 8 | [AKT1, HSP90AA1, ICAM1, IL1B, INS, PTGS2, STAT3, TNF] |
| GO:0010827 | regulation of glucose transmembrane transport | GO_BiologicalProcess-EBI-UniProt-GOA_27.02.2019_00h00 | 6.05E-08 | 1.73E-07 | 6.05E-08 | 7.68E-08 | Group055 | 7.55 | 8 | [AKT1, BRAF, GH1, IGF1, IL1B, INS, MAPK14, TNF] |
| GO:0019218 | regulation of steroid metabolic process | GO_BiologicalProcess-EBI-UniProt-GOA_27.02.2019_00h00 | 6.04E-08 | 1.74E-07 | 6.04E-08 | 7.76E-08 | Group057 | 6.04 | 9 | [ADRB2, APOA1, APOB, HMGCR, IL1B, KIT, LDLR, TNF, UGT1A1] |
| GO:0034763 | negative regulation of transmembrane transport | GO_BiologicalProcess-EBI-UniProt-GOA_27.02.2019_00h00 | 6.40E-08 | 1.82E-07 | 7.26E-10 | 1.33E-09 | Group093 | 6.00 | 9 | [AKT1, CALM1, CALM2, IL1B, MMP9, PTEN, TGFB1, TIMP1, TNF] |
| GO:0035234 | ectopic germ cell programmed cell death | GO_BiologicalProcess-EBI-UniProt-GOA_27.02.2019_00h00 | 7.41E-08 | 2.09E-07 | 7.41E-08 | 9.30E-08 | Group005 | 44.44 | 4 | [BAX, IGF1, IL1B, KIT] |
| GO:0090316 | positive regulation of intracellular protein transport | GO_BiologicalProcess-EBI-UniProt-GOA_27.02.2019_00h00 | 8.11E-08 | 2.26E-07 | 8.11E-08 | 1.01E-07 | Group078 | 4.88 | 10 | [BAP1, HRAS, IL1B, KLK3, MAPK14, MDM2, PRKACA, PTGS2, TGFB1, TP53] |
| GO:0090276 | regulation of peptide hormone secretion | GO_BiologicalProcess-EBI-UniProt-GOA_27.02.2019_00h00 | 8.11E-08 | 2.28E-07 | 9.22E-11 | 2.07E-10 | Group103 | 4.20 | 11 | [ADRB2, CCL5, CFTR, EGFR, HIF1A, HMGCR, IL1B, INS, NOS2, PRKACA, TNF] |
| GO:0007435 | salivary gland morphogenesis | GO_BiologicalProcess-EBI-UniProt-GOA_27.02.2019_00h00 | 9.54E-08 | 2.64E-07 | 9.54E-08 | 1.17E-07 | Group044 | 13.33 | 6 | [EGFR, FGFR1, IL6, TGFB1, TGFB2, TNF] |
| GO:1904035 | regulation of epithelial cell apoptotic process | GO_BiologicalProcess-EBI-UniProt-GOA_27.02.2019_00h00 | 9.97E-08 | 2.74E-07 | 9.97E-08 | 1.21E-07 | Group039 | 7.08 | 8 | [BRAF, CCL2, ICAM1, IL4, IL6, KDR, MTTP, TNF] |
| GO:0009612 | response to mechanical stimulus | GO_BiologicalProcess-EBI-UniProt-GOA_27.02.2019_00h00 | 1.06E-07 | 2.90E-07 | 1.06E-07 | 1.27E-07 | Group050 | 4.09 | 11 | [AKT1, EGFR, ELANE, FOS, IL1B, JUN, KIT, MAPK14, PTGS2, RAF1, TGFB1] |
| GO:0043154 | negative regulation of cysteine-type endopeptidase activity involved in apoptotic process | GO_BiologicalProcess-EBI-UniProt-GOA_27.02.2019_00h00 | 1.07E-07 | 2.90E-07 | 2.96E-09 | 4.56E-09 | Group079 | 7.02 | 8 | [AKT1, IL6, MDM2, MMP9, PTGS2, RAF1, VEGFA, XIAP] |
| GO:0030307 | positive regulation of cell growth | GO_BiologicalProcess-EBI-UniProt-GOA_27.02.2019_00h00 | 1.16E-07 | 3.13E-07 | 1.08E-09 | 1.79E-09 | Group068 | 4.69 | 10 | [AKT1, CDC42, EGFR, F2, FN1, IGF1, IL2, INS, TGFB2, VEGFA] |
| GO:0019207 | kinase regulator activity | GO_BiologicalProcess-EBI-UniProt-GOA_27.02.2019_00h00 | 1.23E-07 | 3.30E-07 | 1.23E-07 | 1.46E-07 | Group056 | 4.03 | 11 | [CALM1, CALM2, CASP3, CCL5, CCNA2, CDK4, CDKN1A, CDKN2A, IL2, TGFB1, TIMP1] |
| GO:0050920 | regulation of chemotaxis | GO_BiologicalProcess-EBI-UniProt-GOA_27.02.2019_00h00 | 1.28E-07 | 3.40E-07 | 1.28E-07 | 1.50E-07 | Group019 | 4.01 | 11 | [CCL2, CCL5, CXCL8, CYP19A1, ELANE, FGFR1, IL6, KDR, MAPK14, TGFB1, VEGFA] |
| GO:0070542 | response to fatty acid | GO_BiologicalProcess-EBI-UniProt-GOA_27.02.2019_00h00 | 1.31E-07 | 3.46E-07 | 1.31E-07 | 1.52E-07 | Group023 | 6.84 | 8 | [ADRB2, AKT1, APOB, CAT, CDK4, LDLR, PTGS2, TBXAS1] |
| GO:1901216 | positive regulation of neuron death | GO_BiologicalProcess-EBI-UniProt-GOA_27.02.2019_00h00 | 1.31E-07 | 3.46E-07 | 1.31E-07 | 1.52E-07 | Group033 | 6.84 | 8 | [BAX, CALM2, CASP3, FOS, JUN, MTTP, TGFB2, TNF] |
| GO:0097756 | negative regulation of blood vessel diameter | GO_BiologicalProcess-EBI-UniProt-GOA_27.02.2019_00h00 | 1.31E-07 | 3.46E-07 | 1.16E-10 | 2.55E-10 | Group097 | 6.84 | 8 | [AKT1, ECE1, EGFR, HMGCR, ICAM1, INS, PTGS2, TBXAS1] |
| GO:2001236 | regulation of extrinsic apoptotic signaling pathway | GO_BiologicalProcess-EBI-UniProt-GOA_27.02.2019_00h00 | 1.32E-07 | 3.48E-07 | 2.13E-11 | 5.76E-11 | Group083 | 4.63 | 10 | [AKT1, FGFR1, ICAM1, IGF1, IL1B, IL6, NOS3, PTEN, RAF1, TNF] |
| GO:0051702 | interaction with symbiont | GO_BiologicalProcess-EBI-UniProt-GOA_27.02.2019_00h00 | 1.40E-07 | 3.65E-07 | 2.34E-08 | 3.20E-08 | Group071 | 6.78 | 8 | [CALM1, CALM2, CCL5, ELANE, EP300, F2, FN1, JUN] |
| GO:0045429 | positive regulation of nitric oxide biosynthetic process | GO_BiologicalProcess-EBI-UniProt-GOA_27.02.2019_00h00 | 1.42E-07 | 3.67E-07 | 8.72E-12 | 2.55E-11 | Group109 | 12.50 | 6 | [AKT1, HSP90AA1, ICAM1, IL1B, PTGS2, TNF] |
| GO:0090399 | replicative senescence | GO_BiologicalProcess-EBI-UniProt-GOA_27.02.2019_00h00 | 1.41E-07 | 3.68E-07 | 1.41E-07 | 1.63E-07 | Group026 | 20.00 | 5 | [CDKN1A, CDKN2A, CHEK2, MME, TP53] |
| GO:0016125 | sterol metabolic process | GO_BiologicalProcess-EBI-UniProt-GOA_27.02.2019_00h00 | 1.44E-07 | 3.71E-07 | 7.11E-14 | 3.84E-13 | Group104 | 4.59 | 10 | [ADRB2, APOA1, APOB, APP, CAT, CFTR, CYP19A1, HMGCR, IL4, LDLR] |
| GO:0008585 | female gonad development | GO_BiologicalProcess-EBI-UniProt-GOA_27.02.2019_00h00 | 1.49E-07 | 3.81E-07 | 1.49E-07 | 1.69E-07 | Group047 | 6.72 | 8 | [BAX, CASP3, CYP19A1, ICAM1, IGF1, KIT, NOS3, VEGFA] |
| GO:0051926 | negative regulation of calcium ion transport | GO_BiologicalProcess-EBI-UniProt-GOA_27.02.2019_00h00 | 1.52E-07 | 3.86E-07 | 7.26E-10 | 1.33E-09 | Group093 | 8.75 | 7 | [CALM1, CALM2, ICAM1, NOS3, PTGS2, TGFB1, TIMP1] |
| GO:0048565 | digestive tract development | GO_BiologicalProcess-EBI-UniProt-GOA_27.02.2019_00h00 | 1.61E-07 | 4.06E-07 | 1.61E-07 | 1.81E-07 | Group015 | 5.39 | 9 | [CDKN1A, CXCL8, EGFR, HIF1A, KIT, TBXAS1, TGFB1, TGFB2, TNF] |
| GO:0001659 | temperature homeostasis | GO_BiologicalProcess-EBI-UniProt-GOA_27.02.2019_00h00 | 1.71E-07 | 4.29E-07 | 1.71E-07 | 1.90E-07 | Group000 | 4.50 | 10 | [ACHE, ADRB2, IGF1R, IL1B, IL4, LDLR, PTGS2, STAT3, TNF, VEGFA] |
| GO:0051897 | positive regulation of protein kinase B signaling | GO_BiologicalProcess-EBI-UniProt-GOA_27.02.2019_00h00 | 1.78E-07 | 4.42E-07 | 1.24E-11 | 3.53E-11 | Group072 | 4.48 | 10 | [EGFR, FGFR1, HSP90AA1, IGF1, IGF1R, IL6, INS, KIT, TGFB1, TNF] |
| GO:1900407 | regulation of cellular response to oxidative stress | GO_BiologicalProcess-EBI-UniProt-GOA_27.02.2019_00h00 | 1.81E-07 | 4.43E-07 | 1.81E-07 | 1.97E-07 | Group031 | 6.56 | 8 | [AKT1, HDAC6, HIF1A, IL6, INS, P4HB, SOD2, TNF] |
| GO:0032642 | regulation of chemokine production | GO_BiologicalProcess-EBI-UniProt-GOA_27.02.2019_00h00 | 1.81E-07 | 4.43E-07 | 1.81E-07 | 1.97E-07 | Group065 | 6.56 | 8 | [ADRB2, APP, ELANE, HIF1A, HSP90AA1, IL1B, IL6, TNF] |
| GO:0008637 | apoptotic mitochondrial changes | GO_BiologicalProcess-EBI-UniProt-GOA_27.02.2019_00h00 | 1.78E-07 | 4.44E-07 | 1.78E-07 | 1.96E-07 | Group048 | 5.33 | 9 | [AKT1, BAX, CDKN2A, IGF1, IL6, JUN, MMP9, SOD2, TP53] |
| GO:0006977 | DNA damage response, signal transduction by p53 class mediator resulting in cell cycle arrest | GO_BiologicalProcess-EBI-UniProt-GOA_27.02.2019_00h00 | 1.80E-07 | 4.45E-07 | 5.39E-12 | 1.71E-11 | Group105 | 8.54 | 7 | [BAX, CDKN1A, CDKN2A, CHEK2, EP300, MDM2, TP53] |
| GO:0035821 | modification of morphology or physiology of other organism | GO_BiologicalProcess-EBI-UniProt-GOA_27.02.2019_00h00 | 2.19E-07 | 5.30E-07 | 2.34E-08 | 3.20E-08 | Group071 | 4.39 | 10 | [CALM1, CALM2, CCL5, ELANE, EP300, F2, JUN, NOS2, TBXAS1, TGFB1] |
| GO:2000106 | regulation of leukocyte apoptotic process | GO_BiologicalProcess-EBI-UniProt-GOA_27.02.2019_00h00 | 2.18E-07 | 5.32E-07 | 3.60E-10 | 7.06E-10 | Group082 | 6.40 | 8 | [BAX, CCL5, CDKN2A, HIF1A, IGF1, IL2, PTEN, TP53] |
| GO:1903580 | positive regulation of ATP metabolic process | GO_BiologicalProcess-EBI-UniProt-GOA_27.02.2019_00h00 | 2.32E-07 | 5.59E-07 | 2.33E-10 | 4.94E-10 | Group098 | 11.54 | 6 | [CDKN1A, HIF1A, IGF1, INS, PTGS2, STAT3] |
| GO:0043112 | receptor metabolic process | GO_BiologicalProcess-EBI-UniProt-GOA_27.02.2019_00h00 | 2.78E-07 | 6.63E-07 | 2.78E-07 | 2.98E-07 | Group010 | 4.27 | 10 | [ACHE, CALM2, CXCL8, ECE1, HDAC6, HIF1A, SELE, TGFB1, TNF, VEGFA] |
| GO:0030278 | regulation of ossification | GO_BiologicalProcess-EBI-UniProt-GOA_27.02.2019_00h00 | 2.78E-07 | 6.63E-07 | 2.78E-07 | 2.98E-07 | Group059 | 4.27 | 10 | [ADRB2, CCNA2, HIF1A, IGF1, IL6, MAPK14, PRKACA, TGFB1, TGFB2, TNF] |
| GO:0048010 | vascular endothelial growth factor receptor signaling pathway | GO_BiologicalProcess-EBI-UniProt-GOA_27.02.2019_00h00 | 2.78E-07 | 6.67E-07 | 2.78E-07 | 3.00E-07 | Group051 | 6.20 | 8 | [CDC42, CXCL8, HIF1A, HSP90AA1, IL1B, KDR, MAPK14, VEGFA] |
| GO:0070640 | vitamin D3 metabolic process | GO_BiologicalProcess-EBI-UniProt-GOA_27.02.2019_00h00 | 2.88E-07 | 6.82E-07 | 2.88E-07 | 3.04E-07 | Group024 | 33.33 | 4 | [CYP3A4, FGFR1, IL1B, TNF] |
| GO:1903034 | regulation of response to wounding | GO_BiologicalProcess-EBI-UniProt-GOA_27.02.2019_00h00 | 2.89E-07 | 6.82E-07 | 2.89E-07 | 3.04E-07 | Group037 | 4.26 | 10 | [BRAF, CDKN1A, F2, GP1BA, HMGCR, HRAS, KLK3, NOS3, PRKACA, PTEN] |
| GO:0042102 | positive regulation of T cell proliferation | GO_BiologicalProcess-EBI-UniProt-GOA_27.02.2019_00h00 | 2.95E-07 | 6.92E-07 | 6.31E-19 | 1.14E-17 | Group111 | 6.15 | 8 | [CCL5, IGF1, IL1B, IL2, IL4, IL6, TIMP1, ZAP70] |
| GO:0048167 | regulation of synaptic plasticity | GO_BiologicalProcess-EBI-UniProt-GOA_27.02.2019_00h00 | 3.25E-07 | 7.59E-07 | 3.25E-07 | 3.38E-07 | Group011 | 4.20 | 10 | [APP, BRAF, CALM1, HRAS, INS, KIT, MME, MTTP, PTEN, PTGS2] |
| GO:2001243 | negative regulation of intrinsic apoptotic signaling pathway | GO_BiologicalProcess-EBI-UniProt-GOA_27.02.2019_00h00 | 3.52E-07 | 8.11E-07 | 7.37E-13 | 2.95E-12 | Group101 | 6.02 | 8 | [AKT1, HIF1A, INS, MDM2, MMP9, PTGS2, SOD2, TIMP1] |
| GO:0045471 | response to ethanol | GO_BiologicalProcess-EBI-UniProt-GOA_27.02.2019_00h00 | 3.50E-07 | 8.12E-07 | 3.50E-07 | 3.60E-07 | Group013 | 4.92 | 9 | [CAT, CDKN1A, HMGCR, ICAM1, IL2, PTEN, STAT3, TBXAS1, UGT1A1] |
| GO:0045740 | positive regulation of DNA replication | GO_BiologicalProcess-EBI-UniProt-GOA_27.02.2019_00h00 | 3.64E-07 | 8.34E-07 | 3.64E-07 | 3.71E-07 | Group001 | 10.71 | 6 | [CDKN1A, CDKN2A, EGFR, FGFR1, HRAS, JUN] |
| GO:1900274 | regulation of phospholipase C activity | GO_BiologicalProcess-EBI-UniProt-GOA_27.02.2019_00h00 | 3.64E-07 | 8.34E-07 | 2.25E-08 | 3.12E-08 | Group080 | 10.71 | 6 | [EGFR, FGFR1, HRAS, KIT, MTTP, SELE] |
| GO:1902107 | positive regulation of leukocyte differentiation | GO_BiologicalProcess-EBI-UniProt-GOA_27.02.2019_00h00 | 3.67E-07 | 8.36E-07 | 2.45E-09 | 3.89E-09 | Group099 | 4.89 | 9 | [FOS, IGF1, IL2, IL4, JUN, PRKACA, TGFB1, TNF, ZAP70] |
| GO:0002673 | regulation of acute inflammatory response | GO_BiologicalProcess-EBI-UniProt-GOA_27.02.2019_00h00 | 4.05E-07 | 9.19E-07 | 4.05E-07 | 4.09E-07 | Group002 | 10.53 | 6 | [IL1B, IL6, INS, KLK3, PTGS2, TNF] |
| GO:0038083 | peptidyl-tyrosine autophosphorylation | GO_BiologicalProcess-EBI-UniProt-GOA_27.02.2019_00h00 | 4.05E-07 | 9.19E-07 | 4.05E-07 | 4.09E-07 | Group007 | 10.53 | 6 | [EGFR, IGF1R, KDR, MME, MTTP, VEGFA] |
| GO:0006694 | steroid biosynthetic process | GO_BiologicalProcess-EBI-UniProt-GOA_27.02.2019_00h00 | 4.09E-07 | 9.24E-07 | 4.09E-07 | 4.09E-07 | Group042 | 4.10 | 10 | [ADRB2, APOA1, APOB, CFTR, CYP19A1, CYP3A4, HMGCR, HSD11B1, IL1B, TNF] |
| GO:0046822 | regulation of nucleocytoplasmic transport | GO_BiologicalProcess-EBI-UniProt-GOA_27.02.2019_00h00 | 4.41E-07 | 9.91E-07 | 8.11E-08 | 1.01E-07 | Group078 | 5.84 | 8 | [HIF1A, IL1B, MAPK14, MDM2, PRKACA, PTGS2, TGFB1, TP53] |

**Table S5** The data of GO (molecular function) enrichment analysis of GATE candidate targets on NS treatment.

| GOID | GOTerm | Ontology Source | Term PValue | Term PValue Corrected with Benjamini-Hochberg | Group PValue | Group PValue Corrected with Benjamini-Hochberg | GOLevels | GOGroups | % Associated Genes | Nr. Genes | Associated Genes Found |
| --- | --- | --- | --- | --- | --- | --- | --- | --- | --- | --- | --- |
| GO:0033674 | positive regulation of kinase activity | GO_MolecularFunction-EBI-UniProt-GOA_27.02.2019_00h00 | 3.22E-21 | 1.55E-19 | 3.22E-21 | 3.22E-20 | [4, 5, 6] | Group16 | 4.13 | 32 | [ADRB2, AKT1, BRAF, CALM1, CALM2, CCL5, CCNA2, CDKN1A, EGFR, ELANE, F2, FGFR1, GH1, HRAS, HSP90AA1, IGF1, IGF1R, IL1B, IL2, IL4, IL6, INS, KIT, MAPK14, MTTP, PRKACA, RAF1, TGFB1, TGFB2, TIMP1, TNF, VEGFA] |
| GO:0045860 | positive regulation of protein kinase activity | GO_MolecularFunction-EBI-UniProt-GOA_27.02.2019_00h00 | 5.97E-20 | 1.43E-18 | 3.22E-21 | 3.22E-20 | [4, 5, 6, 7] | Group16 | 4.17 | 30 | [ADRB2, AKT1, BRAF, CALM1, CALM2, CCL5, CCNA2, CDKN1A, EGFR, ELANE, FGFR1, GH1, HRAS, HSP90AA1, IGF1, IGF1R, IL1B, IL4, IL6, INS, KIT, MAPK14, MTTP, PRKACA, RAF1, TGFB1, TGFB2, TIMP1, TNF, VEGFA] |
| GO:0032147 | activation of protein kinase activity | GO_MolecularFunction-EBI-UniProt-GOA_27.02.2019_00h00 | 2.40E-18 | 3.84E-17 | 3.22E-21 | 3.22E-20 | [5, 6, 7, 8] | Group16 | 4.95 | 25 | [ADRB2, AKT1, BRAF, CALM1, CALM2, CCL5, CDKN1A, EGFR, GH1, IGF1, IGF1R, IL1B, IL4, IL6, INS, KIT, MAPK14, MTTP, PRKACA, RAF1, TGFB1, TGFB2, TIMP1, TNF, VEGFA] |
| GO:0004517 | nitric-oxide synthase activity | GO_MolecularFunction-EBI-UniProt-GOA_27.02.2019_00h00 | 5.83E-15 | 6.99E-14 | 1.51E-15 | 1.01E-14 | [5] | Group17 | 15.00 | 12 | [AKT1, CALM1, CALM2, EGFR, HIF1A, HRAS, HSP90AA1, IL1B, INS, NOS2, NOS3, TNF] |
| GO:0071902 | positive regulation of protein serine/threonine kinase activity | GO_MolecularFunction-EBI-UniProt-GOA_27.02.2019_00h00 | 1.25E-14 | 1.20E-13 | 3.22E-21 | 3.22E-20 | [5, 6, 7, 8] | Group16 | 4.50 | 21 | [ADRB2, AKT1, BRAF, CALM1, CALM2, CDKN1A, EGFR, ELANE, FGFR1, GH1, HRAS, IGF1, IGF1R, IL1B, IL6, KIT, MAPK14, RAF1, TGFB1, TNF, VEGFA] |
| GO:0016705 | oxidoreductase activity, acting on paired donors, with incorporation or reduction of molecular oxygen | GO_MolecularFunction-EBI-UniProt-GOA_27.02.2019_00h00 | 2.59E-14 | 2.07E-13 | 1.51E-15 | 1.01E-14 | [3] | Group17 | 6.27 | 17 | [AKT1, CALM1, CALM2, CYP19A1, CYP3A4, EGFR, HIF1A, HRAS, HSP90AA1, IL1B, INS, NOS2, NOS3, P4HB, PTGS2, TBXAS1, TNF] |
| GO:0004497 | monooxygenase activity | GO_MolecularFunction-EBI-UniProt-GOA_27.02.2019_00h00 | 6.24E-14 | 4.28E-13 | 1.51E-15 | 1.01E-14 | [3] | Group17 | 7.58 | 15 | [AKT1, CALM1, CALM2, CYP19A1, CYP3A4, EGFR, HIF1A, HRAS, HSP90AA1, IL1B, INS, NOS2, NOS3, TBXAS1, TNF] |
| GO:0051341 | regulation of oxidoreductase activity | GO_MolecularFunction-EBI-UniProt-GOA_27.02.2019_00h00 | 9.11E-14 | 5.47E-13 | 1.51E-15 | 1.01E-14 | [3] | Group17 | 10.00 | 13 | [AKT1, CALM1, CALM2, EGFR, HDAC6, HIF1A, HRAS, HSP90AA1, IL1B, INS, MTTP, NOS3, TNF] |
| GO:0043406 | positive regulation of MAP kinase activity | GO_MolecularFunction-EBI-UniProt-GOA_27.02.2019_00h00 | 4.38E-12 | 2.33E-11 | 1.59E-13 | 7.94E-13 | [6, 7, 8, 9] | Group13 | 4.57 | 17 | [BRAF, CDKN1A, EGFR, ELANE, FGFR1, GH1, HRAS, IGF1, IGF1R, IL1B, IL6, KIT, MAPK14, RAF1, TGFB1, TNF, VEGFA] |
| GO:0005126 | cytokine receptor binding | GO_MolecularFunction-EBI-UniProt-GOA_27.02.2019_00h00 | 4.21E-11 | 2.02E-10 | 7.08E-10 | 1.77E-09 | [4] | Group09 | 4.35 | 16 | [CASP3, CCL2, CCL5, CXCL8, GH1, IGF1, IL1B, IL2, IL4, IL6, STAT3, TGFB1, TGFB2, TIMP1, TNF, VEGFA] |
| GO:0051353 | positive regulation of oxidoreductase activity | GO_MolecularFunction-EBI-UniProt-GOA_27.02.2019_00h00 | 1.35E-10 | 5.88E-10 | 1.51E-15 | 1.01E-14 | [3, 4] | Group17 | 12.00 | 9 | [AKT1, CALM1, CALM2, HIF1A, HRAS, IL1B, INS, MTTP, TNF] |
| GO:0051091 | positive regulation of DNA-binding transcription factor activity | GO_MolecularFunction-EBI-UniProt-GOA_27.02.2019_00h00 | 3.04E-10 | 1.22E-09 | 3.04E-10 | 1.01E-09 | [2, 3, 4] | Group10 | 4.19 | 15 | [AKT1, APP, CAT, EP300, HRAS, ICAM1, IL1B, IL6, INS, KIT, PTEN, STAT3, TGFB1, TNF, VEGFA] |
| GO:0005125 | cytokine activity | GO_MolecularFunction-EBI-UniProt-GOA_27.02.2019_00h00 | 1.49E-09 | 5.51E-09 | 7.08E-10 | 1.77E-09 | [4, 5, 6] | Group09 | 4.64 | 13 | [CCL2, CCL5, CXCL8, IGF1, IL1B, IL2, IL4, IL6, TGFB1, TGFB2, TIMP1, TNF, VEGFA] |
| GO:0097153 | cysteine-type endopeptidase activity involved in apoptotic process | GO_MolecularFunction-EBI-UniProt-GOA_27.02.2019_00h00 | 3.05E-09 | 1.05E-08 | 3.05E-09 | 6.77E-09 | [7] | Group11 | 4.38 | 13 | [AKT1, BAX, CASP3, CCNA2, CDKN2A, IL6, MDM2, MMP9, PTGS2, RAF1, TNF, VEGFA, XIAP] |
| GO:0060191 | regulation of lipase activity | GO_MolecularFunction-EBI-UniProt-GOA_27.02.2019_00h00 | 2.30E-08 | 7.36E-08 | 6.56E-09 | 1.31E-08 | [4, 5] | Group14 | 6.77 | 9 | [APOA1, CCL5, CCNA2, EGFR, FGFR1, HRAS, KIT, MTTP, SELE] |
| GO:0008081 | phosphoric diester hydrolase activity | GO_MolecularFunction-EBI-UniProt-GOA_27.02.2019_00h00 | 2.70E-08 | 8.10E-08 | 6.56E-09 | 1.31E-08 | [5] | Group14 | 5.49 | 10 | [CALM1, CALM2, CCL5, EGFR, FGFR1, HRAS, KIT, MTTP, PDE3A, SELE] |
| GO:0030235 | nitric-oxide synthase regulator activity | GO_MolecularFunction-EBI-UniProt-GOA_27.02.2019_00h00 | 4.26E-08 | 1.20E-07 | 4.85E-10 | 1.39E-09 | [3, 4, 6] | Group12 | 25.00 | 5 | [AKT1, CALM1, CALM2, EGFR, HSP90AA1] |
| GO:0043154 | negative regulation of cysteine-type endopeptidase activity involved in apoptotic process | GO_MolecularFunction-EBI-UniProt-GOA_27.02.2019_00h00 | 1.09E-07 | 2.90E-07 | 3.05E-09 | 6.77E-09 | [7, 8, 9] | Group11 | 7.02 | 8 | [AKT1, IL6, MDM2, MMP9, PTGS2, RAF1, VEGFA, XIAP] |
| GO:0019207 | kinase regulator activity | GO_MolecularFunction-EBI-UniProt-GOA_27.02.2019_00h00 | 1.26E-07 | 3.19E-07 | 1.29E-12 | 5.16E-12 | [3, 4, 5, 6] | Group18 | 4.03 | 11 | [CALM1, CALM2, CASP3, CCL5, CCNA2, CDK4, CDKN1A, CDKN2A, IL2, TGFB1, TIMP1] |
| GO:0004629 | phospholipase C activity | GO_MolecularFunction-EBI-UniProt-GOA_27.02.2019_00h00 | 2.00E-07 | 4.79E-07 | 6.56E-09 | 1.31E-08 | [6] | Group14 | 8.43 | 7 | [CCL5, EGFR, FGFR1, HRAS, KIT, MTTP, SELE] |
| GO:0000079 | regulation of cyclin-dependent protein serine/threonine kinase activity | GO_MolecularFunction-EBI-UniProt-GOA_27.02.2019_00h00 | 5.61E-07 | 1.28E-06 | 4.85E-10 | 1.39E-09 | [4, 6, 7, 8] | Group12 | 5.67 | 8 | [AKT1, CASP3, CCNA2, CDK4, CDKN1A, CDKN2A, EGFR, PTEN] |
| GO:0051092 | positive regulation of NF-kappaB transcription factor activity | GO_MolecularFunction-EBI-UniProt-GOA_27.02.2019_00h00 | 1.38E-06 | 3.01E-06 | 3.04E-10 | 1.01E-09 | [3, 4, 5] | Group10 | 4.19 | 9 | [APP, CAT, HRAS, ICAM1, IL1B, INS, STAT3, TGFB1, TNF] |
| GO:0004712 | protein serine/threonine/tyrosine kinase activity | GO_MolecularFunction-EBI-UniProt-GOA_27.02.2019_00h00 | 1.93E-06 | 3.85E-06 | 1.59E-13 | 7.94E-13 | [4, 6] | Group13 | 4.82 | 8 | [AKT1, BRAF, EGFR, IGF1R, MAPK14, PRKACA, RAF1, TNF] |
| GO:0051101 | regulation of DNA binding | GO_MolecularFunction-EBI-UniProt-GOA_27.02.2019_00h00 | 1.93E-06 | 3.85E-06 | 1.29E-12 | 5.16E-12 | [3, 5] | Group18 | 4.82 | 8 | [CALM1, CALM2, EP300, HDAC8, IGF1, JUN, MMP9, TGFB1] |
| GO:0019209 | kinase activator activity | GO_MolecularFunction-EBI-UniProt-GOA_27.02.2019_00h00 | 1.85E-06 | 3.86E-06 | 1.29E-12 | 5.16E-12 | [4, 5, 6, 7] | Group18 | 6.09 | 7 | [CALM1, CALM2, CCL5, CDKN1A, IL2, TGFB1, TIMP1] |
| GO:0020037 | heme binding | GO_MolecularFunction-EBI-UniProt-GOA_27.02.2019_00h00 | 3.24E-06 | 6.23E-06 | 3.24E-06 | 5.41E-06 | [3, 4] | Group05 | 4.49 | 8 | [CAT, CYP19A1, CYP3A4, NOS2, NOS3, PTGS2, TBXAS1, TIMP1] |
| GO:0004714 | transmembrane receptor protein tyrosine kinase activity | GO_MolecularFunction-EBI-UniProt-GOA_27.02.2019_00h00 | 3.59E-06 | 6.63E-06 | 3.59E-06 | 5.53E-06 | [5, 7] | Group03 | 5.51 | 7 | [APP, EGFR, FGFR1, IGF1R, KDR, KIT, MME] |
| GO:0071901 | negative regulation of protein serine/threonine kinase activity | GO_MolecularFunction-EBI-UniProt-GOA_27.02.2019_00h00 | 7.39E-06 | 1.31E-05 | 1.29E-12 | 5.16E-12 | [5, 6, 7, 8] | Group18 | 4.02 | 8 | [AKT1, CASP3, CDKN1A, CDKN2A, HMGCR, IL1B, MTTP, PTEN] |
| GO:0031072 | heat shock protein binding | GO_MolecularFunction-EBI-UniProt-GOA_27.02.2019_00h00 | 9.86E-06 | 1.69E-05 | 7.49E-07 | 1.36E-06 | [3] | Group15 | 4.73 | 7 | [APOA1, BAX, CDKN1A, HDAC6, HDAC8, HIF1A, KDR] |
| GO:0016538 | cyclin-dependent protein serine/threonine kinase regulator activity | GO_MolecularFunction-EBI-UniProt-GOA_27.02.2019_00h00 | 2.73E-05 | 4.52E-05 | 1.29E-12 | 5.16E-12 | [5, 6, 7, 8, 9] | Group18 | 7.14 | 5 | [CASP3, CCNA2, CDK4, CDKN1A, CDKN2A] |
| GO:0004708 | MAP kinase kinase activity | GO_MolecularFunction-EBI-UniProt-GOA_27.02.2019_00h00 | 3.86E-05 | 6.18E-05 | 1.59E-13 | 7.94E-13 | [5, 7, 8, 9, 10, 11] | Group13 | 4.84 | 6 | [BRAF, EGFR, IGF1R, MAPK14, RAF1, TNF] |
| GO:0042976 | activation of Janus kinase activity | GO_MolecularFunction-EBI-UniProt-GOA_27.02.2019_00h00 | 6.63E-05 | 1.03E-04 | 6.63E-05 | 9.47E-05 | [6, 7, 8, 9] | Group00 | 18.75 | 3 | [CCL5, GH1, IL4] |
| GO:0032413 | negative regulation of ion transmembrane transporter activity | GO_MolecularFunction-EBI-UniProt-GOA_27.02.2019_00h00 | 8.23E-05 | 1.23E-04 | 1.29E-12 | 5.16E-12 | [3, 4, 5] | Group18 | 5.68 | 5 | [CALM1, CALM2, MMP9, PTEN, TIMP1] |
| GO:0045736 | negative regulation of cyclin-dependent protein serine/threonine kinase activity | GO_MolecularFunction-EBI-UniProt-GOA_27.02.2019_00h00 | 1.36E-04 | 1.98E-04 | 1.29E-12 | 5.16E-12 | [4, 5, 6, 7, 8, 9] | Group18 | 7.69 | 4 | [CASP3, CDKN1A, CDKN2A, PTEN] |
| GO:0051879 | Hsp90 protein binding | GO_MolecularFunction-EBI-UniProt-GOA_27.02.2019_00h00 | 1.47E-04 | 2.07E-04 | 7.49E-07 | 1.36E-06 | [4] | Group15 | 7.55 | 4 | [HDAC6, HDAC8, HIF1A, KDR] |
| GO:0016811 | hydrolase activity, acting on carbon-nitrogen (but not peptide) bonds, in linear amides | GO_MolecularFunction-EBI-UniProt-GOA_27.02.2019_00h00 | 2.90E-04 | 3.97E-04 | 7.49E-07 | 1.36E-06 | [4] | Group15 | 4.35 | 5 | [CAT, HDAC6, HDAC8, KLK3, VEGFA] |
| GO:0097199 | cysteine-type endopeptidase activity involved in apoptotic signaling pathway | GO_MolecularFunction-EBI-UniProt-GOA_27.02.2019_00h00 | 3.33E-04 | 4.43E-04 | 3.33E-04 | 4.43E-04 | [8] | Group02 | 11.11 | 3 | [BAX, CASP3, MMP9] |
| GO:0031281 | positive regulation of cyclase activity | GO_MolecularFunction-EBI-UniProt-GOA_27.02.2019_00h00 | 3.71E-04 | 4.81E-04 | 3.71E-04 | 4.64E-04 | [3, 4] | Group06 | 10.71 | 3 | [MAPK14, NOS2, NOS3] |
| GO:0004190 | aspartic-type endopeptidase activity | GO_MolecularFunction-EBI-UniProt-GOA_27.02.2019_00h00 | 5.32E-04 | 6.72E-04 | 5.32E-04 | 6.26E-04 | [6] | Group04 | 5.41 | 4 | [CALM2, CASP3, CXCL8, REN] |
| GO:2000679 | positive regulation of transcription regulatory region DNA binding | GO_MolecularFunction-EBI-UniProt-GOA_27.02.2019_00h00 | 5.54E-04 | 6.81E-04 | 1.29E-12 | 5.16E-12 | [4, 5, 6, 7] | Group18 | 9.38 | 3 | [EP300, IGF1, TGFB1] |
| GO:0005217 | intracellular ligand-gated ion channel activity | GO_MolecularFunction-EBI-UniProt-GOA_27.02.2019_00h00 | 7.84E-04 | 9.41E-04 | 2.51E-22 | 5.02E-21 | [6, 8] | Group19 | 4.88 | 4 | [CALM1, CALM2, CFTR, PRKACA] |
| GO:0043539 | protein serine/threonine kinase activator activity | GO_MolecularFunction-EBI-UniProt-GOA_27.02.2019_00h00 | 9.21E-04 | 1.08E-03 | 1.29E-12 | 5.16E-12 | [5, 6, 7, 8, 9, 10] | Group18 | 7.89 | 3 | [CALM1, CALM2, TGFB1] |
| GO:0042379 | chemokine receptor binding | GO_MolecularFunction-EBI-UniProt-GOA_27.02.2019_00h00 | 1.16E-03 | 1.32E-03 | 1.16E-03 | 1.29E-03 | [5] | Group08 | 4.40 | 4 | [CCL2, CCL5, CXCL8, STAT3] |
| GO:1904031 | positive regulation of cyclin-dependent protein kinase activity | GO_MolecularFunction-EBI-UniProt-GOA_27.02.2019_00h00 | 1.42E-03 | 1.58E-03 | 4.85E-10 | 1.39E-09 | [3, 4, 5, 6, 7, 8] | Group12 | 6.82 | 3 | [AKT1, CDKN1A, EGFR] |
| GO:0060314 | regulation of ryanodine-sensitive calcium-release channel activity | GO_MolecularFunction-EBI-UniProt-GOA_27.02.2019_00h00 | 1.42E-03 | 1.58E-03 | 2.51E-22 | 5.02E-21 | [6, 7, 8, 9, 10, 11, 12] | Group19 | 6.82 | 3 | [CALM1, CALM2, PRKACA] |
| GO:0032148 | activation of protein kinase B activity | GO_MolecularFunction-EBI-UniProt-GOA_27.02.2019_00h00 | 1.51E-03 | 1.65E-03 | 1.51E-03 | 1.59E-03 | [6, 7, 8, 9] | Group07 | 6.67 | 3 | [AKT1, IGF1, INS] |
| GO:0004112 | cyclic-nucleotide phosphodiesterase activity | GO_MolecularFunction-EBI-UniProt-GOA_27.02.2019_00h00 | 1.61E-03 | 1.72E-03 | 6.56E-09 | 1.31E-08 | [6] | Group14 | 6.52 | 3 | [CALM1, CALM2, PDE3A] |
| GO:0090218 | positive regulation of lipid kinase activity | GO_MolecularFunction-EBI-UniProt-GOA_27.02.2019_00h00 | 1.71E-03 | 1.79E-03 | 1.71E-03 | 1.71E-03 | [5, 6, 7] | Group01 | 6.38 | 3 | [F2, KIT, TGFB1] |
| GO:0004407 | histone deacetylase activity | GO_MolecularFunction-EBI-UniProt-GOA_27.02.2019_00h00 | 1.71E-03 | 1.79E-03 | 7.49E-07 | 1.36E-06 | [4, 5] | Group15 | 6.38 | 3 | [HDAC6, HDAC8, VEGFA] |
| GO:0008200 | ion channel inhibitor activity | GO_MolecularFunction-EBI-UniProt-GOA_27.02.2019_00h00 | 2.17E-03 | 2.22E-03 | 2.51E-22 | 5.02E-21 | [4, 5, 6] | Group19 | 5.88 | 3 | [CALM1, CALM2, CFTR] |
| GO:0030544 | Hsp70 protein binding | GO_MolecularFunction-EBI-UniProt-GOA_27.02.2019_00h00 | 2.56E-03 | 2.56E-03 | 7.49E-07 | 1.36E-06 | [4] | Group15 | 5.56 | 3 | [BAX, CDKN1A, HDAC8] |

**Table S6** The data of GO (cell component) enrichment analysis of GATE candidate targets on NS treatment.

| GOID | GOTerm | Ontology Source | Term PValue | Term PValue Corrected with Benjamini-Hochberg | Group PValue | Group PValue Corrected with Benjamini-Hochberg | GOLevels | GOGroups | % Associated Genes | Nr. Genes | Associated Genes Found |
| --- | --- | --- | --- | --- | --- | --- | --- | --- | --- | --- | --- |
| GO:0031093 | platelet alpha granule lumen | GO_CellularComponent-EBI-UniProt-GOA_27.02.2019_00h00 | 9.18E-09 | 4.59E-08 | 9.18E-09 | 2.75E-08 | [5, 6, 7, 8, 9, 10, 11] | Group1 | 9.09 | 8 | [APP, FN1, IGF1, IL6, TGFB1, TGFB2, TIMP1, VEGFA] |
| GO:0030665 | clathrin-coated vesicle membrane | GO_CellularComponent-EBI-UniProt-GOA_27.02.2019_00h00 | 4.95E-05 | 1.24E-04 | 8.17E-06 | 1.23E-05 | [4, 5, 6, 7, 8, 9, 10, 11] | Group2 | 4.38 | 6 | [ADRB2, APOB, CALM2, CFTR, EGFR, LDLR] |
| GO:0034362 | low-density lipoprotein particle | GO_CellularComponent-EBI-UniProt-GOA_27.02.2019_00h00 | 8.12E-05 | 1.35E-04 | 8.17E-06 | 1.23E-05 | [3, 4, 5] | Group2 | 16.67 | 3 | [APOA1, APOB, LDLR] |
| GO:0030669 | clathrin-coated endocytic vesicle membrane | GO_CellularComponent-EBI-UniProt-GOA_27.02.2019_00h00 | 2.30E-03 | 2.87E-03 | 8.17E-06 | 1.23E-05 | [4, 5, 6, 7, 8, 9, 10, 11, 12] | Group2 | 5.45 | 3 | [APOB, CALM2, LDLR] |
| GO:0005778 | peroxisomal membrane | GO_CellularComponent-EBI-UniProt-GOA_27.02.2019_00h00 | 4.93E-03 | 4.93E-03 | 4.93E-03 | 4.93E-03 | [4, 5, 6, 7, 8, 9, 10] | Group0 | 4.17 | 3 | [ALDH3A2, CAT, HMGCR] |

**Table S7** The data of KEGG pathway enrichment analysis of GATE candidate targets on NS treatment.

| ID | Description | pvalue | p.adjust | qvalue | geneID | Count |
| --- | --- | --- | --- | --- | --- | --- |
| hsa04933 | AGE-RAGE signaling pathway in diabetic complications | 5.86E-22 | 1.47E-19 | 4.63E-20 | MAPK14/VEGFA/BAX/TNF/JUN/IL6/CASP3/ICAM1/NOS3/AKT1/STAT3/IL1B/CCL2/SELE/FN1/CXCL8/TGFB1/TGFB2/CDK4/CDC42/HRAS | 21 |
| hsa05205 | Proteoglycans in cancer | 7.99E-19 | 9.99E-17 | 3.15E-17 | EGFR/MAPK14/PRKACA/VEGFA/CDKN1A/TNF/CASP3/TP53/IGF1R/IGF1/AKT1/KDR/FGFR1/STAT3/MMP9/MDM2/RAF1/HIF1A/FN1/TGFB1/TGFB2/BRAF/CDC42/HRAS | 24 |
| hsa05163 | Human cytomegalovirus infection | 8.15E-18 | 5.57E-16 | 1.76E-16 | EGFR/PTGS2/MAPK14/PRKACA/VEGFA/CDKN1A/BAX/TNF/IL6/CASP3/TP53/CALM1/CALM2/AKT1/STAT3/CDKN2A/MDM2/RAF1/IL1B/CCL2/CXCL8/CCL5/CDK4/HRAS | 24 |
| hsa01522 | Endocrine resistance | 8.91E-18 | 5.57E-16 | 1.76E-16 | EGFR/MAPK14/PRKACA/FOS/CDKN1A/BAX/JUN/TP53/IGF1R/IGF1/AKT1/MMP9/CDKN2A/MDM2/RAF1/BRAF/CDK4/HRAS | 18 |
| hsa05214 | Glioma | 5.47E-17 | 2.73E-15 | 8.64E-16 | EGFR/CDKN1A/BAX/TP53/IGF1R/CALM1/CALM2/IGF1/AKT1/CDKN2A/MDM2/RAF1/PTEN/BRAF/CDK4/HRAS | 16 |
| hsa04068 | FoxO signaling pathway | 1.03E-16 | 4.30E-15 | 1.36E-15 | EGFR/MAPK14/CDKN1A/IL6/CAT/IGF1R/SOD2/EP300/IGF1/AKT1/STAT3/MDM2/RAF1/TGFB1/PTEN/TGFB2/INS/BRAF/HRAS | 19 |
| hsa05215 | Prostate cancer | 1.83E-16 | 6.54E-15 | 2.06E-15 | EGFR/HSP90AA1/CDKN1A/TP53/IGF1R/EP300/IGF1/AKT1/FGFR1/MMP9/MDM2/RAF1/PTEN/INS/KLK3/BRAF/HRAS | 17 |
| hsa05161 | Hepatitis B | 3.74E-16 | 1.10E-14 | 3.46E-15 | MAPK14/FOS/CDKN1A/BAX/TNF/JUN/IL6/CASP3/TP53/EP300/AKT1/CCNA2/STAT3/MMP9/RAF1/CXCL8/TGFB1/TGFB2/BRAF/HRAS | 20 |
| hsa05167 | Kaposi sarcoma-associated herpesvirus infection | 3.95E-16 | 1.10E-14 | 3.46E-15 | PTGS2/MAPK14/VEGFA/FOS/CDKN1A/BAX/JUN/IL6/CASP3/TP53/ICAM1/CALM1/CALM2/EP300/AKT1/STAT3/RAF1/HIF1A/CXCL8/CDK4/HRAS | 21 |
| hsa05218 | Melanoma | 8.20E-16 | 2.05E-14 | 6.48E-15 | EGFR/CDKN1A/BAX/TP53/IGF1R/IGF1/AKT1/FGFR1/CDKN2A/MDM2/RAF1/PTEN/BRAF/CDK4/HRAS | 15 |
| hsa05418 | Fluid shear stress and atherosclerosis | 5.69E-15 | 1.29E-13 | 4.09E-14 | MAPK14/HSP90AA1/VEGFA/FOS/TNF/JUN/TP53/ICAM1/NOS3/CALM1/CALM2/AKT1/KDR/MMP9/IL1B/CCL2/SELE/GSTM1 | 18 |
| hsa05219 | Bladder cancer | 8.62E-15 | 1.80E-13 | 5.67E-14 | EGFR/VEGFA/CDKN1A/TP53/MMP9/CDKN2A/MDM2/RAF1/CXCL8/BRAF/CDK4/HRAS | 12 |
| hsa05166 | Human T-cell leukemia virus 1 infection | 1.12E-14 | 2.15E-13 | 6.78E-14 | PRKACA/FOS/CDKN1A/BAX/TNF/JUN/IL6/TP53/ICAM1/EP300/AKT1/IL2/CCNA2/CDKN2A/TGFB1/PTEN/CHEK2/TGFB2/CDK4/XIAP/HRAS | 21 |
| hsa04010 | MAPK signaling pathway | 4.45E-14 | 7.94E-13 | 2.51E-13 | EGFR/MAPK14/PRKACA/VEGFA/FOS/TNF/JUN/CASP3/TP53/IGF1R/IGF1/AKT1/KDR/FGFR1/RAF1/IL1B/TGFB1/TGFB2/INS/KIT/BRAF/CDC42/HRAS | 23 |
| hsa05212 | Pancreatic cancer | 5.03E-14 | 8.38E-13 | 2.65E-13 | EGFR/VEGFA/CDKN1A/BAX/TP53/AKT1/STAT3/CDKN2A/RAF1/TGFB1/TGFB2/BRAF/CDK4/CDC42 | 14 |
| hsa04218 | Cellular senescence | 7.02E-14 | 1.10E-12 | 3.47E-13 | MAPK14/CDKN1A/IL6/TP53/CALM1/CALM2/AKT1/CCNA2/CDKN2A/MDM2/RAF1/CXCL8/TGFB1/PTEN/CHEK2/TGFB2/CDK4/HRAS | 18 |
| hsa01521 | EGFR tyrosine kinase inhibitor resistance | 1.98E-12 | 2.91E-11 | 9.20E-12 | EGFR/VEGFA/BAX/IL6/IGF1R/IGF1/AKT1/KDR/STAT3/RAF1/PTEN/BRAF/HRAS | 13 |
| hsa04151 | PI3K-Akt signaling pathway | 2.12E-12 | 2.95E-11 | 9.32E-12 | EGFR/HSP90AA1/VEGFA/CDKN1A/IL6/TP53/IGF1R/NOS3/IL4/GH1/IGF1/AKT1/IL2/KDR/FGFR1/MDM2/RAF1/FN1/PTEN/INS/KIT/CDK4/HRAS | 23 |
| hsa05224 | Breast cancer | 3.36E-12 | 4.33E-11 | 1.37E-11 | EGFR/FOS/CDKN1A/BAX/JUN/TP53/IGF1R/IGF1/AKT1/FGFR1/RAF1/PTEN/KIT/BRAF/CDK4/HRAS | 16 |
| hsa05142 | Chagas disease (American trypanosomiasis) | 3.47E-12 | 4.33E-11 | 1.37E-11 | MAPK14/FOS/TNF/JUN/IL6/NOS2/AKT1/IL2/IL1B/CCL2/CXCL8/TGFB1/TGFB2/CCL5 | 14 |
| hsa05210 | Colorectal cancer | 6.14E-12 | 7.31E-11 | 2.31E-11 | EGFR/FOS/CDKN1A/BAX/JUN/CASP3/TP53/AKT1/RAF1/TGFB1/TGFB2/BRAF/HRAS | 13 |
| hsa05211 | Renal cell carcinoma | 7.51E-12 | 8.54E-11 | 2.70E-11 | VEGFA/CDKN1A/JUN/EP300/AKT1/RAF1/HIF1A/TGFB1/TGFB2/BRAF/CDC42/HRAS | 12 |
| hsa04066 | HIF-1 signaling pathway | 8.80E-12 | 9.56E-11 | 3.02E-11 | EGFR/VEGFA/CDKN1A/IL6/NOS2/IGF1R/NOS3/EP300/IGF1/AKT1/STAT3/TIMP1/HIF1A/INS | 14 |
| hsa04668 | TNF signaling pathway | 1.28E-11 | 1.34E-10 | 4.22E-11 | PTGS2/MAPK14/FOS/TNF/JUN/IL6/CASP3/ICAM1/AKT1/MMP9/IL1B/CCL2/SELE/CCL5 | 14 |
| hsa04657 | IL-17 signaling pathway | 1.98E-11 | 1.98E-10 | 6.26E-11 | PTGS2/MAPK14/HSP90AA1/FOS/TNF/JUN/IL6/CASP3/IL4/MMP9/IL1B/CCL2/CXCL8 | 13 |
| hsa05220 | Chronic myeloid leukemia | 2.49E-11 | 2.39E-10 | 7.55E-11 | CDKN1A/BAX/TP53/AKT1/CDKN2A/MDM2/RAF1/TGFB1/TGFB2/BRAF/CDK4/HRAS | 12 |
| hsa04625 | C-type lectin receptor signaling pathway | 7.37E-11 | 6.82E-10 | 2.16E-10 | PTGS2/MAPK14/TNF/JUN/IL6/CALM1/CALM2/AKT1/IL2/MDM2/RAF1/IL1B/HRAS | 13 |
| hsa05223 | Non-small cell lung cancer | 9.66E-11 | 8.63E-10 | 2.72E-10 | EGFR/CDKN1A/BAX/TP53/AKT1/STAT3/CDKN2A/RAF1/BRAF/CDK4/HRAS | 11 |
| hsa05323 | Rheumatoid arthritis | 2.87E-10 | 2.48E-09 | 7.82E-10 | VEGFA/FOS/TNF/JUN/IL6/ICAM1/IL1B/CCL2/CXCL8/TGFB1/TGFB2/CCL5 | 12 |
| hsa05225 | Hepatocellular carcinoma | 2.97E-10 | 2.48E-09 | 7.83E-10 | EGFR/CDKN1A/BAX/TP53/IGF1R/AKT1/CDKN2A/RAF1/TGFB1/PTEN/TGFB2/GSTM1/BRAF/CDK4/HRAS | 15 |
| hsa05203 | Viral carcinogenesis | 3.98E-10 | 3.21E-09 | 1.01E-09 | PRKACA/CDKN1A/BAX/JUN/CASP3/TP53/EP300/CCNA2/STAT3/CDKN2A/MDM2/HDAC6/HDAC8/CDK4/CDC42/HRAS | 16 |
| hsa05135 | Yersinia infection | 4.58E-10 | 3.57E-09 | 1.13E-09 | MAPK14/FOS/TNF/JUN/IL6/AKT1/IL2/IL1B/CCL2/FN1/CXCL8/ZAP70/CDC42 | 13 |
| hsa05133 | Pertussis | 4.71E-10 | 3.57E-09 | 1.13E-09 | MAPK14/FOS/TNF/JUN/IL6/NOS2/CASP3/CALM1/CALM2/IL1B/CXCL8 | 11 |
| hsa04015 | Rap1 signaling pathway | 7.62E-10 | 5.60E-09 | 1.77E-09 | EGFR/MAPK14/VEGFA/IGF1R/CALM1/CALM2/IGF1/AKT1/KDR/FGFR1/RAF1/INS/KIT/BRAF/CDC42/HRAS | 16 |
| hsa04660 | T cell receptor signaling pathway | 1.08E-09 | 7.72E-09 | 2.44E-09 | MAPK14/FOS/TNF/JUN/IL4/AKT1/IL2/RAF1/ZAP70/CDK4/CDC42/HRAS | 12 |
| hsa04926 | Relaxin signaling pathway | 1.14E-09 | 7.90E-09 | 2.50E-09 | EGFR/MAPK14/PRKACA/VEGFA/FOS/JUN/NOS2/NOS3/AKT1/MMP9/RAF1/TGFB1/HRAS | 13 |
| hsa04659 | Th17 cell differentiation | 1.51E-09 | 1.02E-08 | 3.22E-09 | MAPK14/HSP90AA1/FOS/JUN/IL6/IL4/IL2/STAT3/HIF1A/IL1B/TGFB1/ZAP70 | 12 |
| hsa05165 | Human papillomavirus infection | 1.90E-09 | 1.25E-08 | 3.94E-09 | EGFR/PTGS2/PRKACA/VEGFA/CDKN1A/BAX/TNF/CASP3/TP53/EP300/AKT1/CCNA2/MDM2/RAF1/FN1/PTEN/CDK4/CDC42/HRAS | 19 |
| hsa05144 | Malaria | 2.65E-09 | 1.67E-08 | 5.28E-09 | TNF/IL6/ICAM1/IL1B/CCL2/SELE/CXCL8/TGFB1/TGFB2 | 9 |
| hsa05235 | PD-L1 expression and PD-1 checkpoint pathway in cancer | 2.68E-09 | 1.67E-08 | 5.28E-09 | EGFR/MAPK14/FOS/JUN/AKT1/STAT3/RAF1/HIF1A/PTEN/ZAP70/HRAS | 11 |
| hsa04014 | Ras signaling pathway | 3.28E-09 | 2.00E-08 | 6.32E-09 | EGFR/PRKACA/VEGFA/IGF1R/CALM1/CALM2/IGF1/AKT1/KDR/FGFR1/RAF1/INS/ZAP70/KIT/CDC42/HRAS | 16 |
| hsa05222 | Small cell lung cancer | 3.84E-09 | 2.28E-08 | 7.21E-09 | PTGS2/CDKN1A/BAX/NOS2/CASP3/TP53/AKT1/FN1/PTEN/CDK4/XIAP | 11 |
| hsa04115 | p53 signaling pathway | 4.64E-09 | 2.70E-08 | 8.52E-09 | CDKN1A/BAX/CASP3/TP53/IGF1/CDKN2A/MDM2/PTEN/CHEK2/CDK4 | 10 |
| hsa05152 | Tuberculosis | 7.54E-09 | 4.29E-08 | 1.35E-08 | MAPK14/BAX/TNF/IL6/NOS2/CASP3/CALM1/CALM2/EP300/AKT1/RAF1/IL1B/TGFB1/TGFB2 | 14 |
| hsa05140 | Leishmaniasis | 9.08E-09 | 5.05E-08 | 1.59E-08 | PTGS2/MAPK14/FOS/TNF/JUN/NOS2/IL4/IL1B/TGFB1/TGFB2 | 10 |
| hsa05213 | Endometrial cancer | 1.05E-08 | 5.70E-08 | 1.80E-08 | EGFR/CDKN1A/BAX/TP53/AKT1/RAF1/PTEN/BRAF/HRAS | 9 |
| hsa05160 | Hepatitis C | 1.10E-08 | 5.83E-08 | 1.84E-08 | EGFR/CDKN1A/BAX/TNF/CASP3/TP53/LDLR/AKT1/STAT3/RAF1/BRAF/CDK4/HRAS | 13 |
| hsa04370 | VEGF signaling pathway | 1.23E-08 | 6.38E-08 | 2.02E-08 | PTGS2/MAPK14/VEGFA/NOS3/AKT1/KDR/RAF1/CDC42/HRAS | 9 |
| hsa04510 | Focal adhesion | 2.74E-08 | 1.40E-07 | 4.41E-08 | EGFR/VEGFA/JUN/IGF1R/IGF1/AKT1/KDR/RAF1/FN1/PTEN/BRAF/CDC42/XIAP/HRAS | 14 |
| hsa04915 | Estrogen signaling pathway | 2.82E-08 | 1.41E-07 | 4.45E-08 | EGFR/HSP90AA1/PRKACA/FOS/JUN/NOS3/CALM1/CALM2/AKT1/MMP9/RAF1/HRAS | 12 |
| hsa05321 | Inflammatory bowel disease (IBD) | 2.96E-08 | 1.45E-07 | 4.58E-08 | TNF/JUN/IL6/IL4/IL2/STAT3/IL1B/TGFB1/TGFB2 | 9 |
| hsa05169 | Epstein-Barr virus infection | 3.11E-08 | 1.49E-07 | 4.72E-08 | MAPK14/CDKN1A/BAX/TNF/JUN/IL6/CASP3/TP53/ICAM1/AKT1/CCNA2/STAT3/MDM2/CDK4 | 14 |
| hsa05164 | Influenza A | 3.35E-08 | 1.58E-07 | 4.98E-08 | BAX/TNF/IL6/CASP3/ICAM1/EP300/AKT1/RAF1/IL1B/CCL2/CXCL8/CCL5/CDK4 | 13 |
| hsa04211 | Longevity regulating pathway | 3.78E-08 | 1.75E-07 | 5.53E-08 | PRKACA/BAX/TP53/CAT/IGF1R/SOD2/IGF1/AKT1/INS/HRAS | 10 |
| hsa05230 | Central carbon metabolism in cancer | 5.06E-08 | 2.30E-07 | 7.26E-08 | EGFR/TP53/AKT1/FGFR1/RAF1/HIF1A/PTEN/KIT/HRAS | 9 |
| hsa04722 | Neurotrophin signaling pathway | 5.92E-08 | 2.64E-07 | 8.34E-08 | MAPK14/BAX/JUN/TP53/CALM1/CALM2/AKT1/RAF1/BRAF/CDC42/HRAS | 11 |
| hsa05132 | Salmonella infection | 6.86E-08 | 3.01E-07 | 9.50E-08 | MAPK14/HSP90AA1/FOS/BAX/TNF/JUN/IL6/CASP3/AKT1/RAF1/IL1B/CXCL8/CDC42/HRAS | 14 |
| hsa01524 | Platinum drug resistance | 8.36E-08 | 3.60E-07 | 1.14E-07 | CDKN1A/BAX/CASP3/TP53/AKT1/CDKN2A/MDM2/GSTM1/XIAP | 9 |
| hsa04914 | Progesterone-mediated oocyte maturation | 1.06E-07 | 4.49E-07 | 1.42E-07 | MAPK14/HSP90AA1/PRKACA/IGF1R/IGF1/AKT1/CCNA2/RAF1/INS/BRAF | 10 |
| hsa05146 | Amoebiasis | 1.41E-07 | 5.88E-07 | 1.86E-07 | PRKACA/TNF/IL6/NOS2/CASP3/IL1B/FN1/CXCL8/TGFB1/TGFB2 | 10 |
| hsa05162 | Measles | 2.73E-07 | 1.12E-06 | 3.53E-07 | FOS/BAX/JUN/IL6/CASP3/TP53/AKT1/IL2/STAT3/IL1B/CDK4 | 11 |
| hsa04213 | Longevity regulating pathway - multiple species | 3.19E-07 | 1.28E-06 | 4.06E-07 | PRKACA/CAT/IGF1R/SOD2/IGF1/AKT1/INS/HRAS | 8 |
| hsa05145 | Toxoplasmosis | 3.43E-07 | 1.36E-06 | 4.30E-07 | MAPK14/TNF/NOS2/CASP3/LDLR/AKT1/STAT3/TGFB1/TGFB2/XIAP | 10 |
| hsa04932 | Non-alcoholic fatty liver disease (NAFLD) | 5.93E-07 | 2.32E-06 | 7.31E-07 | BAX/TNF/JUN/IL6/CASP3/AKT1/IL1B/CXCL8/TGFB1/INS/CDC42 | 11 |
| hsa04935 | Growth hormone synthesis, secretion and action | 6.06E-07 | 2.33E-06 | 7.35E-07 | MAPK14/PRKACA/FOS/EP300/GH1/IGF1/AKT1/STAT3/RAF1/HRAS | 10 |
| hsa04912 | GnRH signaling pathway | 6.93E-07 | 2.62E-06 | 8.28E-07 | EGFR/MAPK14/PRKACA/JUN/CALM1/CALM2/RAF1/CDC42/HRAS | 9 |
| hsa04921 | Oxytocin signaling pathway | 7.73E-07 | 2.89E-06 | 9.11E-07 | EGFR/PTGS2/PRKACA/FOS/CDKN1A/JUN/NOS3/CALM1/CALM2/RAF1/HRAS | 11 |
| hsa04110 | Cell cycle | 8.88E-07 | 3.27E-06 | 1.03E-06 | CDKN1A/TP53/EP300/CCNA2/CDKN2A/MDM2/TGFB1/CHEK2/TGFB2/CDK4 | 10 |
| hsa05206 | MicroRNAs in cancer | 1.14E-06 | 4.11E-06 | 1.30E-06 | EGFR/PTGS2/VEGFA/CDKN1A/CASP3/TP53/EP300/STAT3/MMP9/CDKN2A/MDM2/RAF1/PTEN/TGFB2/HRAS | 15 |
| hsa04913 | Ovarian steroidogenesis | 1.18E-06 | 4.22E-06 | 1.33E-06 | PTGS2/PRKACA/LDLR/IGF1R/IGF1/CYP19A1/INS | 7 |
| hsa04630 | JAK-STAT signaling pathway | 1.37E-06 | 4.81E-06 | 1.52E-06 | EGFR/CDKN1A/IL6/IL4/EP300/GH1/AKT1/IL2/STAT3/RAF1/HRAS | 11 |
| hsa04620 | Toll-like receptor signaling pathway | 1.79E-06 | 6.23E-06 | 1.97E-06 | MAPK14/FOS/TNF/JUN/IL6/AKT1/IL1B/CXCL8/CCL5 | 9 |
| hsa04210 | Apoptosis | 2.08E-06 | 7.12E-06 | 2.25E-06 | FOS/BAX/TNF/JUN/CASP3/TP53/AKT1/RAF1/XIAP/HRAS | 10 |
| hsa05143 | African trypanosomiasis | 2.61E-06 | 8.81E-06 | 2.78E-06 | TNF/IL6/ICAM1/APOA1/IL1B/SELE | 6 |
| hsa04024 | cAMP signaling pathway | 3.65E-06 | 1.22E-05 | 3.84E-06 | ADRB2/PRKACA/PDE3A/FOS/JUN/CALM1/CALM2/EP300/AKT1/RAF1/CFTR/BRAF | 12 |
| hsa05226 | Gastric cancer | 4.75E-06 | 1.56E-05 | 4.94E-06 | EGFR/CDKN1A/BAX/TP53/AKT1/RAF1/TGFB1/TGFB2/BRAF/HRAS | 10 |
| hsa05202 | Transcriptional misregulation in cancer | 5.28E-06 | 1.71E-05 | 5.41E-06 | CDKN1A/BAX/IL6/TP53/IGF1R/IGF1/ELANE/CCNA2/MMP9/MDM2/CXCL8 | 11 |
| hsa04071 | Sphingolipid signaling pathway | 5.52E-06 | 1.77E-05 | 5.59E-06 | MAPK14/BAX/TNF/TP53/NOS3/AKT1/RAF1/PTEN/HRAS | 9 |
| hsa04720 | Long-term potentiation | 7.70E-06 | 2.41E-05 | 7.60E-06 | PRKACA/CALM1/CALM2/EP300/RAF1/BRAF/HRAS | 7 |
| hsa05221 | Acute myeloid leukemia | 7.70E-06 | 2.41E-05 | 7.60E-06 | AKT1/CCNA2/STAT3/RAF1/KIT/BRAF/HRAS | 7 |
| hsa05131 | Shigellosis | 9.08E-06 | 2.80E-05 | 8.85E-06 | EGFR/MAPK14/BAX/TNF/JUN/TP53/AKT1/MDM2/IL1B/CXCL8/CCL5/CDC42 | 12 |
| hsa04917 | Prolactin signaling pathway | 1.03E-05 | 3.11E-05 | 9.83E-06 | MAPK14/FOS/AKT1/STAT3/RAF1/INS/HRAS | 7 |
| hsa05120 | Epithelial cell signaling in Helicobacter pylori infection | 1.03E-05 | 3.11E-05 | 9.83E-06 | EGFR/MAPK14/JUN/CASP3/CXCL8/CCL5/CDC42 | 7 |
| hsa04640 | Hematopoietic cell lineage | 1.16E-05 | 3.42E-05 | 1.08E-05 | TNF/IL6/IL4/GP1BA/IL1B/KIT/DNTT/MME | 8 |
| hsa05130 | Pathogenic Escherichia coli infection | 1.16E-05 | 3.42E-05 | 1.08E-05 | MAPK14/FOS/BAX/TNF/JUN/IL6/CASP3/F2/IL1B/CXCL8/CDC42 | 11 |
| hsa05170 | Human immunodeficiency virus 1 infection | 1.83E-05 | 5.33E-05 | 1.68E-05 | MAPK14/FOS/BAX/TNF/JUN/CASP3/CALM1/CALM2/AKT1/RAF1/HRAS | 11 |
| hsa04621 | NOD-like receptor signaling pathway | 2.64E-05 | 7.59E-05 | 2.40E-05 | MAPK14/HSP90AA1/TNF/JUN/IL6/IL1B/CCL2/CXCL8/CCL5/XIAP | 10 |
| hsa05014 | Amyotrophic lateral sclerosis (ALS) | 3.41E-05 | 9.70E-05 | 3.06E-05 | MAPK14/BAX/TNF/CASP3/TP53/CAT | 6 |
| hsa04726 | Serotonergic synapse | 3.48E-05 | 9.76E-05 | 3.08E-05 | MAOA/PTGS2/PRKACA/CASP3/RAF1/BRAF/APP/HRAS | 8 |
| hsa05020 | Prion diseases | 3.56E-05 | 9.89E-05 | 3.12E-05 | PRKACA/BAX/IL6/IL1B/CCL5 | 5 |
| hsa04012 | ErbB signaling pathway | 3.73E-05 | 1.02E-04 | 3.23E-05 | EGFR/CDKN1A/JUN/AKT1/RAF1/BRAF/HRAS | 7 |
| hsa04062 | Chemokine signaling pathway | 3.83E-05 | 1.04E-04 | 3.29E-05 | PRKACA/AKT1/STAT3/RAF1/CCL2/CXCL8/CCL5/BRAF/CDC42/HRAS | 10 |
| hsa04150 | mTOR signaling pathway | 4.20E-05 | 1.13E-04 | 3.56E-05 | TNF/IGF1R/IGF1/AKT1/RAF1/PTEN/INS/BRAF/HRAS | 9 |
| hsa04919 | Thyroid hormone signaling pathway | 4.45E-05 | 1.18E-04 | 3.73E-05 | PRKACA/TP53/EP300/AKT1/MDM2/RAF1/HIF1A/HRAS | 8 |
| hsa05216 | Thyroid cancer | 4.70E-05 | 1.24E-04 | 3.91E-05 | CDKN1A/BAX/TP53/BRAF/HRAS | 5 |
| hsa04380 | Osteoclast differentiation | 7.48E-05 | 1.95E-04 | 6.15E-05 | MAPK14/FOS/TNF/JUN/AKT1/IL1B/TGFB1/TGFB2 | 8 |
| hsa05231 | Choline metabolism in cancer | 9.31E-05 | 2.39E-04 | 7.53E-05 | EGFR/FOS/JUN/AKT1/RAF1/HIF1A/HRAS | 7 |
| hsa04664 | Fc epsilon RI signaling pathway | 9.35E-05 | 2.39E-04 | 7.53E-05 | MAPK14/TNF/IL4/AKT1/RAF1/HRAS | 6 |
| hsa04924 | Renin secretion | 1.02E-04 | 2.54E-04 | 8.02E-05 | ADRB2/PRKACA/PDE3A/CALM1/CALM2/REN | 6 |
| hsa05031 | Amphetamine addiction | 1.02E-04 | 2.54E-04 | 8.02E-05 | MAOA/PRKACA/FOS/JUN/CALM1/CALM2 | 6 |
| hsa04916 | Melanogenesis | 1.13E-04 | 2.79E-04 | 8.81E-05 | PRKACA/CALM1/CALM2/EP300/RAF1/KIT/HRAS | 7 |
| hsa04064 | NF-kappa B signaling pathway | 1.20E-04 | 2.87E-04 | 9.08E-05 | PTGS2/TNF/ICAM1/IL1B/CXCL8/ZAP70/XIAP | 7 |
| hsa04140 | Autophagy - animal | 1.21E-04 | 2.87E-04 | 9.08E-05 | PRKACA/IGF1R/AKT1/RAF1/HIF1A/PTEN/INS/HRAS | 8 |
| hsa04371 | Apelin signaling pathway | 1.21E-04 | 2.87E-04 | 9.08E-05 | PRKACA/NOS2/NOS3/CALM1/CALM2/AKT1/RAF1/HRAS | 8 |
| hsa04910 | Insulin signaling pathway | 1.21E-04 | 2.87E-04 | 9.08E-05 | PRKACA/CALM1/CALM2/AKT1/RAF1/INS/BRAF/HRAS | 8 |
| hsa04550 | Signaling pathways regulating pluripotency of stem cells | 1.40E-04 | 3.31E-04 | 1.05E-04 | MAPK14/IGF1R/IGF1/AKT1/FGFR1/STAT3/RAF1/HRAS | 8 |
| hsa04928 | Parathyroid hormone synthesis, secretion and action | 1.53E-04 | 3.57E-04 | 1.13E-04 | EGFR/PRKACA/FOS/CDKN1A/FGFR1/RAF1/BRAF | 7 |
| hsa04931 | Insulin resistance | 1.72E-04 | 3.97E-04 | 1.26E-04 | TNF/IL6/NOS3/AKT1/STAT3/PTEN/INS | 7 |
| hsa05034 | Alcoholism | 1.74E-04 | 4.00E-04 | 1.26E-04 | MAOA/PRKACA/CALM1/CALM2/RAF1/HDAC6/HDAC8/BRAF/HRAS | 9 |
| hsa04072 | Phospholipase D signaling pathway | 2.06E-04 | 4.69E-04 | 1.48E-04 | EGFR/AKT1/F2/RAF1/CXCL8/INS/KIT/HRAS | 8 |
| hsa04923 | Regulation of lipolysis in adipocytes | 3.21E-04 | 7.23E-04 | 2.28E-04 | PTGS2/ADRB2/PRKACA/AKT1/INS | 5 |
| hsa04152 | AMPK signaling pathway | 3.29E-04 | 7.35E-04 | 2.32E-04 | IGF1R/IGF1/AKT1/HMGCR/CCNA2/INS/CFTR | 7 |
| hsa01523 | Antifolate resistance | 3.40E-04 | 7.53E-04 | 2.38E-04 | SHMT1/TNF/IL6/IL1B | 4 |
| hsa04060 | Cytokine-cytokine receptor interaction | 3.46E-04 | 7.59E-04 | 2.40E-04 | TNF/IL6/IL4/GH1/IL2/IL1B/CCL2/CXCL8/TGFB1/TGFB2/CCL5 | 11 |
| hsa05134 | Legionellosis | 3.80E-04 | 8.26E-04 | 2.61E-04 | TNF/IL6/CASP3/IL1B/CXCL8 | 5 |
| hsa04611 | Platelet activation | 4.02E-04 | 8.67E-04 | 2.74E-04 | TBXAS1/MAPK14/PRKACA/NOS3/AKT1/GP1BA/F2 | 7 |
| hsa04022 | cGMP-PKG signaling pathway | 4.68E-04 | 1.00E-03 | 3.16E-04 | ADRB2/PDE3A/NOS3/CALM1/CALM2/AKT1/RAF1/INS | 8 |
| hsa04730 | Long-term depression | 4.83E-04 | 1.02E-03 | 3.23E-04 | IGF1R/IGF1/RAF1/BRAF/HRAS | 5 |
| hsa04114 | Oocyte meiosis | 4.88E-04 | 1.02E-03 | 3.24E-04 | MAPK14/PRKACA/IGF1R/CALM1/CALM2/IGF1/INS | 7 |
| hsa04658 | Th1 and Th2 cell differentiation | 4.94E-04 | 1.03E-03 | 3.25E-04 | MAPK14/FOS/JUN/IL4/IL2/ZAP70 | 6 |
| hsa04810 | Regulation of actin cytoskeleton | 5.17E-04 | 1.07E-03 | 3.37E-04 | EGFR/F2/FGFR1/RAF1/FN1/INS/BRAF/CDC42/HRAS | 9 |
| hsa04650 | Natural killer cell mediated cytotoxicity | 5.61E-04 | 1.15E-03 | 3.63E-04 | TNF/CASP3/ICAM1/RAF1/ZAP70/BRAF/HRAS | 7 |
| hsa04728 | Dopaminergic synapse | 5.87E-04 | 1.19E-03 | 3.77E-04 | MAOA/MAPK14/PRKACA/FOS/CALM1/CALM2/AKT1 | 7 |
| hsa04061 | Viral protein interaction with cytokine and cytokine receptor | 7.70E-04 | 1.54E-03 | 4.86E-04 | TNF/IL6/IL2/CCL2/CXCL8/CCL5 | 6 |
| hsa04750 | Inflammatory mediator regulation of TRP channels | 7.70E-04 | 1.54E-03 | 4.86E-04 | MAPK14/PRKACA/CALM1/CALM2/IGF1/IL1B | 6 |
| hsa05332 | Graft-versus-host disease | 1.01E-03 | 2.00E-03 | 6.31E-04 | TNF/IL6/IL2/IL1B | 4 |
| hsa04520 | Adherens junction | 1.05E-03 | 2.06E-03 | 6.51E-04 | EGFR/IGF1R/EP300/FGFR1/CDC42 | 5 |
| hsa04976 | Bile secretion | 1.12E-03 | 2.18E-03 | 6.89E-04 | PRKACA/LDLR/CYP3A4/HMGCR/CFTR | 5 |
| hsa04940 | Type I diabetes mellitus | 1.21E-03 | 2.34E-03 | 7.39E-04 | TNF/IL2/IL1B/INS | 4 |
| hsa04934 | Cushing syndrome | 1.51E-03 | 2.91E-03 | 9.18E-04 | EGFR/PRKACA/CDKN1A/LDLR/CDKN2A/BRAF/CDK4 | 7 |
| hsa04672 | Intestinal immune network for IgA production | 1.97E-03 | 3.76E-03 | 1.19E-03 | IL6/IL4/IL2/TGFB1 | 4 |
| hsa04662 | B cell receptor signaling pathway | 2.00E-03 | 3.76E-03 | 1.19E-03 | FOS/JUN/AKT1/RAF1/HRAS | 5 |
| hsa05204 | Chemical carcinogenesis | 2.00E-03 | 3.76E-03 | 1.19E-03 | PTGS2/CYP3A4/HSD11B1/UGT1A1/GSTM1 | 5 |
| hsa00330 | Arginine and proline metabolism | 2.13E-03 | 3.97E-03 | 1.25E-03 | MAOA/NOS2/NOS3/ALDH3A2 | 4 |
| hsa05010 | Alzheimer disease | 2.64E-03 | 4.90E-03 | 1.55E-03 | TNF/CASP3/CALM1/CALM2/IL1B/MME/APP | 7 |
| hsa05410 | Hypertrophic cardiomyopathy (HCM) | 3.01E-03 | 5.52E-03 | 1.74E-03 | TNF/IL6/IGF1/TGFB1/TGFB2 | 5 |
| hsa05414 | Dilated cardiomyopathy (DCM) | 3.97E-03 | 7.25E-03 | 2.29E-03 | PRKACA/TNF/IGF1/TGFB1/TGFB2 | 5 |
| hsa00140 | Steroid hormone biosynthesis | 4.14E-03 | 7.50E-03 | 2.37E-03 | CYP3A4/HSD11B1/CYP19A1/UGT1A1 | 4 |
| hsa04215 | Apoptosis - multiple species | 5.04E-03 | 9.06E-03 | 2.86E-03 | BAX/CASP3/XIAP | 3 |
| hsa04020 | Calcium signaling pathway | 5.16E-03 | 9.21E-03 | 2.91E-03 | EGFR/ADRB2/PRKACA/NOS2/NOS3/CALM1/CALM2 | 7 |
| hsa04137 | Mitophagy - animal | 5.52E-03 | 9.78E-03 | 3.09E-03 | JUN/TP53/HIF1A/HRAS | 4 |

**Table S8** Detailed information of the interactions among chemical components of GATE, NS, targets and pathways.

| Node1 | Node2 | Net | Node1 | Node2 | Net |
| --- | --- | --- | --- | --- | --- |
| HRAS | RAF1 | ppi | NS | JUN | disease |
| XIAP | CASP3 | ppi | NS | IL6 | disease |
| TP53 | EP300 | ppi | NS | NOS2 | disease |
| JUN | FOS | ppi | NS | CASP3 | disease |
| CDKN1A | TP53 | ppi | NS | TP53 | disease |
| TP53 | MDM2 | ppi | NS | LDLR | disease |
| APOA1 | APOB | ppi | NS | CAT | disease |
| IGF1 | IGF1R | ppi | NS | IGF1R | disease |
| CDKN1A | CCNA2 | ppi | NS | CYP3A4 | disease |
| AKT1 | NOS3 | ppi | NS | ICAM1 | disease |
| CDKN1A | CDK4 | ppi | NS | MTTP | disease |
| CDKN2A | CDK4 | ppi | NS | APOB | disease |
| CCNA2 | CDK4 | ppi | NS | SOD2 | disease |
| VEGFA | KDR | ppi | NS | NOS3 | disease |
| EGFR | STAT3 | ppi | NS | ECE1 | disease |
| INS | IGF1R | ppi | NS | IL4 | disease |
| HIF1A | EP300 | ppi | NS | CALM1 | disease |
| AKT1 | HSP90AA1 | ppi | NS | CALM2 | disease |
| CHEK2 | TP53 | ppi | NS | EP300 | disease |
| HRAS | EGFR | ppi | NS | MT-ND6 | disease |
| SOD2 | CAT | ppi | NS | GH1 | disease |
| AKT1 | INS | ppi | NS | IGF1 | disease |
| MTTP | APOB | ppi | NS | P4HB | disease |
| HRAS | BRAF | ppi | NS | BAP1 | disease |
| MMP9 | TIMP1 | ppi | NS | AKT1 | disease |
| CDKN2A | TP53 | ppi | NS | APOA1 | disease |
| PTEN | TP53 | ppi | NS | IL2 | disease |
| IL6 | STAT3 | ppi | NS | HSD11B1 | disease |
| LDLR | APOB | ppi | NS | HMGCR | disease |
| IL6 | IL4 | ppi | NS | GP1BA | disease |
| VEGFA | HIF1A | ppi | NS | PMS2 | disease |
| JUN | MAPK14 | ppi | NS | F2 | disease |
| AKT1 | CDKN1A | ppi | NS | KDR | disease |
| INS | IGF1 | ppi | NS | ACHE | disease |
| AKT1 | TP53 | ppi | NS | ALDH3A2 | disease |
| AKT1 | STAT3 | ppi | NS | CYP19A1 | disease |
| GP1BA | F2 | ppi | NS | DHODH | disease |
| VEGFA | STAT3 | ppi | NS | ELANE | disease |
| VEGFA | IGF1 | ppi | NS | CCNA2 | disease |
| CCL5 | CXCL8 | ppi | NS | FGFR1 | disease |
| VEGFA | TGFB1 | ppi | NS | STAT3 | disease |
| VEGFA | NOS3 | ppi | NS | MMP9 | disease |
| CDKN2A | MDM2 | ppi | NS | CDKN2A | disease |
| CXCL8 | IL4 | ppi | NS | MDM2 | disease |
| TNF | IL4 | ppi | NS | RAF1 | disease |
| CXCL8 | IL1B | ppi | NS | TIMP1 | disease |
| CALM1 | NOS2 | ppi | NS | HIF1A | disease |
| HSP90AA1 | NOS3 | ppi | NS | IL1B | disease |
| IL1B | IL4 | ppi | NS | CCL2 | disease |
| AKT1 | MDM2 | ppi | NS | SELE | disease |
| IL4 | CCL2 | ppi | NS | FN1 | disease |
| JUN | TP53 | ppi | NS | CXCL8 | disease |
| HSP90AA1 | TP53 | ppi | NS | TGFB1 | disease |
| TP53 | MAPK14 | ppi | NS | PTEN | disease |
| CCNA2 | TP53 | ppi | NS | UGT1A1 | disease |
| AKT1 | PTEN | ppi | NS | CHEK2 | disease |
| VEGFA | MMP9 | ppi | NS | TGFB2 | disease |
| CASP3 | MAPK14 | ppi | NS | INS | disease |
| HSP90AA1 | EGFR | ppi | NS | KLK3 | disease |
| TNF | IL1B | ppi | NS | HDAC6 | disease |
| FOS | STAT3 | ppi | NS | CFTR | disease |
| VEGFA | FN1 | ppi | NS | ZAP70 | disease |
| AKT1 | HIF1A | ppi | NS | GSTM1 | disease |
| MTTP | P4HB | ppi | NS | TGM3 | disease |
| TNF | JUN | ppi | NS | KIT | disease |
| TP53 | STAT3 | ppi | NS | DNTT | disease |
| HIF1A | STAT3 | ppi | NS | MME | disease |
| CALM1 | NOS3 | ppi | NS | CCL5 | disease |
| IL6 | CXCL8 | ppi | NS | HDAC8 | disease |
| FN1 | TGFB1 | ppi | NS | BRAF | disease |
| HSP90AA1 | HDAC6 | ppi | NS | REN | disease |
| CDKN2A | HRAS | ppi | NS | APP | disease |
| TNF | MAPK14 | ppi | NS | CDK4 | disease |
| BAX | TP53 | ppi | NS | CDC42 | disease |
| IL6 | JUN | ppi | NS | XIAP | disease |
| TNF | IL6 | ppi | NS | HRAS | disease |
| TNF | NOS2 | ppi | NS | HPRT1 | disease |
| IL6 | IL1B | ppi | Daidzein | GATE | comp |
| CXCL8 | STAT3 | ppi | TBXAS1 | Daidzein | target |
| VEGFA | JUN | ppi | MAOA | Daidzein | target |
| NOS3 | KDR | ppi | EGFR | Daidzein | target |
| IL6 | TIMP1 | ppi | SHMT1 | Daidzein | target |
| IL6 | CCL2 | ppi | PTGS2 | Daidzein | target |
| HSP90AA1 | STAT3 | ppi | ADRB2 | Daidzein | target |
| IL6 | FOS | ppi | MAPK14 | Daidzein | target |
| MMP9 | STAT3 | ppi | HSP90AA1 | Daidzein | target |
| CDKN1A | JUN | ppi | PRKACA | Daidzein | target |
| CDKN2A | CDKN1A | ppi | PDE3A | Daidzein | target |
| STAT3 | CCL2 | ppi | VEGFA | Daidzein | target |
| CHEK2 | MDM2 | ppi | FOS | Daidzein | target |
| TNF | FOS | ppi | CDKN1A | Daidzein | target |
| PTGS2 | STAT3 | ppi | BAX | Daidzein | target |
| STAT3 | IL4 | ppi | TNF | Daidzein | target |
| MTTP | APOA1 | ppi | JUN | Daidzein | target |
| CDKN1A | CASP3 | ppi | IL6 | Daidzein | target |
| TGFB1 | TIMP1 | ppi | NOS2 | Daidzein | target |
| HSP90AA1 | KDR | ppi | CASP3 | Daidzein | target |
| CXCL8 | CCL2 | ppi | TP53 | Daidzein | target |
| JUN | EP300 | ppi | LDLR | Daidzein | target |
| VEGFA | EGFR | ppi | CAT | Daidzein | target |
| HDAC6 | EP300 | ppi | IGF1R | Daidzein | target |
| AKT1 | RAF1 | ppi | CYP3A4 | Daidzein | target |
| IL1B | MAPK14 | ppi | ICAM1 | Daidzein | target |
| JUN | CCL2 | ppi | MTTP | Daidzein | target |
| AKT1 | IL2 | ppi | APOB | Daidzein | target |
| FOS | MAPK14 | ppi | SOD2 | Daidzein | target |
| HIF1A | TP53 | ppi | NOS3 | Daidzein | target |
| TGFB2 | FN1 | ppi | ECE1 | Daidzein | target |
| TNF | STAT3 | ppi | IL4 | Daidzein | target |
| HIF1A | HSP90AA1 | ppi | CALM1 | Daidzein | target |
| AKT1 | EP300 | ppi | CALM2 | Daidzein | target |
| HRAS | KIT | ppi | EP300 | Daidzein | target |
| PRKACA | BRAF | ppi | MT-ND6 | Daidzein | target |
| CDKN1A | STAT3 | ppi | GH1 | Daidzein | target |
| STAT3 | EP300 | ppi | IGF1 | Daidzein | target |
| UGT1A1 | CYP3A4 | ppi | P4HB | Daidzein | target |
| HRAS | FGFR1 | ppi | BAP1 | Daidzein | target |
| EP300 | MDM2 | ppi | NOS3 | Daidzein | target |
| ICAM1 | STAT3 | ppi | AKT1 | Daidzein | target |
| PTEN | XIAP | ppi | APOA1 | Daidzein | target |
| PTGS2 | IL1B | ppi | APOB | Daidzein | target |
| VEGFA | HSP90AA1 | ppi | Daidzin | GATE | comp |
| CXCL8 | MAPK14 | ppi | IL2 | Daidzin | target |
| TNF | CXCL8 | ppi | HSD11B1 | Daidzin | target |
| HIF1A | EGFR | ppi | SOD2 | Daidzin | target |
| AKT1 | SOD2 | ppi | HMGCR | Daidzin | target |
| VEGFA | EP300 | ppi | GP1BA | Daidzin | target |
| VEGFA | TIMP1 | ppi | PMS2 | Daidzin | target |
| CCL5 | STAT3 | ppi | NOS2 | Daidzin | target |
| FN1 | TIMP1 | ppi | F2 | Daidzin | target |
| HRAS | IGF1R | ppi | PTGS2 | Daidzin | target |
| CDC42 | EGFR | ppi | CALM2 | Daidzin | target |
| STAT3 | MAPK14 | ppi | KDR | Daidzin | target |
| VEGFA | CDC42 | ppi | ACHE | Daidzin | target |
| AKT1 | CAT | ppi | MAOA | Daidzin | target |
| JUN | CXCL8 | ppi | ALDH3A2 | Daidzin | target |
| IL6 | FN1 | ppi | Genistein | GATE | comp |
| IL6 | APOA1 | ppi | TBXAS1 | Genistein | target |
| TNF | CCL2 | ppi | MAOA | Genistein | target |
| PTEN | MDM2 | ppi | EGFR | Genistein | target |
| JUN | IL2 | ppi | CYP19A1 | Genistein | target |
| STAT3 | TIMP1 | ppi | DHODH | Genistein | target |
| HIF1A | JUN | ppi | ELANE | Genistein | target |
| FOS | EP300 | ppi | HSP90AA1 | Genistein | target |
| LDLR | APOA1 | ppi | SHMT1 | Genistein | target |
| FOS | CCL2 | ppi | CCNA2 | Genistein | target |
| CDKN1A | EP300 | ppi | FGFR1 | Genistein | target |
| FOS | TP53 | ppi | PTGS2 | Genistein | target |
| HIF1A | NOS3 | ppi | MAPK14 | Genistein | target |
| IGF1 | TGFB1 | ppi | HSP90AA1 | Genistein | target |
| STAT3 | IL2 | ppi | PRKACA | Genistein | target |
| AKT1 | CASP3 | ppi | EGFR | Genistein | target |
| CDC42 | FN1 | ppi | STAT3 | Genistein | target |
| FN1 | IGF1 | ppi | AKT1 | Genistein | target |
| HDAC8 | EP300 | ppi | VEGFA | Genistein | target |
| INS | EGFR | ppi | FOS | Genistein | target |
| PTEN | EGFR | ppi | CDKN1A | Genistein | target |
| HRAS | TP53 | ppi | BAX | Genistein | target |
| HRAS | INS | ppi | MMP9 | Genistein | target |
| VEGFA | AKT1 | ppi | TNF | Genistein | target |
| HIF1A | MDM2 | ppi | JUN | Genistein | target |
| HRAS | IL2 | ppi | NOS2 | Genistein | target |
| INS | APP | ppi | CDKN2A | Genistein | target |
| STAT3 | IL1B | ppi | CASP3 | Genistein | target |
| ADRB2 | CFTR | ppi | TP53 | Genistein | target |
| NOS2 | CALM2 | ppi | LDLR | Genistein | target |
| IL4 | IL2 | ppi | MDM2 | Genistein | target |
| HSP90AA1 | EP300 | ppi | RAF1 | Genistein | target |
| STAT3 | TGFB1 | ppi | TIMP1 | Genistein | target |
| P4HB | APOB | ppi | HIF1A | Genistein | target |
| EGFR | TP53 | ppi | IGF1R | Genistein | target |
| TGFB2 | APP | ppi | ICAM1 | Genistein | target |
| JUN | IL1B | ppi | IL1B | Genistein | target |
| HRAS | HSP90AA1 | ppi | CCL2 | Genistein | target |
| TP53 | PMS2 | ppi | SELE | Genistein | target |
| IL4 | MAPK14 | ppi | FN1 | Genistein | target |
| APP | TGFB1 | ppi | CXCL8 | Genistein | target |
| CDKN1A | HSP90AA1 | ppi | SOD2 | Genistein | target |
| TNF | EP300 | ppi | NOS3 | Genistein | target |
| TNF | ICAM1 | ppi | TGFB1 | Genistein | target |
| CCNA2 | MDM2 | ppi | PTEN | Genistein | target |
| FN1 | IGF1R | ppi | HMGCR | Genistein | target |
| TNF | XIAP | ppi | UGT1A1 | Genistein | target |
| AKT1 | IGF1 | ppi | CHEK2 | Genistein | target |
| HIF1A | CDKN2A | ppi | TGFB2 | Genistein | target |
| CXCL8 | EP300 | ppi | INS | Genistein | target |
| KIT | STAT3 | ppi | KLK3 | Genistein | target |
| CDC42 | MAPK14 | ppi | HDAC6 | Genistein | target |
| VEGFA | IL6 | ppi | APOA1 | Genistein | target |
| IL6 | IL2 | ppi | CFTR | Genistein | target |
| NOS2 | CAT | ppi | NOS3 | Genistein | target |
| HRAS | FN1 | ppi | CYP19A1 | Genistein | target |
| HRAS | IGF1 | ppi | CFTR | Genistein | target |
| PTGS2 | CXCL8 | ppi | AKT1 | Genistein | target |
| TNF | IL2 | ppi | Genistin | GATE | comp |
| IL6 | APOB | ppi | IL2 | Genistin | target |
| IL6 | APP | ppi | SOD2 | Genistin | target |
| CALM1 | CALM2 | ppi | ZAP70 | Genistin | target |
| APP | APOA1 | ppi | HSD11B1 | Genistin | target |
| JUN | NOS2 | ppi | SELE | Genistin | target |
| KLK3 | IGF1 | ppi | GSTM1 | Genistin | target |
| CDKN1A | HDAC6 | ppi | TGM3 | Genistin | target |
| CDC42 | KDR | ppi | PMS2 | Genistin | target |
| NOS2 | STAT3 | ppi | KIT | Genistin | target |
| JUN | IL4 | ppi | ELANE | Genistin | target |
| VEGFA | CXCL8 | ppi | NOS2 | Genistin | target |
| SELE | ICAM1 | ppi | F2 | Genistin | target |
| FN1 | IL4 | ppi | PTGS2 | Genistin | target |
| IL6 | ICAM1 | ppi | CALM2 | Genistin | target |
| VEGFA | KIT | ppi | CALM1 | Genistin | target |
| IGF1 | TIMP1 | ppi | DNTT | Genistin | target |
| VEGFA | TGFB2 | ppi | Glycitein | GATE | comp |
| IGF1 | RAF1 | ppi | EGFR | Glycitein | target |
| TBXAS1 | PTGS2 | ppi | MME | Glycitein | target |
| JUN | NOS3 | ppi | CCL5 | Glycitein | target |
| BRAF | RAF1 | ppi | SHMT1 | Glycitein | target |
| TGFB2 | IGF1 | ppi | MAPK14 | Glycitein | target |
| HDAC8 | HDAC6 | ppi | HDAC8 | Glycitein | target |
| IGF1 | APP | ppi | BRAF | Glycitein | target |
| EP300 | MAPK14 | ppi | REN | Glycitein | target |
| IL1B | CCL2 | ppi | PTGS2 | Glycitein | target |
| JUN | TGFB1 | ppi | PDE3A | Glycitein | target |
| FN1 | EGFR | ppi | MAPK14 | Glycitein | target |
| IL6 | INS | ppi | HSP90AA1 | Glycitein | target |
| FN1 | RAF1 | ppi | CCNA2 | Glycitein | target |
| FN1 | APP | ppi | CALM1 | Glycitein | target |
| ALDH3A2 | CAT | ppi | PRKACA | Glycitein | target |
| IL6 | PTGS2 | ppi | NOS2 | Glycitein | target |
| CASP3 | TP53 | ppi | APP | Glycitein | target |
| F2 | APP | ppi | JUN | Glycitein | target |
| TNF | MMP9 | ppi | CDK4 | Glycitein | target |
| VEGFA | APP | ppi | Isoschaftoside | GATE | comp |
| FN1 | APOA1 | ppi | CDC42 | Isoschaftoside | target |
| ADRB2 | APOB | ppi | XIAP | Isoschaftoside | target |
| FOS | TGFB1 | ppi | PMS2 | Isoschaftoside | target |
| LDLR | EGFR | ppi | HRAS | Isoschaftoside | target |
| TGFB2 | TIMP1 | ppi | HPRT1 | Isoschaftoside | target |
| MAPK14 | IL2 | ppi | EGFR | PI3K-Akt signaling pathway | pathway |
| CCL5 | TNF | ppi | VEGFA | PI3K-Akt signaling pathway | pathway |
| HSP90AA1 | IL2 | ppi | CDKN1A | PI3K-Akt signaling pathway | pathway |
| CDKN1A | MDM2 | ppi | HSP90AA1 | PI3K-Akt signaling pathway | pathway |
| FN1 | P4HB | ppi | KIT | PI3K-Akt signaling pathway | pathway |
| AKT1 | CDC42 | ppi | IL6 | PI3K-Akt signaling pathway | pathway |
| FN1 | APOB | ppi | TP53 | PI3K-Akt signaling pathway | pathway |
| F2 | IGF1 | ppi | IGF1R | PI3K-Akt signaling pathway | pathway |
| CALM1 | HSP90AA1 | ppi | IGF1 | PI3K-Akt signaling pathway | pathway |
| HIF1A | CDKN1A | ppi | AKT1 | PI3K-Akt signaling pathway | pathway |
| ADRB2 | EGFR | ppi | HRAS | PI3K-Akt signaling pathway | pathway |
| CYP19A1 | CYP3A4 | ppi | KDR | PI3K-Akt signaling pathway | pathway |
| EGFR | CFTR | ppi | FGFR1 | PI3K-Akt signaling pathway | pathway |
| HDAC8 | PRKACA | ppi | MDM2 | PI3K-Akt signaling pathway | pathway |
| TGFB2 | TGFB1 | ppi | RAF1 | PI3K-Akt signaling pathway | pathway |
| CXCL8 | APP | ppi | FN1 | PI3K-Akt signaling pathway | pathway |
| CCL5 | IL6 | ppi | NOS3 | PI3K-Akt signaling pathway | pathway |
| MMP9 | CXCL8 | ppi | IL4 | PI3K-Akt signaling pathway | pathway |
| HRAS | F2 | ppi | GH1 | PI3K-Akt signaling pathway | pathway |
| TNF | SELE | ppi | IL2 | PI3K-Akt signaling pathway | pathway |
| APOA1 | TIMP1 | ppi | PTEN | PI3K-Akt signaling pathway | pathway |
| P4HB | APP | ppi | INS | PI3K-Akt signaling pathway | pathway |
| MAOA | CYP3A4 | ppi | CDK4 | PI3K-Akt signaling pathway | pathway |
| FN1 | BRAF | ppi | EGFR | FoxO signaling pathway | pathway |
| EP300 | TGFB1 | ppi | MAPK14 | FoxO signaling pathway | pathway |
| AKT1 | MAPK14 | ppi | CDKN1A | FoxO signaling pathway | pathway |
| HSD11B1 | CYP3A4 | ppi | IL6 | FoxO signaling pathway | pathway |
| ICAM1 | CCL2 | ppi | IGF1R | FoxO signaling pathway | pathway |
| CXCL8 | ICAM1 | ppi | IGF1 | FoxO signaling pathway | pathway |
| ALDH3A2 | MAOA | ppi | AKT1 | FoxO signaling pathway | pathway |
| APP | APOB | ppi | HRAS | FoxO signaling pathway | pathway |
| NOS3 | EP300 | ppi | STAT3 | FoxO signaling pathway | pathway |
| JUN | TGFB2 | ppi | MDM2 | FoxO signaling pathway | pathway |
| ICAM1 | IL1B | ppi | RAF1 | FoxO signaling pathway | pathway |
| APP | TIMP1 | ppi | TGFB1 | FoxO signaling pathway | pathway |
| TNF | PTGS2 | ppi | TGFB2 | FoxO signaling pathway | pathway |
| IL6 | P4HB | ppi | BRAF | FoxO signaling pathway | pathway |
| INS | KIT | ppi | PTEN | FoxO signaling pathway | pathway |
| LDLR | CFTR | ppi | INS | FoxO signaling pathway | pathway |
| HSP90AA1 | PRKACA | ppi | CAT | FoxO signaling pathway | pathway |
| APOB | CFTR | ppi | SOD2 | FoxO signaling pathway | pathway |
| EGFR | APOB | ppi | EP300 | FoxO signaling pathway | pathway |
| IL1B | EP300 | ppi | CDKN1A | Hepatitis B | pathway |
| TGFB2 | FOS | ppi | IL6 | Hepatitis B | pathway |
| IL6 | MMP9 | ppi | TP53 | Hepatitis B | pathway |
| IGF1R | RAF1 | ppi | CASP3 | Hepatitis B | pathway |
| F2 | CFTR | ppi | MAPK14 | Hepatitis B | pathway |
| CCL5 | APP | ppi | AKT1 | Hepatitis B | pathway |
| JUN | STAT3 | ppi | HRAS | Hepatitis B | pathway |
| IGF1R | MDM2 | ppi | STAT3 | Hepatitis B | pathway |
| KDR | MAPK14 | ppi | MMP9 | Hepatitis B | pathway |
| IL6 | EGFR | ppi | RAF1 | Hepatitis B | pathway |
| VEGFA | INS | ppi | TGFB1 | Hepatitis B | pathway |
| LDLR | ADRB2 | ppi | TGFB2 | Hepatitis B | pathway |
| VEGFA | TNF | ppi | BRAF | Hepatitis B | pathway |
| APOB | TIMP1 | ppi | FOS | Hepatitis B | pathway |
| P4HB | APOA1 | ppi | BAX | Hepatitis B | pathway |
| HIF1A | P4HB | ppi | TNF | Hepatitis B | pathway |
| VEGFA | PTGS2 | ppi | JUN | Hepatitis B | pathway |
| VEGFA | TP53 | ppi | EP300 | Hepatitis B | pathway |
| TNF | INS | ppi | CCNA2 | Hepatitis B | pathway |
| HRAS | PTEN | ppi | CXCL8 | Hepatitis B | pathway |
| GH1 | STAT3 | ppi | VEGFA | AGE-RAGE signaling pathway in diabetic complications | pathway |
| P4HB | TIMP1 | ppi | IL6 | AGE-RAGE signaling pathway in diabetic complications | pathway |
| VEGFA | IGF1R | ppi | MAPK14 | AGE-RAGE signaling pathway in diabetic complications | pathway |
| EP300 | APOA1 | ppi | Bax | AGE-RAGE signaling pathway in diabetic complications | pathway |
| XIAP | TGFB1 | ppi | TNF | AGE-RAGE signaling pathway in diabetic complications | pathway |
| PTGS2 | TP53 | ppi | CASP3 | AGE-RAGE signaling pathway in diabetic complications | pathway |
| INS | NOS3 | ppi | ICAM1 | AGE-RAGE signaling pathway in diabetic complications | pathway |
| VEGFA | CCL2 | ppi | NOS3 | AGE-RAGE signaling pathway in diabetic complications | pathway |
| AKT1 | XIAP | ppi | AKT1 | AGE-RAGE signaling pathway in diabetic complications | pathway |
| MMP9 | IL1B | ppi | STAT3 | AGE-RAGE signaling pathway in diabetic complications | pathway |
| AKT1 | JUN | ppi | IL1B | AGE-RAGE signaling pathway in diabetic complications | pathway |
| HSP90AA1 | RAF1 | ppi | CCL2 | AGE-RAGE signaling pathway in diabetic complications | pathway |
| JUN | APOA1 | ppi | SELE | AGE-RAGE signaling pathway in diabetic complications | pathway |
| APOB | MAPK14 | ppi | FN1 | AGE-RAGE signaling pathway in diabetic complications | pathway |
| PRKACA | IGF1R | ppi | CXCL8 | AGE-RAGE signaling pathway in diabetic complications | pathway |
| BAX | EP300 | ppi | TGFB1 | AGE-RAGE signaling pathway in diabetic complications | pathway |
| F2 | ADRB2 | ppi | TGFB2 | AGE-RAGE signaling pathway in diabetic complications | pathway |
| AKT1 | NOS2 | ppi | CDK4 | AGE-RAGE signaling pathway in diabetic complications | pathway |
| F2 | RAF1 | ppi | CDC42 | AGE-RAGE signaling pathway in diabetic complications | pathway |
| IGF1 | KIT | ppi | HRAS | AGE-RAGE signaling pathway in diabetic complications | pathway |
| GP1BA | RAF1 | ppi | JUN | AGE-RAGE signaling pathway in diabetic complications | pathway |
| HSP90AA1 | CDK4 | ppi | EGFR | Endocrine resistance | pathway |
| MAOA | IL4 | ppi | MAPK14 | Endocrine resistance | pathway |
| GP1BA | EP300 | ppi | PRKACA | Endocrine resistance | pathway |
| CDKN2A | PTEN | ppi | FOS | Endocrine resistance | pathway |
| PTGS2 | NOS2 | ppi | CDKN1A | Endocrine resistance | pathway |
| TP53 | IGF1R | ppi | BAX | Endocrine resistance | pathway |
| MMP9 | TGFB1 | ppi | JUN | Endocrine resistance | pathway |
| INS | REN | ppi | TP53 | Endocrine resistance | pathway |
| DNTT | CASP3 | ppi | IGF1R | Endocrine resistance | pathway |
| CASP3 | MDM2 | ppi | IGF1 | Endocrine resistance | pathway |
| VEGFA | PRKACA | ppi | AKT1 | Endocrine resistance | pathway |
| AKT1 | HRAS | ppi | MMP9 | Endocrine resistance | pathway |
| PRKACA | KDR | ppi | CDKN2A | Endocrine resistance | pathway |
| FN1 | PRKACA | ppi | MDM2 | Endocrine resistance | pathway |
| IL1B | IL2 | ppi | RAF1 | Endocrine resistance | pathway |
| PRKACA | NOS3 | ppi | BRAF | Endocrine resistance | pathway |
| FOS | EGFR | ppi | CDK4 | Endocrine resistance | pathway |
| INS | APOB | ppi | HRAS | Endocrine resistance | pathway |
| CDKN2A | MAPK14 | ppi | MAPK14 | Fluid shear stress and atherosclerosis | pathway |
| CCL5 | JUN | ppi | HSP90AA1 | Fluid shear stress and atherosclerosis | pathway |
| MMP9 | JUN | ppi | VEGFA | Fluid shear stress and atherosclerosis | pathway |
| F2 | APOA1 | ppi | FOS | Fluid shear stress and atherosclerosis | pathway |
| PRKACA | FOS | ppi | TNF | Fluid shear stress and atherosclerosis | pathway |
| CASP3 | APP | ppi | JUN | Fluid shear stress and atherosclerosis | pathway |
| VEGFA | ICAM1 | ppi | TP53 | Fluid shear stress and atherosclerosis | pathway |
| AKT1 | FN1 | ppi | ICAM1 | Fluid shear stress and atherosclerosis | pathway |
| AKT1 | IL6 | ppi | CALM2 | Fluid shear stress and atherosclerosis | pathway |
| AKT1 | MMP9 | ppi | AKT1 | Fluid shear stress and atherosclerosis | pathway |
| IGF1R | STAT3 | ppi | KDR | Fluid shear stress and atherosclerosis | pathway |
| GH1 | IGF1 | ppi | MMP9 | Fluid shear stress and atherosclerosis | pathway |
| INS | CCL2 | ppi | IL1B | Fluid shear stress and atherosclerosis | pathway |
| PRKACA | RAF1 | ppi | CCL2 | Fluid shear stress and atherosclerosis | pathway |
| INS | PTEN | ppi | SELE | Fluid shear stress and atherosclerosis | pathway |
| VEGFA | FGFR1 | ppi | GSTM1 | Fluid shear stress and atherosclerosis | pathway |
| IGF1 | EGFR | ppi | NOS3 | Fluid shear stress and atherosclerosis | pathway |
| CCL5 | IL1B | ppi | CALM1 | Fluid shear stress and atherosclerosis | pathway |
| CDC42 | PTEN | ppi | PRKACA | Human T-cell leukemia virus 1 infection | pathway |
| JUN | EGFR | ppi | FOS | Human T-cell leukemia virus 1 infection | pathway |
| VEGFA | IL1B | ppi | CDKN1A | Human T-cell leukemia virus 1 infection | pathway |
| AKT1 | TNF | ppi | BAX | Human T-cell leukemia virus 1 infection | pathway |
| JUN | CASP3 | ppi | TNF | Human T-cell leukemia virus 1 infection | pathway |
| CASP3 | BAX | ppi | JUN | Human T-cell leukemia virus 1 infection | pathway |
| TP53 | CDK4 | ppi | IL6 | Human T-cell leukemia virus 1 infection | pathway |
| SOD2 | TP53 | ppi | TP53 | Human T-cell leukemia virus 1 infection | pathway |
| TNF | CASP3 | ppi | ICAM1 | Human T-cell leukemia virus 1 infection | pathway |
| LDLR | HMGCR | ppi | EP300 | Human T-cell leukemia virus 1 infection | pathway |
| PTGS2 | EGFR | ppi | AKT1 | Human T-cell leukemia virus 1 infection | pathway |
| PRKACA | CFTR | ppi | IL2 | Human T-cell leukemia virus 1 infection | pathway |
| IL6 | TP53 | ppi | CCNA2 | Human T-cell leukemia virus 1 infection | pathway |
| KIT | TP53 | ppi | CDKN2A | Human T-cell leukemia virus 1 infection | pathway |
| VEGFA | CCL5 | ppi | TGFB1 | Human T-cell leukemia virus 1 infection | pathway |
| CXCL8 | IL2 | ppi | PTEN | Human T-cell leukemia virus 1 infection | pathway |
| ICAM1 | IL4 | ppi | CHEK2 | Human T-cell leukemia virus 1 infection | pathway |
| IL6 | IGF1 | ppi | TGFB2 | Human T-cell leukemia virus 1 infection | pathway |
| EGFR | MDM2 | ppi | CDK4 | Human T-cell leukemia virus 1 infection | pathway |
| PTGS2 | CCL2 | ppi | XIAP | Human T-cell leukemia virus 1 infection | pathway |
| ZAP70 | MAPK14 | ppi | HRAS | Human T-cell leukemia virus 1 infection | pathway |
| CASP3 | STAT3 | ppi | EGFR | MAPK signaling pathway | pathway |
| AKT1 | PTGS2 | ppi | MAPK14 | MAPK signaling pathway | pathway |
| TP53 | KDR | ppi | PRKACA | MAPK signaling pathway | pathway |
| PTEN | IGF1R | ppi | VEGFA | MAPK signaling pathway | pathway |
| NOS2 | IL1B | ppi | FOS | MAPK signaling pathway | pathway |
| INS | KDR | ppi | TNF | MAPK signaling pathway | pathway |
| MMP9 | CCL2 | ppi | JUN | MAPK signaling pathway | pathway |
| CCL5 | ICAM1 | ppi | CASP3 | MAPK signaling pathway | pathway |
| IGF1 | KDR | ppi | TP53 | MAPK signaling pathway | pathway |
| NOS3 | CALM2 | ppi | IGF1R | MAPK signaling pathway | pathway |
| CYP3A4 | GSTM1 | ppi | IGF1 | MAPK signaling pathway | pathway |
| ELANE | CXCL8 | ppi | AKT1 | MAPK signaling pathway | pathway |
| NOS3 | REN | ppi | KDR | MAPK signaling pathway | pathway |
| INS | FN1 | ppi | FGFR1 | MAPK signaling pathway | pathway |
| IL6 | TGFB1 | ppi | RAF1 | MAPK signaling pathway | pathway |
| VEGFA | PTEN | ppi | IL1B | MAPK signaling pathway | pathway |
| AKT1 | KIT | ppi | TGFB1 | MAPK signaling pathway | pathway |
| INS | STAT3 | ppi | TGFB2 | MAPK signaling pathway | pathway |
| JUN | CDK4 | ppi | INS | MAPK signaling pathway | pathway |
| IL6 | MAPK14 | ppi | KIT | MAPK signaling pathway | pathway |
| PTGS2 | NOS3 | ppi | BRAF | MAPK signaling pathway | pathway |
| ACHE | APP | ppi | CDC42 | MAPK signaling pathway | pathway |
| ICAM1 | IL2 | ppi | HRAS | MAPK signaling pathway | pathway |
| INS | JUN | ppi | MAPK14 | Cellular senescence | pathway |
| TNF | NOS3 | ppi | CDKN1A | Cellular senescence | pathway |
| CASP3 | IL1B | ppi | IL6 | Cellular senescence | pathway |
| VEGFA | HRAS | ppi | TP53 | Cellular senescence | pathway |
| MMP9 | EGFR | ppi | CALM1 | Cellular senescence | pathway |
| PTEN | STAT3 | ppi | CALM2 | Cellular senescence | pathway |
| HRAS | FOS | ppi | AKT1 | Cellular senescence | pathway |
| F2 | APOB | ppi | CCNA2 | Cellular senescence | pathway |
| TNF | TP53 | ppi | CDKN2A | Cellular senescence | pathway |
| JUN | PRKACA | ppi | MDM2 | Cellular senescence | pathway |
| TNF | FN1 | ppi | RAF1 | Cellular senescence | pathway |
| INS | GH1 | ppi | CXCL8 | Cellular senescence | pathway |
| FOS | IL2 | ppi | TGFB1 | Cellular senescence | pathway |
| PTEN | JUN | ppi | PTEN | Cellular senescence | pathway |
| VEGFA | SELE | ppi | CHEK2 | Cellular senescence | pathway |
| IL1B | TIMP1 | ppi | TGFB2 | Cellular senescence | pathway |
| FN1 | ICAM1 | ppi | CDK4 | Cellular senescence | pathway |
| AKT1 | ICAM1 | ppi | HRAS | Cellular senescence | pathway |
| ACHE | CAT | ppi | EGFR | EGFR tyrosine kinase inhibitor resistance | pathway |
| CXCL8 | EGFR | ppi | VEGFA | EGFR tyrosine kinase inhibitor resistance | pathway |
| IGF1 | TP53 | ppi | BAX | EGFR tyrosine kinase inhibitor resistance | pathway |
| PTGS2 | FOS | ppi | IL6 | EGFR tyrosine kinase inhibitor resistance | pathway |
| CCNA2 | EP300 | ppi | IGF1R | EGFR tyrosine kinase inhibitor resistance | pathway |
| INS | CXCL8 | ppi | IGF1 | EGFR tyrosine kinase inhibitor resistance | pathway |
| CXCL8 | IGF1 | ppi | AKT1 | EGFR tyrosine kinase inhibitor resistance | pathway |
| AKT1 | FOS | ppi | KDR | EGFR tyrosine kinase inhibitor resistance | pathway |
| LDLR | APP | ppi | STAT3 | EGFR tyrosine kinase inhibitor resistance | pathway |
| INS | FOS | ppi | RAF1 | EGFR tyrosine kinase inhibitor resistance | pathway |
| NOS2 | NOS3 | ppi | PTEN | EGFR tyrosine kinase inhibitor resistance | pathway |
| CXCL8 | TIMP1 | ppi | BRAF | EGFR tyrosine kinase inhibitor resistance | pathway |
| HRAS | JUN | ppi | HRAS | EGFR tyrosine kinase inhibitor resistance | pathway |
| CCL5 | IL4 | ppi | CDKN1A | Prostate cancer | pathway |
| EGFR | IL2 | ppi | TP53 | Prostate cancer | pathway |
| PDE3A | PRKACA | ppi | IGF1R | Prostate cancer | pathway |
| EGFR | MAPK14 | ppi | EP300 | Prostate cancer | pathway |
| XIAP | RAF1 | ppi | IGF1 | Prostate cancer | pathway |
| CHEK2 | CCNA2 | ppi | AKT1 | Prostate cancer | pathway |
| IL6 | SELE | ppi | HRAS | Prostate cancer | pathway |
| FN1 | CCL2 | ppi | FGFR1 | Prostate cancer | pathway |
| FOS | IL1B | ppi | MDM2 | Prostate cancer | pathway |
| ELANE | MMP9 | ppi | RAF1 | Prostate cancer | pathway |
| HRAS | STAT3 | ppi | PTEN | Prostate cancer | pathway |
| LDLR | INS | ppi | INS | Prostate cancer | pathway |
| IGF1 | STAT3 | ppi | KLK3 | Prostate cancer | pathway |
| FGFR1 | INS | ppi | BRAF | Prostate cancer | pathway |
| CCL5 | MMP9 | ppi | EGFR | Prostate cancer | pathway |
| PTGS2 | IL4 | ppi | HSP90AA1 | Prostate cancer | pathway |
| FN1 | CXCL8 | ppi | MMP9 | Prostate cancer | pathway |
| MMP9 | TP53 | ppi | EGFR | Proteoglycans in cancer | pathway |
| PRKACA | BAX | ppi | MAPK14 | Proteoglycans in cancer | pathway |
| TNF | APP | ppi | PRKACA | Proteoglycans in cancer | pathway |
| IL6 | NOS3 | ppi | VEGFA | Proteoglycans in cancer | pathway |
| SELE | IL1B | ppi | CDKN1A | Proteoglycans in cancer | pathway |
| MDM2 | CDK4 | ppi | TNF | Proteoglycans in cancer | pathway |
| PTEN | KDR | ppi | CASP3 | Proteoglycans in cancer | pathway |
| IL6 | REN | ppi | TP53 | Proteoglycans in cancer | pathway |
| FOS | IGF1 | ppi | IGF1R | Proteoglycans in cancer | pathway |
| HSP90AA1 | P4HB | ppi | IGF1 | Proteoglycans in cancer | pathway |
| CCL5 | IL2 | ppi | AKT1 | Proteoglycans in cancer | pathway |
| IL2 | CCL2 | ppi | HRAS | Proteoglycans in cancer | pathway |
| AKT1 | CCL2 | ppi | KDR | Proteoglycans in cancer | pathway |
| ELANE | IL6 | ppi | FGFR1 | Proteoglycans in cancer | pathway |
| INS | ICAM1 | ppi | STAT3 | Proteoglycans in cancer | pathway |
| INS | IL4 | ppi | MMP9 | Proteoglycans in cancer | pathway |
| CASP3 | NOS3 | ppi | MDM2 | Proteoglycans in cancer | pathway |
| INS | MMP9 | ppi | RAF1 | Proteoglycans in cancer | pathway |
| STAT3 | KDR | ppi | HIF1A | Proteoglycans in cancer | pathway |
| FGFR1 | IGF1 | ppi | FN1 | Proteoglycans in cancer | pathway |
| SELE | CXCL8 | ppi | TGFB1 | Proteoglycans in cancer | pathway |
| TNF | TGFB1 | ppi | TGFB2 | Proteoglycans in cancer | pathway |
| PTGS2 | MAPK14 | ppi | BRAF | Proteoglycans in cancer | pathway |
| PTEN | CDK4 | ppi | CDC42 | Proteoglycans in cancer | pathway |
| MMP9 | NOS3 | ppi | EGFR | Melanoma | pathway |
| SOD2 | TNF | ppi | CDKN1A | Melanoma | pathway |
| CYP19A1 | UGT1A1 | ppi | TP53 | Melanoma | pathway |
| CASP3 | CAT | ppi | IGF1R | Melanoma | pathway |
| INS | CASP3 | ppi | IGF1 | Melanoma | pathway |
| CDKN2A | EGFR | ppi | AKT1 | Melanoma | pathway |
| EGFR | IL4 | ppi | HRAS | Melanoma | pathway |
| PTEN | CASP3 | ppi | FGFR1 | Melanoma | pathway |
| AKT1 | CCNA2 | ppi | MDM2 | Melanoma | pathway |
| IGF1 | IL1B | ppi | RAF1 | Melanoma | pathway |
| CXCL8 | KDR | ppi | BRAF | Melanoma | pathway |
| IL6 | CASP3 | ppi | PTEN | Melanoma | pathway |
| ADRB2 | IL1B | ppi | CDK4 | Melanoma | pathway |
| MME | APP | ppi | CDKN2A | Melanoma | pathway |
| MTTP | INS | ppi | BAX | Melanoma | pathway |
| AKT1 | CDKN2A | ppi | EGFR | Glioma | pathway |
| JUN | FN1 | ppi | CDKN1A | Glioma | pathway |
| SOD2 | IL6 | ppi | TP53 | Glioma | pathway |
| VEGFA | CDKN2A | ppi | IGF1R | Glioma | pathway |
| SELE | CCL2 | ppi | IGF1 | Glioma | pathway |
| FN1 | IL1B | ppi | AKT1 | Glioma | pathway |
| HIF1A | PTEN | ppi | HRAS | Glioma | pathway |
| JUN | ICAM1 | ppi | MDM2 | Glioma | pathway |
| JUN | IGF1 | ppi | RAF1 | Glioma | pathway |
| CDKN2A | IL6 | ppi | BRAF | Glioma | pathway |
| CASP3 | EGFR | ppi | PTEN | Glioma | pathway |
| HSP90AA1 | BRAF | ppi | CDK4 | Glioma | pathway |
| CXCL8 | FOS | ppi | CDKN2A | Glioma | pathway |
| FN1 | NOS3 | ppi | CALM1 | Glioma | pathway |
| PTGS2 | IGF1 | ppi | CALM2 | Glioma | pathway |
| XIAP | MDM2 | ppi | BAX | Glioma | pathway |
| AKT1 | TIMP1 | ppi | TP53 | Pancreatic cancer | pathway |
| NOS3 | CAT | ppi | AKT1 | Pancreatic cancer | pathway |
| CXCL8 | CFTR | ppi | STAT3 | Pancreatic cancer | pathway |
| PTGS2 | FN1 | ppi | RAF1 | Pancreatic cancer | pathway |
| FN1 | F2 | ppi | TGFB1 | Pancreatic cancer | pathway |
| SELE | IL4 | ppi | TGFB2 | Pancreatic cancer | pathway |
| CDC42 | CASP3 | ppi | BRAF | Pancreatic cancer | pathway |
| IL6 | NOS2 | ppi | CDC42 | Pancreatic cancer | pathway |
| MMP9 | PTGS2 | ppi | CDK4 | Pancreatic cancer | pathway |
| TNF | REN | ppi | CDKN2A | Pancreatic cancer | pathway |
| INS | CAT | ppi | VEGFA | Pancreatic cancer | pathway |
| CDKN2A | STAT3 | ppi | BAX | Pancreatic cancer | pathway |
| IGF1 | CCL2 | ppi | CDKN1A | Pancreatic cancer | pathway |
| VEGFA | CASP3 | ppi | EGFR | Pancreatic cancer | pathway |
| MMP9 | ICAM1 | ppi | RAF1 | Bladder cancer | pathway |
| INS | APOA1 | ppi | VEGFA | Bladder cancer | pathway |
| IL1B | TGFB1 | ppi | MMP9 | Bladder cancer | pathway |
| MMP9 | PTEN | ppi | BRAF | Bladder cancer | pathway |
| IL6 | CAT | ppi | CDK4 | Bladder cancer | pathway |
| IGF1 | NOS3 | ppi | CDKN2A | Bladder cancer | pathway |
| AKT1 | BAX | ppi | CDKN1A | Bladder cancer | pathway |
| HSP90AA1 | CAT | ppi | TP53 | Bladder cancer | pathway |
| MME | DNTT | ppi | HRAS | Bladder cancer | pathway |
| VEGFA | IL2 | ppi | CXCL8 | Bladder cancer | pathway |
| HRAS | CDK4 | ppi | EGFR | Bladder cancer | pathway |
| HIF1A | INS | ppi | MDM2 | Bladder cancer | pathway |
| IL6 | KLK3 | ppi | IL6 | Chagas disease (American trypanosomiasis) | pathway |
| NOS3 | TP53 | ppi | TNF | Chagas disease (American trypanosomiasis) | pathway |
| TP53 | ICAM1 | ppi | CCL2 | Chagas disease (American trypanosomiasis) | pathway |
| INS | TP53 | ppi | CXCL8 | Chagas disease (American trypanosomiasis) | pathway |
| HRAS | MMP9 | ppi | CCL5 | Chagas disease (American trypanosomiasis) | pathway |
| PTEN | BRAF | ppi | TGFB1 | Chagas disease (American trypanosomiasis) | pathway |
| HSP90AA1 | CFTR | ppi | AKT1 | Chagas disease (American trypanosomiasis) | pathway |
| NOS3 | IL1B | ppi | FOS | Chagas disease (American trypanosomiasis) | pathway |
| ELANE | ICAM1 | ppi | JUN | Chagas disease (American trypanosomiasis) | pathway |
| HRAS | KDR | ppi | MAPK14 | Chagas disease (American trypanosomiasis) | pathway |
| TNF | KDR | ppi | IL1B | Chagas disease (American trypanosomiasis) | pathway |
| VEGFA | IL4 | ppi | NOS2 | Chagas disease (American trypanosomiasis) | pathway |
| FOS | IL4 | ppi | IL2 | Chagas disease (American trypanosomiasis) | pathway |
| INS | IL1B | ppi | TGFB2 | Chagas disease (American trypanosomiasis) | pathway |
| KIT | IL4 | ppi | EGFR | Human cytomegalovirus infection | pathway |
| TP53 | CCL2 | ppi | PTGS2 | Human cytomegalovirus infection | pathway |
| IL6 | F2 | ppi | MAPK14 | Human cytomegalovirus infection | pathway |
| XIAP | TP53 | ppi | PRKACA | Human cytomegalovirus infection | pathway |
| MMP9 | CASP3 | ppi | VEGFA | Human cytomegalovirus infection | pathway |
| HRAS | PTGS2 | ppi | CDKN1A | Human cytomegalovirus infection | pathway |
| HRAS | CALM1 | ppi | BAX | Human cytomegalovirus infection | pathway |
| ELANE | CASP3 | ppi | TNF | Human cytomegalovirus infection | pathway |
| CCL2 | TIMP1 | ppi | IL6 | Human cytomegalovirus infection | pathway |
| HRAS | CDKN1A | ppi | CASP3 | Human cytomegalovirus infection | pathway |
| APP | EGFR | ppi | TP53 | Human cytomegalovirus infection | pathway |
| TNF | PTEN | ppi | CALM1 | Human cytomegalovirus infection | pathway |
| UGT1A1 | HSD11B1 | ppi | CALM2 | Human cytomegalovirus infection | pathway |
| TNF | IGF1 | ppi | AKT1 | Human cytomegalovirus infection | pathway |
| APP | TP53 | ppi | STAT3 | Human cytomegalovirus infection | pathway |
| TNF | EGFR | ppi | CDKN2A | Human cytomegalovirus infection | pathway |
| PTGS2 | CAT | ppi | MDM2 | Human cytomegalovirus infection | pathway |
| AKT1 | TGFB1 | ppi | RAF1 | Human cytomegalovirus infection | pathway |
| SOD2 | JUN | ppi | IL1B | Human cytomegalovirus infection | pathway |
| VEGFA | MDM2 | ppi | CCL2 | Human cytomegalovirus infection | pathway |
| INS | CYP3A4 | ppi | CXCL8 | Human cytomegalovirus infection | pathway |
| JUN | APP | ppi | CCL5 | Human cytomegalovirus infection | pathway |
| CHEK2 | PTEN | ppi | CDK4 | Human cytomegalovirus infection | pathway |
| SOD2 | NOS3 | ppi | HRAS | Human cytomegalovirus infection | pathway |
| JUN | CCNA2 | ppi | PTGS2 | Kaposi sarcoma-associated herpesvirus infection | pathway |
| JUN | PTGS2 | ppi | MAPK14 | Kaposi sarcoma-associated herpesvirus infection | pathway |
| FN1 | KLK3 | ppi | VEGFA | Kaposi sarcoma-associated herpesvirus infection | pathway |
| HRAS | CXCL8 | ppi | FOS | Kaposi sarcoma-associated herpesvirus infection | pathway |
| IL6 | HSP90AA1 | ppi | CDKN1A | Kaposi sarcoma-associated herpesvirus infection | pathway |
| HMGCR | APOB | ppi | BAX | Kaposi sarcoma-associated herpesvirus infection | pathway |
| MME | KIT | ppi | JUN | Kaposi sarcoma-associated herpesvirus infection | pathway |
| DNTT | TP53 | ppi | IL6 | Kaposi sarcoma-associated herpesvirus infection | pathway |
| HSP90AA1 | NOS2 | ppi | CASP3 | Kaposi sarcoma-associated herpesvirus infection | pathway |
| ELANE | PTGS2 | ppi | TP53 | Kaposi sarcoma-associated herpesvirus infection | pathway |
| AKT1 | CXCL8 | ppi | ICAM1 | Kaposi sarcoma-associated herpesvirus infection | pathway |
| HSP90AA1 | MDM2 | ppi | CALM1 | Kaposi sarcoma-associated herpesvirus infection | pathway |
| HIF1A | IGF1 | ppi | CALM2 | Kaposi sarcoma-associated herpesvirus infection | pathway |
| NOS3 | ICAM1 | ppi | EP300 | Kaposi sarcoma-associated herpesvirus infection | pathway |
| CDKN2A | CCNA2 | ppi | AKT1 | Kaposi sarcoma-associated herpesvirus infection | pathway |
| PTGS2 | ICAM1 | ppi | STAT3 | Kaposi sarcoma-associated herpesvirus infection | pathway |
| CDKN1A | CHEK2 | ppi | RAF1 | Kaposi sarcoma-associated herpesvirus infection | pathway |
| XIAP | BAX | ppi | HIF1A | Kaposi sarcoma-associated herpesvirus infection | pathway |
| ZAP70 | IL2 | ppi | CXCL8 | Kaposi sarcoma-associated herpesvirus infection | pathway |
| FN1 | KDR | ppi | CDK4 | Kaposi sarcoma-associated herpesvirus infection | pathway |
| IL4 | TGFB1 | ppi | HRAS | Kaposi sarcoma-associated herpesvirus infection | pathway |
| CDC42 | JUN | ppi | EGFR | Breast cancer | pathway |
| NS | TBXAS1 | disease | FOS | Breast cancer | pathway |
| NS | MAOA | disease | CDKN1A | Breast cancer | pathway |
| NS | EGFR | disease | BAX | Breast cancer | pathway |
| NS | SHMT1 | disease | JUN | Breast cancer | pathway |
| NS | PTGS2 | disease | TP53 | Breast cancer | pathway |
| NS | ADRB2 | disease | IGF1R | Breast cancer | pathway |
| NS | MAPK14 | disease | IGF1 | Breast cancer | pathway |
| NS | HSP90AA1 | disease | AKT1 | Breast cancer | pathway |
| NS | PRKACA | disease | FGFR1 | Breast cancer | pathway |
| NS | PDE3A | disease | RAF1 | Breast cancer | pathway |
| NS | VEGFA | disease | PTEN | Breast cancer | pathway |
| NS | FOS | disease | KIT | Breast cancer | pathway |
| NS | CDKN1A | disease | BRAF | Breast cancer | pathway |
| NS | BAX | disease | CDK4 | Breast cancer | pathway |
| NS | TNF | disease | HRAS | Breast cancer | pathway |
